# Supplementary material for: A mosquito salivary protein-driven influx of myeloid cells facilitates flavivirus transmission
Source: EMBO J. 2024 Feb 20;43(9):2. doi: 10.1038/s44318-024-00056-x (PMC11066113; doi:10.1038/s44318-024-00056-x)
Supplement: Supplementary file 1 — Appendix [file 44318_2024_56_MOESM1_ESM.pdf]

Appendix Supplementary Information for

A mosquito salivary protein-driven influx of myeloid cells facilitates flavivirus transmission

Zhaoyang Wang, Kaixiao Nie, Yan Liang, Jichen Niu, Xi Yu, Oujia Zhang, Long Liu, Xiaolu Shi, Yibaina Wang, Xuechun Feng, Yibin Zhu, Penghua Wang, Gong Cheng

Contents

Appendix Figure S1 (Page 2)

Appendix Figure S2 (Page 3)

Appendix Figure S3 (Page 4)

Appendix Figure S4 (Page 5)

Appendix Figure S5 (Page 6)

Appendix Figure S6 (Page 7)

Appendix Table S1 (Pages 8 and 9)

Appendix Table S2 (Pages 10 -15)

Appendix Table S3 (Pages 16 - 18)

Appendix Table S4 (Page 19)

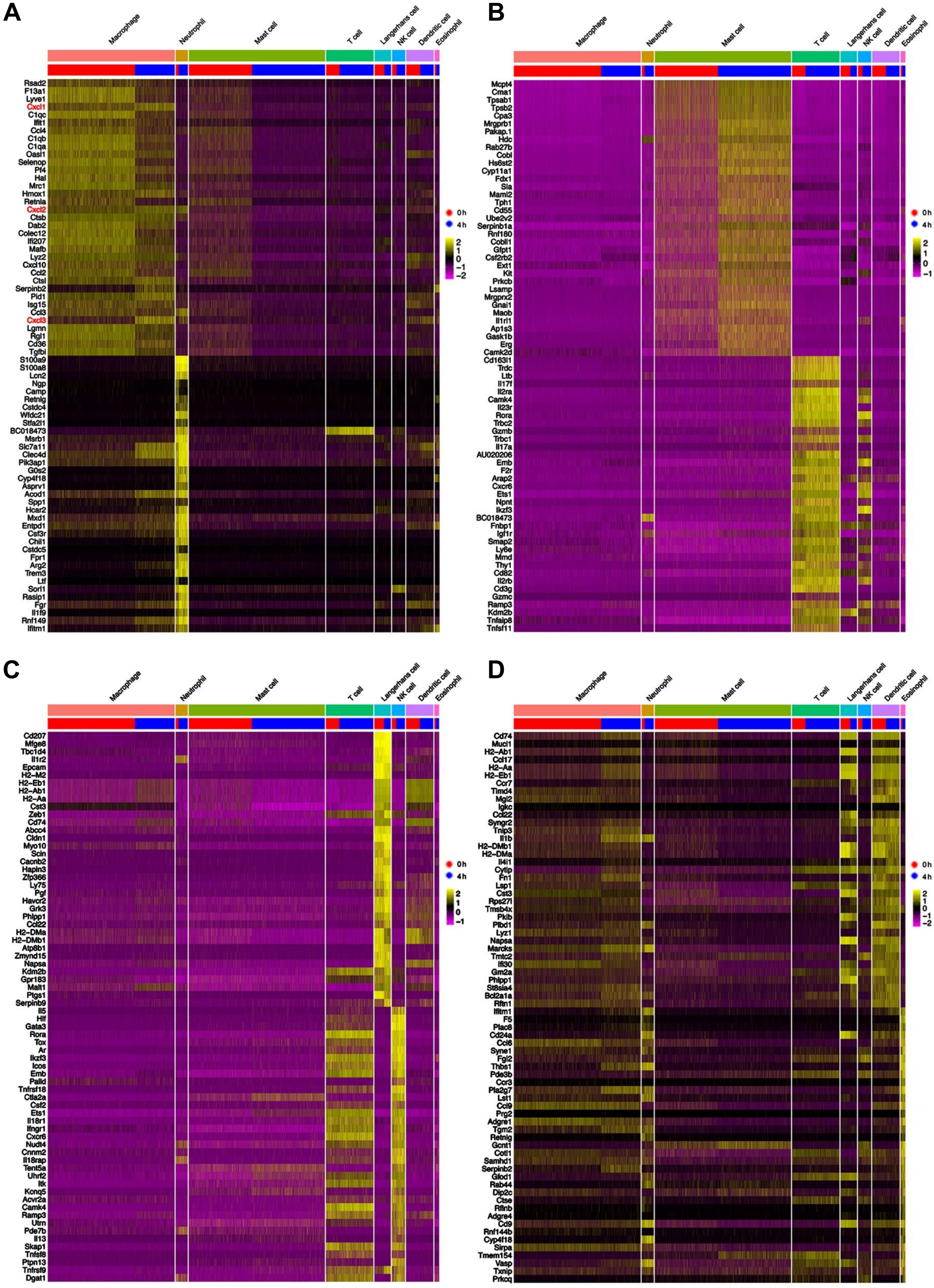

Appendix Fig S1

Appendix Figure S1. Heatmap of the expression levels of the top 35 differentially expressed genes (DEGs) in different types of skin resident immunocytes, related to Fig. 2.

(A – D) The heatmap displays the relative expression levels of the top 35 DEGs in skin macrophages and neutrophils (A), mast cells and T cells (B), Langerhans cells and natural killer cells (C), dendritic cells and eosinophils (D) at both the 0 h and 4 h time points, using a common scale for comparison. The top DEGs in each type of skin immunocytes are well clustered and scaled with yellow color denoting the higher expression levels. CXCL1/2/3 (marked in red font) were almost exclusively and highly expressed by the skin macrophages, and their expression was significantly induced by mosquito bite. The original scRNA-Seq data is available at NCBI-GEO with the accession number GSE232756.

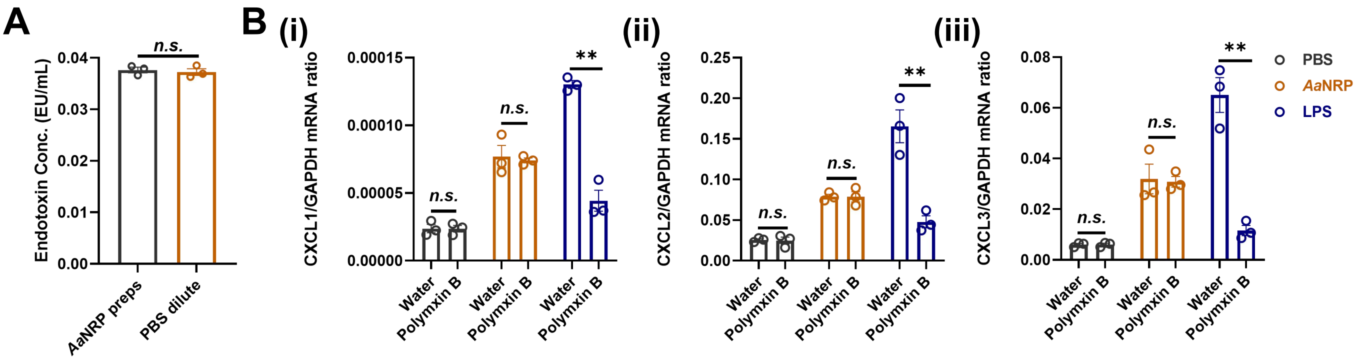

Appendix Fig S2

Appendix Figure S2. Exclusion of endotoxin/LPS contamination in purified *Aa*NRP.

(A) Determination of endotoxin in purified *Aa*NRP preparation and PBS dilute (Gibco, Cat# 10010023). The content of endotoxin was determined by using Pierce LAL Chromogenic Endotoxin Quantitation Kit (Cat# A39552S). The endotoxin concentration in the preparation of purified *Aa*NRP protein was very low (<0.1 EU/ml) and showed no difference with the PBS solution (Average level: *Aa*NRP 0.038 EU/ml, PBS 0.037 EU/ml, p=0.66). It is generally believed that an endotoxin content below 0.1 EU/ml is an excellent condition, endotoxin at this concentration has no detectable effect on the cultured cells or animals.

(B) Influence of polymyxin B preincubation on *Aa*NRP and LPS stimulated CXCL1/2/3 expression. The purified *Aa*NRP and LPS were incubated with sterile water (control) or with 30 μg/ml polymyxin B (neutralization) at 4°C for 24 hours. Then the sterile water or polymyxin B-treated *Aa*NRP and LPS were added to murine RAW264.7 macrophages for incubation. Four hours later, the cells were collected for qPCR detection of CXCL1/2/3 expression.

(A and B) Data are expressed as the mean ± SEM and each dot represents an independent replicate. The unpaired t test (A) and two-way ANOVA were used for statistical analyses. ** p<0.01, *n.s.* not significant.

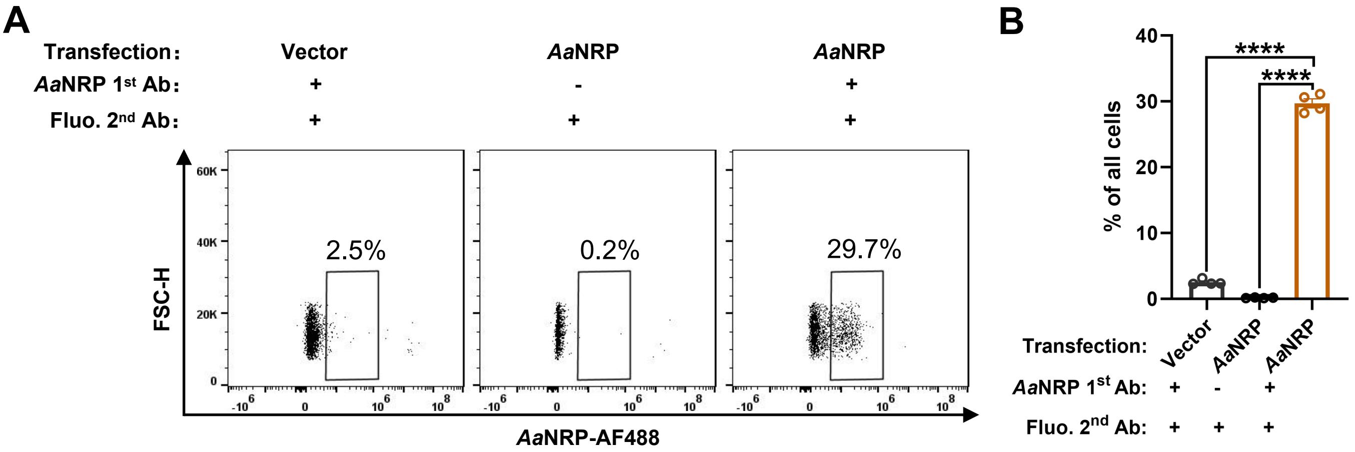

Appendix Fig S3

Appendix Figure S3. Validation of the specificity of *Aa*NRP antibody (Ab).

(A) Dot plots showing the *Aa*NRP Ab-bound cells that are AF488 positive.

(B) Percentages of the *Aa*NRP Ab-bound cells. HEK293T cells were transfected with vector or *A. aegypti Aa*NRP plasmid. Forty-eight hours later, the vector- or *Aa*NRP-transfected HEK293T cells were incubated with or without *Aa*NRP Ab, followed by incubation with AF488-labeled secondary Ab. The Ab-bound cells were detected by flow cytometry. Data are expressed as the mean ± SEM, and each dot represents an independent replicate. One-way ANOVA and multiple t tests were used for statistical analyses. **** $p<0.0001$.

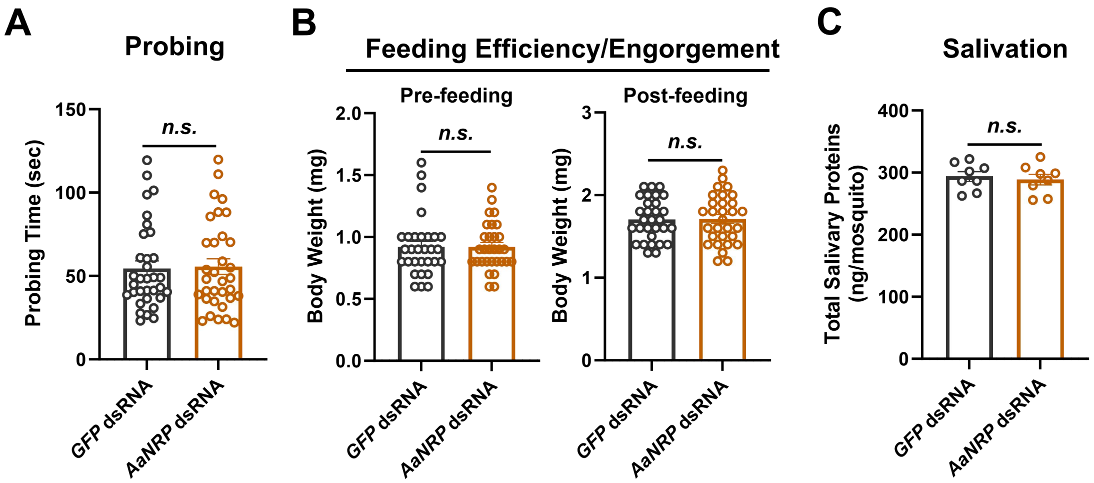

Appendix Figure S4

Appendix Figure S4. *AaNRP* silencing has no influence on the efficiency of probing, feeding or salivation of *A. aegypti*. (A) Probing time of *GFP* dsRNA- and *AaNRP* dsRNA-treated mosquitoes. *AaNRP* dsRNA was intrathoracically inoculated into the female mosquitoes. The mosquitoes inoculated by *GFP* dsRNA served as negative controls. Three days after administration of dsRNA, the mosquitoes were intrathoracically infected with ZIKV. At 8 days after infection, these mosquitoes were allowed to bite anesthetized mice. Probing time is defined as the interval from the initial insertion of the mosquito mouthparts into the skin until visualization of first traces of blood in the midgut (Martin-Martin *et al*, 2022). (B) Feeding efficiency of *GFP* dsRNA- and *AaNRP* dsRNA-treated mosquitoes. The body weight of each mosquito was recorded immediately before and post blood feeding on anesthetized mice. (C) The amount of total protein components in mosquito saliva. Mosquito saliva was collected by forced salivation (Miller *et al*, 2021) and the total salivary proteins were quantified by Bradford assay.

(A – C) Data are expressed as the mean ± SEM and each dot represents an individual mouse. The unpaired t tests were used for statistical analyses.

All experiments were reproduced at least twice. *n.s.* not significant.

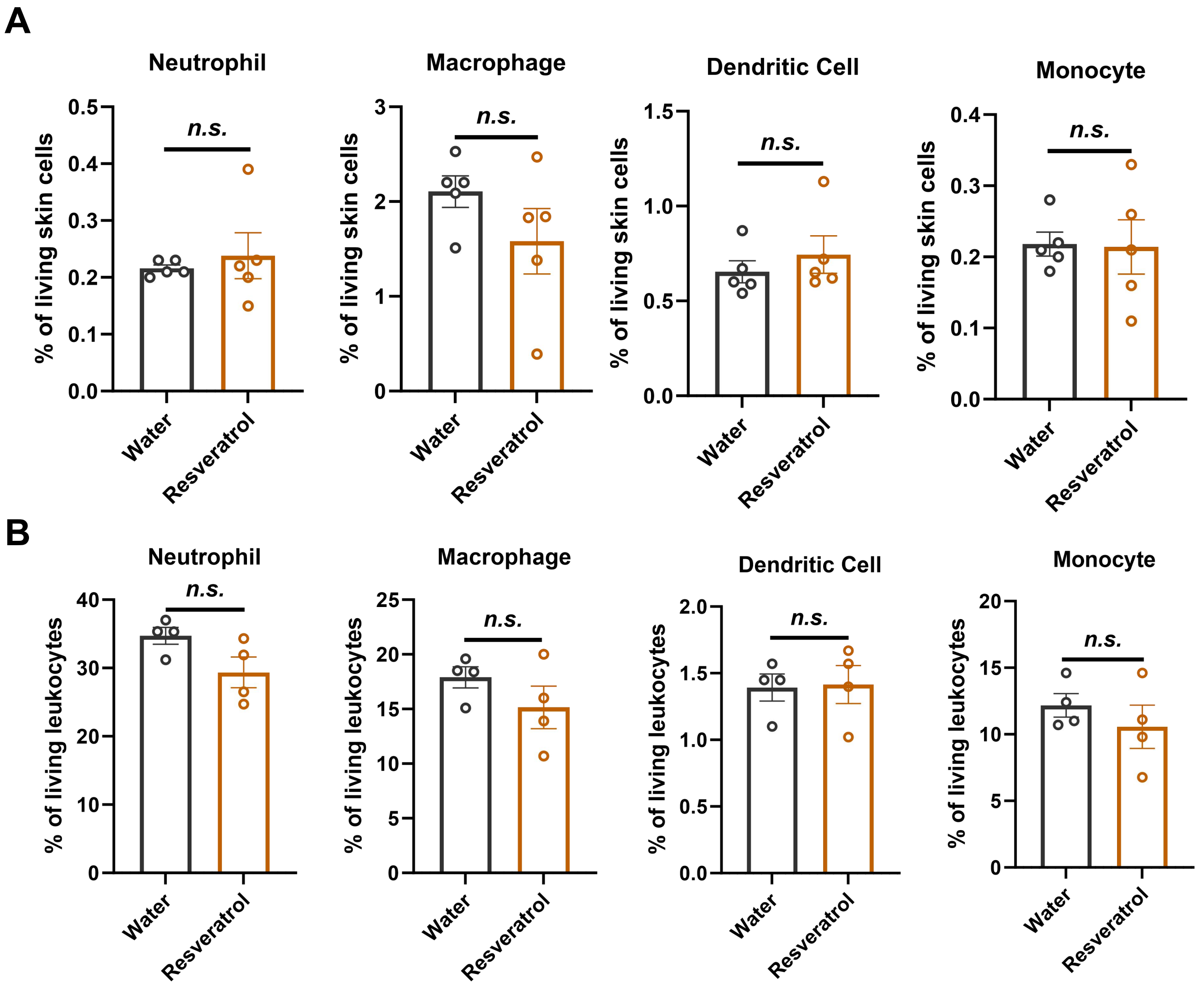

Appendix Figure S5

Appendix Figure S5. Influence of resveratrol supplementation on the proportions of myeloid cells in resting skin and blood.

(A-B) Percentages of neutrophils, macrophages, dendritic cells (DCs) and monocytes in murine footpad skin (A) and peripheral blood (B) of water-administrated and resveratrol-administrated mice. Six-week-old A129 mice were orally administered 200 μL of 20 mg/mL resveratrol once daily for 14 days. One hour post the last administration, mice were euthanized for analyzing the proportions of neutrophils, macrophages, dendritic cells and monocytes in footpad skin (A) and blood (B) by flow cytometry.

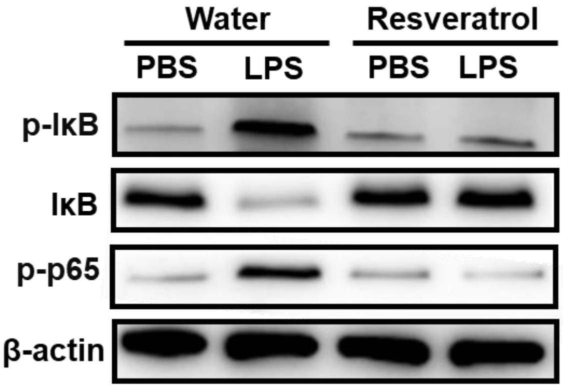

Appendix Figure S6

Appendix Figure S6. Oral gavage of resveratrol attenuated LPS-activated MyD88-NF-κB signaling in murine footpad skin. Six-week-old A129 mice were orally administered water or 4.0 mg resveratrol (Res) once daily for 14 days. Afterward, A129 mice were subcutaneously injected with ZIKV together with PBS or LPS (100 ng/ml) immediately after the last dose of resveratrol. Four hours later, mouse footpads were collected for detecting the activation of MyD88-NF-κB signaling by immunoblotting assay.

Appendix Table S1. ***Aedes aegypti*** **salivary proteins identified by LC-MS/MS with the spectrometric score greater than 25.**

Gene ID	Gene Name	Score
AAEL006347	Apyrase Precursor	8418.65
AAEL006485	Inosine-uridine preferring nucleoside hydrolase	2413.71
AAEL006424	37 kDa salivary gland allergen Aed a 2 Precursor	2262.26
AAEL003182	Serpin homologue	2202.37
AAEL000749	Putative protein	1852.42
AAEL006417	D7 protein	1468.68
AAEL010235	30 kDa salivary gland allergen Aed a 3 Precursor	1264.15
AAEL000726	Fibrinogen and fibronectin	1260.68
AAEL000556	C-Type Lectin	1247.66
AAEL006096	Gelsolin precursor	1049.90
AAEL002704	Serpin homologue	922.50
AAEL005672	Adenosine deaminase	878.16
AAEL007420	Serpin homologue	865.74
AAEL000533	C-Type Lectin	865.43
AAEL000748	Putative protein	801.34
AAEL010228	Putative protein	736.52
AAEL000732	Putative protein	585.68
AAEL003600	Putative protein	425.26
AAEL000793	Venom allergen	417.39
AAEL003053	Allergen	416.16
AAEL003601	Putative protein	376.62
AAEL013532	Putative protein	329.88

AAEL003585	Putative protein	243.14
AAEL009524	Alpha-amylase	231.66
AAEL000392	Probable maltase Precursor	230.17
AAEL009852	Putative protein	182.64
AAEL013029	Deoxyribonuclease I	138.92
AAEL011510	Multiple inositol polyphosphate phosphatase	113.65
AAEL009081	Putative protein	106.93
AAEL003057	Allergen	102.74
AAEL008305	Putative protein	82.93
AAEL007986	Putative protein	78.71
AAEL002693	Venom allergen	78.02
AAEL006423	Putative protein	75.81
AAEL009670	C-Type Lysozyme	75.38
AAEL003584	Putative protein	52.68
AAEL007064	Gram-Negative Binding Protein	52.19
AAEL006406	Putative protein	47.87
AAEL018258	Putative protein	45.45
AAEL007394	Putative protein	42.91
AAEL003107	Putative protein	40.78
AAEL006351	Putative protein	27.61

Appendix Table S2. Top150 upregulated genes in skin resident macrophages (4 h *v.s.* 0 h).

Gene Name	Gene ID	Fold Change	p-value
Thbs1	ENSMUSG00000040152	6.67	3.04E-198
Serpinb2	ENSMUSG00000062345	5.32	9.34E-165
Ptgs2	ENSMUSG00000032487	4.25	1.20E-261
Slc7a11	ENSMUSG00000027737	3.66	0
Slpi	ENSMUSG00000017002	3.63	7.30E-75
Cxcl3	ENSMUSG00000029379	3.61	2.29E-232
Vcan	ENSMUSG00000021614	3.54	9.08E-151
Nrg1	ENSMUSG00000062991	3.21	4.25E-168
Il1b	ENSMUSG00000027398	3.02	1.27E-193
Inhba	ENSMUSG00000041324	2.94	9.85E-121
Timp1	ENSMUSG00000001131	2.91	1.34E-146
Il7r	ENSMUSG00000003882	2.68	9.57E-222
Esd	ENSMUSG00000021996	2.62	1.04E-226
Sod2	ENSMUSG00000006818	2.61	2.93E-248
Clec4e	ENSMUSG00000030142	2.57	1.20E-232
F10	ENSMUSG00000031444	2.54	6.84E-152
Cd14	ENSMUSG00000051439	2.46	1.45E-211
Fth1	ENSMUSG00000024661	2.40	4.04E-277
Lmo4	ENSMUSG00000028266	2.32	3.81E-99
Clec4d	ENSMUSG00000030144	2.29	1.27E-211
Csf2rb	ENSMUSG00000071713	2.27	2.89E-196
Mmp14	ENSMUSG00000000957	2.21	8.32E-146
Plcb1	ENSMUSG00000051177	2.19	9.85E-90
Csf3	ENSMUSG00000038067	2.16	7.93E-93
Acod1	ENSMUSG00000022126	2.15	7.91E-127
Il1rn	ENSMUSG00000026981	2.12	2.14E-139

Smox	ENSMUSG00000027333	2.10	8.95E-186
Cytip	ENSMUSG00000026832	2.07	9.69E-168
H2-Ab1	ENSMUSG00000073421	2.03	2.58E-125
Flrt3	ENSMUSG00000051379	2.02	5.22E-195
Pla2g7	ENSMUSG00000023913	2.00	5.30E-137
Txnrd1	ENSMUSG00000020250	1.99	1.16E-235
Srgn	ENSMUSG00000020077	1.99	1.36E-179
Cd74	ENSMUSG00000024610	1.97	1.97E-131
Malt1	ENSMUSG00000032688	1.97	2.74E-142
H2-Aa	ENSMUSG00000036594	1.93	1.79E-110
Ifitm1	ENSMUSG00000025491	1.93	2.41E-78
Procr	ENSMUSG00000027611	1.91	1.17E-144
H2-Eb1	ENSMUSG00000060586	1.90	8.74E-104
Mir155hg	ENSMUSG00000097418	1.88	1.18E-91
Traf1	ENSMUSG00000026875	1.87	2.24E-155
Pid1	ENSMUSG00000045658	1.86	7.02E-25
Bcl2a1d	ENSMUSG00000099974	1.86	2.50E-115
Rab11fip1	ENSMUSG00000031488	1.85	3.19E-171
Ccl5	ENSMUSG00000035042	1.84	1.87E-13
Mthfd2	ENSMUSG00000005667	1.82	4.36E-154
Samsn1	ENSMUSG00000022876	1.82	1.70E-82
Errfi1	ENSMUSG00000028967	1.81	3.26E-101
Srxn1	ENSMUSG00000032802	1.80	3.05E-89
Il1a	ENSMUSG00000027399	1.80	6.16E-41
Gclm	ENSMUSG00000028124	1.79	1.88E-85
Bcl2a1b	ENSMUSG00000089929	1.78	1.04E-105
Hif1a	ENSMUSG00000021109	1.77	1.61E-88
Tnfrsf1b	ENSMUSG00000028599	1.77	1.10E-139
Met	ENSMUSG00000009376	1.76	8.57E-125

Ndrg1	ENSMUSG00000005125	1.76	7.88E-80
Hmox1	ENSMUSG00000005413	1.75	1.79E-08
Ramp3	ENSMUSG00000041046	1.74	2.10E-83
S100a9	ENSMUSG00000056071	1.74	4.59E-22
Marcks	ENSMUSG00000069662	1.74	9.65E-109
Tmtc2	ENSMUSG00000036019	1.72	3.71E-73
Il10	ENSMUSG00000016529	1.71	1.30E-35
Prdx1	ENSMUSG00000028691	1.71	1.41E-21
Clec4n	ENSMUSG00000023349	1.71	1.34E-55
Plet1	ENSMUSG00000032068	1.71	3.16E-18
Eif2s2	ENSMUSG00000074656	1.70	4.75E-167
Antxr2	ENSMUSG00000029338	1.70	5.62E-35
Mmp19	ENSMUSG00000025355	1.69	4.96E-14
Map4k4	ENSMUSG00000026074	1.69	5.41E-112
Cmss1	ENSMUSG00000022748	1.68	6.38E-122
Pdpn	ENSMUSG00000028583	1.67	2.42E-59
Bcl2a1a	ENSMUSG00000102037	1.66	7.85E-103
Ptges	ENSMUSG00000050737	1.66	2.35E-70
AY036118	ENSMUSG00000105361	1.64	3.37E-97
Ctsl	ENSMUSG00000021477	1.64	5.68E-10
Btg1	ENSMUSG00000036478	1.64	8.94E-102
Id2	ENSMUSG00000020644	1.63	7.90E-110
Sipa1l1	ENSMUSG00000042700	1.62	2.57E-54
Tnip3	ENSMUSG00000044162	1.62	3.06E-88
Fyb	ENSMUSG00000022148	1.61	3.42E-52
Denr	ENSMUSG00000023106	1.61	1.46E-57
Csf2rb2	ENSMUSG00000071714	1.61	2.52E-155
Cd274	ENSMUSG00000016496	1.61	3.06E-100
Ifitm3	ENSMUSG00000025492	1.61	7.45E-10

Basp1	ENSMUSG00000045763	1.59	9.84E-76
Slco3a1	ENSMUSG00000025790	1.58	9.00E-51
Slc7a2	ENSMUSG00000031596	1.58	7.11E-54
Txn1	ENSMUSG00000028367	1.58	7.11E-100
Rnf149	ENSMUSG00000048234	1.58	3.76E-95
Lars2	ENSMUSG00000035202	1.57	8.96E-67
Fn1	ENSMUSG00000026193	1.57	8.14E-73
Cacna1d	ENSMUSG00000015968	1.57	1.54E-34
Slc15a3	ENSMUSG00000024737	1.57	1.37E-99
Cxcl1	ENSMUSG00000029380	1.56	1.13E-32
Ccnd2	ENSMUSG00000000184	1.56	1.81E-25
Ccr7	ENSMUSG00000037944	1.56	9.95E-37
Stap1	ENSMUSG00000029254	1.56	1.34E-58
Ppp4r2	ENSMUSG00000052144	1.55	4.53E-118
Lcp1	ENSMUSG00000021998	1.55	2.36E-70
Adam8	ENSMUSG00000025473	1.55	9.70E-131
Il1r1	ENSMUSG00000026072	1.55	7.99E-76
Tax1bp1	ENSMUSG00000004535	1.55	1.22E-108
Ms4a4c	ENSMUSG00000024675	1.54	2.99E-65
Slc39a14	ENSMUSG00000022094	1.54	1.53E-106
Gpr141	ENSMUSG00000053101	1.54	1.60E-87
Tnip1	ENSMUSG00000020400	1.53	3.80E-96
Il4i1	ENSMUSG00000074141	1.53	2.08E-50
H2afz	ENSMUSG00000037894	1.52	1.59E-55
Snx18	ENSMUSG00000042364	1.52	7.05E-107
Zeb1	ENSMUSG00000024238	1.52	4.65E-49
Cxcl2	ENSMUSG00000058427	1.51	4.04E-33
Cd52	ENSMUSG00000000682	1.51	9.34E-72
Jak2	ENSMUSG00000024789	1.51	2.33E-74

Ell2	ENSMUSG00000001542	1.50	3.77E-87
Mefv	ENSMUSG00000022534	1.50	1.49E-129
Phlda1	ENSMUSG00000020205	1.50	2.53E-62
Pde4b	ENSMUSG00000028525	1.50	3.98E-101
Prr13	ENSMUSG00000023048	1.50	5.46E-70
Gm15987	ENSMUSG00000085786	1.50	1.03E-91
Vegfa	ENSMUSG00000023951	1.50	1.16E-97
Hilpda	ENSMUSG00000043421	1.50	2.19E-42
Prdx5	ENSMUSG00000024953	1.50	1.78E-06
Dst	ENSMUSG00000026131	1.50	4.00E-45
Gm42418	ENSMUSG00000098178	1.49	1.84E-36
Tnfaip2	ENSMUSG00000021281	1.49	4.04E-94
Trps1	ENSMUSG00000038679	1.49	5.89E-32
Aqp9	ENSMUSG00000032204	1.48	2.24E-133
Syngr2	ENSMUSG00000048277	1.47	5.09E-23
Mpeg1	ENSMUSG00000046805	1.47	5.80E-70
Pabpc1	ENSMUSG00000022283	1.47	7.61E-118
Arl5c	ENSMUSG00000038352	1.46	1.27E-58
Ehd1	ENSMUSG00000024772	1.46	2.21E-107
Camk1d	ENSMUSG00000039145	1.46	5.55E-53
Vasp	ENSMUSG00000030403	1.46	2.14E-68
Rps27l	ENSMUSG00000036781	1.46	1.42E-26
Il6	ENSMUSG00000025746	1.45	3.72E-25
Pla2g4a	ENSMUSG00000056220	1.45	2.10E-43
Crem	ENSMUSG00000063889	1.45	8.41E-81
Slfn5	ENSMUSG00000054404	1.45	1.75E-06
Pgd	ENSMUSG00000028961	1.44	7.90E-74
Chst11	ENSMUSG00000034612	1.44	1.48E-96
Filip1l	ENSMUSG00000043336	1.44	1.56E-70

Slc2a6	ENSMUSG00000036067	1.44	2.75E-99
Smim3	ENSMUSG00000038059	1.44	2.10E-100
Mki67	ENSMUSG00000031004	1.43	8.02E-21
Lgals3	ENSMUSG00000050335	1.43	5.18E-61
Osgin2	ENSMUSG00000041153	1.43	2.46E-70
Cd53	ENSMUSG00000040747	1.43	1.88E-84
S100a8	ENSMUSG00000056054	1.42	0.000110893
Hdc	ENSMUSG00000027360	1.42	2.01E-52

Appendix Table S3. Transcriptional comparation of the antiviral genes in different tissues between water- and resveratrol-administrated mice at 24 hours post Zika virus infection (resveratrol *v.s.* water)*.

Gene Name	Gene ID	Log2 FC	p-value	q-value	Significant
**Footpad Skin**					
IFN alpha	ENSMUSG00000095498	0.00	1.00	1.00	No
IFN beta	ENSMUSG00000048806	1.27	0.99	1.00	No
IFN gamma	ENSMUSG00000055170	0.00	1.00	1.00	No
IFN lambda	ENSMUSG00000059128	0.00	1.00	1.00	No
IFITM3	ENSMUSG00000025492	0.76	0.03	0.21	No
STAT2	ENSMUSG00000040033	0.05	0.93	0.97	No
IRF3	ENSMUSG00000003184	0.58	0.15	0.40	No
IRF7	ENSMUSG00000025498	0.23	0.74	0.87	No
MX1	ENSMUSG00000000386	1.24	0.28	1.00	No
TLR3	ENSMUSG00000031639	-0.10	0.10	0.34	No
TLR7	ENSMUSG00000044583	-0.64	0.03	0.19	No
TLR8	ENSMUSG00000040522	-0.06	0.53	1.00	No
TLR9	ENSMUSG00000045322	-0.89	0.03	1.00	No
DDX58	ENSMUSG00000040296	0.04	0.15	0.41	No
DHX58	ENSMUSG00000017830	0.52	0.29	0.57	No
EIF2AK2	ENSMUSG00000024079	-0.26	0.01	0.15	No
MAVS	ENSMUSG00000037523	0.39	0.49	0.72	No
TRIM25	ENSMUSG00000000275	0.16	0.62	0.81	No
**Lymph Node**					

Gene Name	Gene ID	Log2 FC	p-value	q-value	Significant
IFN alpha	ENSMUSG00000095498	0.00	1.00	1.00	No
IFN beta	ENSMUSG00000048806	9.18	0.58	1.00	No
IFN gamma	ENSMUSG00000055170	-0.46	0.19	1.00	No
IFN lymda	ENSMUSG00000059128	0.00	1.00	1.00	No
IFITM3	ENSMUSG00000025492	0.23	0.98	1.00	No
STAT2	ENSMUSG00000040033	0.23	0.72	0.94	No
IRF3	ENSMUSG00000003184	-0.17	0.15	0.60	No
IRF7	ENSMUSG00000025498	-0.25	0.02	0.32	No
MX1	ENSMUSG00000000386	0.34	0.91	0.98	No
TLR3	ENSMUSG00000031639	0.14	0.87	0.98	No
TLR7	ENSMUSG00000044583	1.05	0.03	0.38	No
TLR8	ENSMUSG00000040522	0.68	0.45	1.00	No
TLR9	ENSMUSG00000045322	0.16	0.83	0.96	No
DDX58	ENSMUSG00000040296	0.17	0.56	0.87	No
DHX58	ENSMUSG00000017830	-0.08	0.68	0.93	No
EIF2AK2	ENSMUSG00000024079	0.25	0.75	0.94	No
MAVS	ENSMUSG00000037523	0.01	0.75	0.95	No
TRIM25	ENSMUSG00000000275	0.30	0.65	0.91	No
**Spleen**					
**Gene Name**	**Gene ID**	**Log2 FC**	**p-value**	**q-value**	**Significant**
IFN alpha	ENSMUSG00000095498	-8.88	0.71	1.00	No
IFN beta	ENSMUSG00000048806	0.52	0.74	1.00	No

IFN gamma	ENSMUSG00000055170	-0.51	0.43	1.00	No
IFN lymda	ENSMUSG00000059128	0.00	1.00	1.00	No
IFITM3	ENSMUSG00000025492	-0.55	0.04	1.00	No
STAT2	ENSMUSG00000040033	-0.23	0.26	1.00	No
IRF3	ENSMUSG00000003184	-0.16	0.78	1.00	No
IRF7	ENSMUSG00000025498	-0.42	0.05	1.00	No
MX1	ENSMUSG00000000386	0.00	0.57	1.00	No
TLR3	ENSMUSG00000031639	0.08	0.63	1.00	No
TLR7	ENSMUSG00000044583	-0.08	0.84	1.00	No
TLR8	ENSMUSG00000040522	-0.49	0.37	1.00	No
TLR9	ENSMUSG00000045322	-0.16	0.72	1.00	No
DDX58	ENSMUSG00000040296	-0.02	0.82	1.00	No
DHX58	ENSMUSG00000017830	-0.28	0.38	1.00	No
EIF2AK2	ENSMUSG00000024079	-0.13	0.89	1.00	No
MAVS	ENSMUSG00000037523	-0.13	0.74	1.00	No
TRIM25	ENSMUSG00000000275	-0.22	0.37	1.00	No

* The original RNA sequencing data is available at NCBI SRA with accession: PRJNA1045313.

Appendix Table S4. Primers used in the study.

Target Gene	Sense	Anti-sense
Primers for qPCR		
mouse *Cxcl1*	GCTCCCTTGGTTCAGAAAATTG	TCACCAGACAGGTGCCATCA
mouse *Cxcl2*	TCATAGCCACTCTCAAGGG	TTGGTTCTTCCGTTGAGGG
mouse *Cxcl3*	CAGCCACACTCCAGCCTA	CACAACAGCCCCTGTAGC
mouse *Gapdh*	TCTTGGGCTACACTGAGGAC	CATACCAGGAAATGAGCTTGA
A. aegypti AaNRP	TCATAACCTTTCCGAAGAGG	GCACCAAACAAGATTCTGC
A. aegypti β-actin	GAACACCCAGTCCTGCTGACA	TGCGTCATCTTCTCACGGTTAG
Primers for dsRNA synthesis		
GFP dsRNA	TAATACGACTCACTATAGGGGTGAGCAAGGGCGAGGAG	TAATACGACTCACTATAGGGCATGATATAGACGTTGTGGCTGTT
AaNRP dsRNA	TAATACGACTCACTATAGGGGTGTGCCGACGAAAGTTT	TAATACGACTCACTATAGGGCTGGTCCTTGCTTACTGGTT
