# Supplementary figures and images for: A mosquito salivary protein-driven influx of myeloid cells facilitates flavivirus transmission
Source: EMBO J. 2024 Feb 20;43(9):2. doi: 10.1038/s44318-024-00056-x (PMC11066113; doi:10.1038/s44318-024-00056-x)

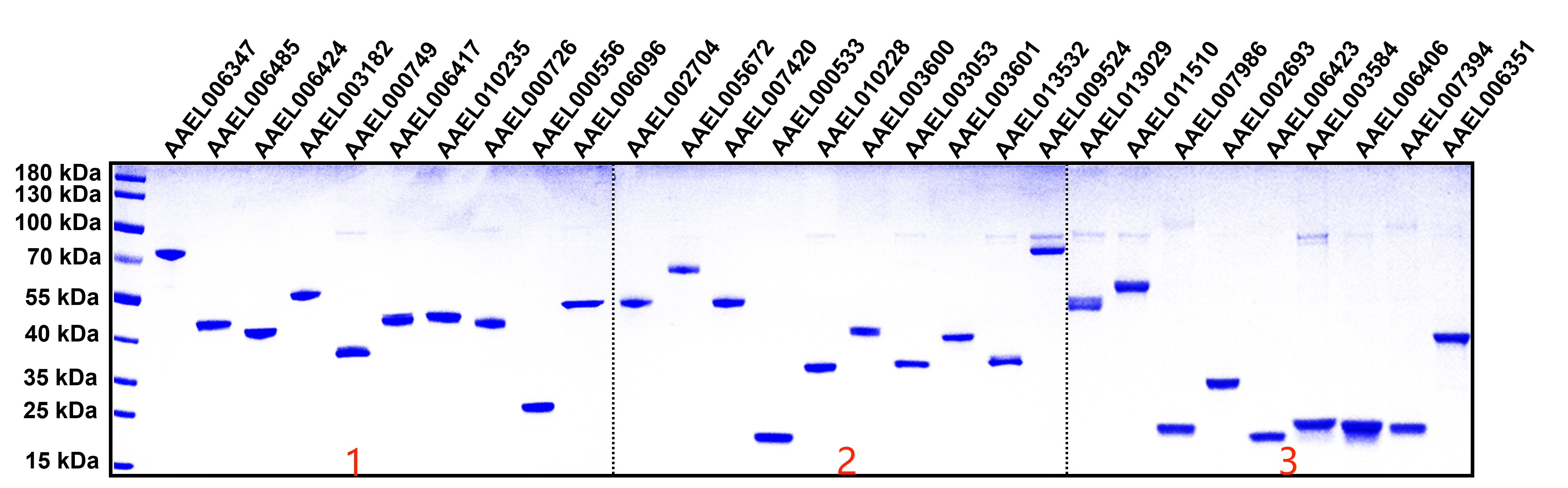

Supplement: Supplementary file 2 — Source Data Fig. 1 [file 44318_2024_56_MOESM2_ESM.zip › Fig 1/Fig 1A/Combined SDS-PAGE images with splice sites indicated by vertical dashed lines.png]

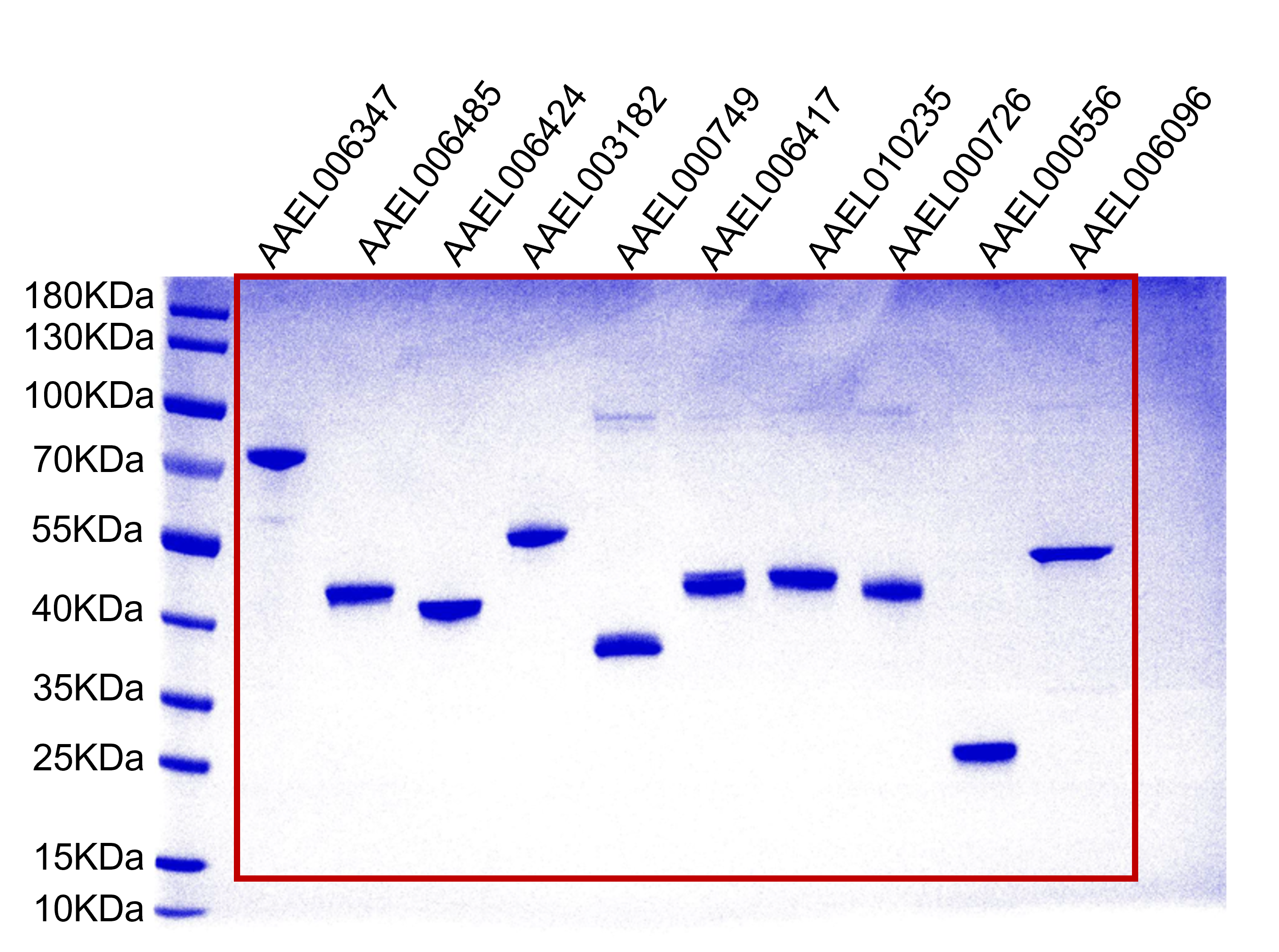

Supplement: Supplementary file 2 — Source Data Fig. 1 [file 44318_2024_56_MOESM2_ESM.zip › Fig 1/Fig 1A/SDS-PAGE-1.tif]

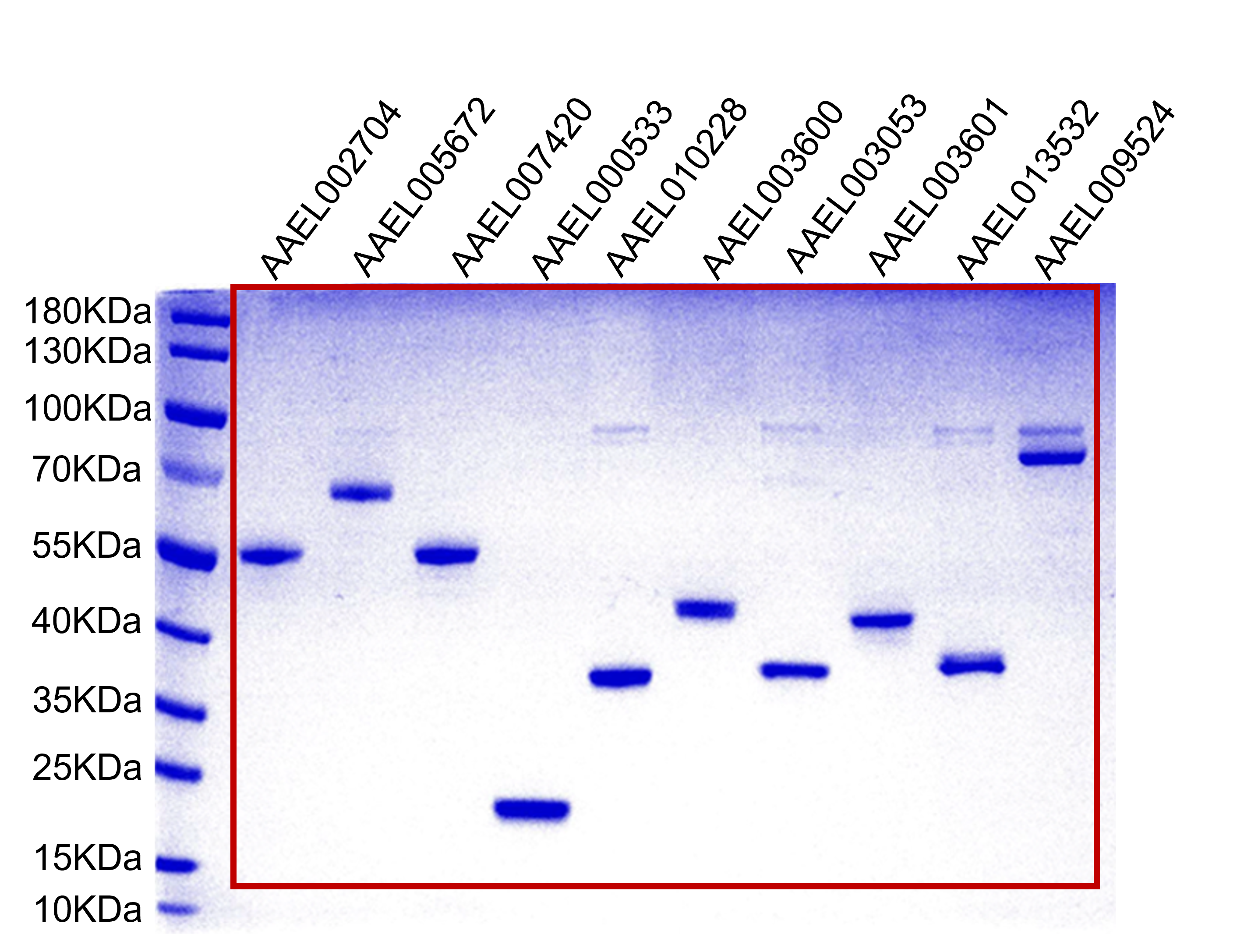

Supplement: Supplementary file 2 — Source Data Fig. 1 [file 44318_2024_56_MOESM2_ESM.zip › Fig 1/Fig 1A/SDS-PAGE-2.tif]

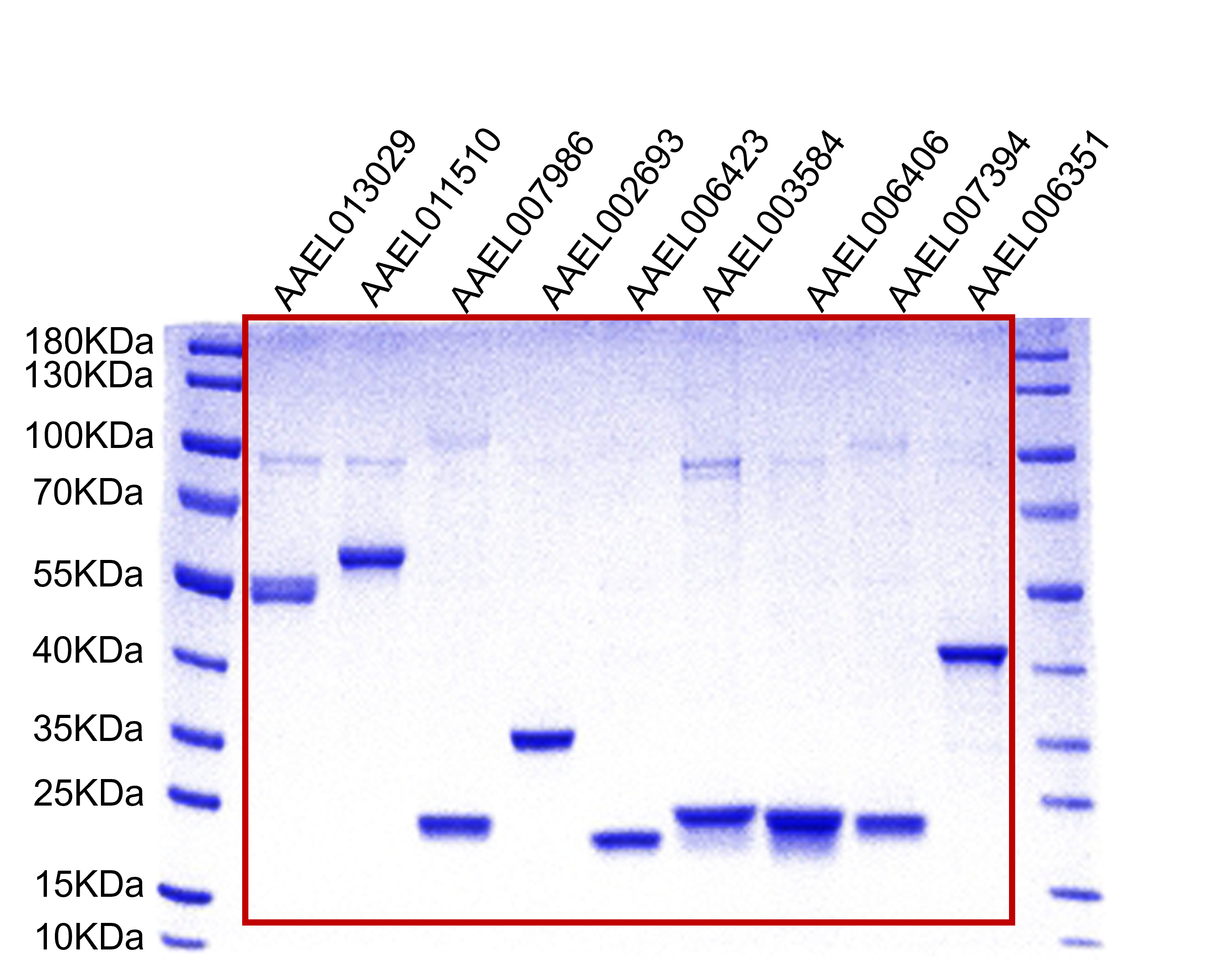

Supplement: Supplementary file 2 — Source Data Fig. 1 [file 44318_2024_56_MOESM2_ESM.zip › Fig 1/Fig 1A/SDS-PAGE-3.tif]

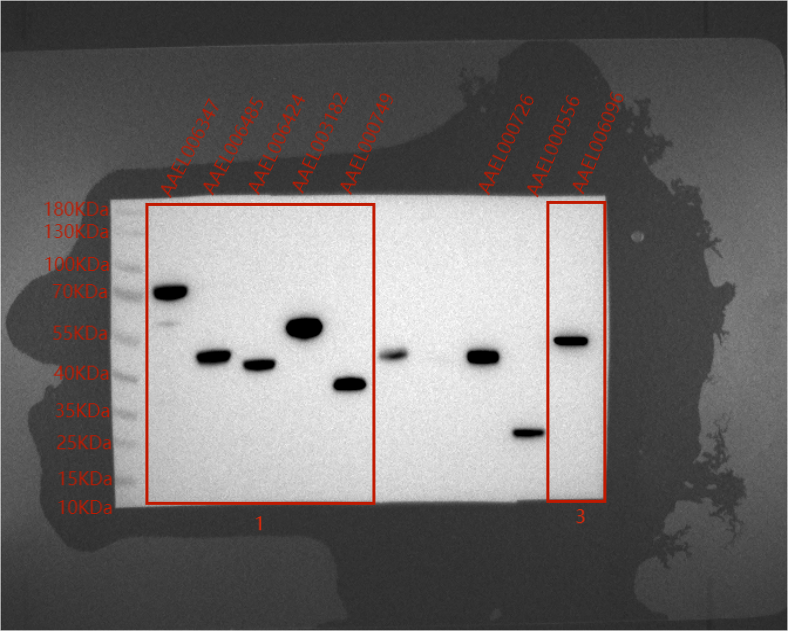

Supplement: Supplementary file 2 — Source Data Fig. 1 [file 44318_2024_56_MOESM2_ESM.zip › Fig 1/Fig 1B/1+3.png]

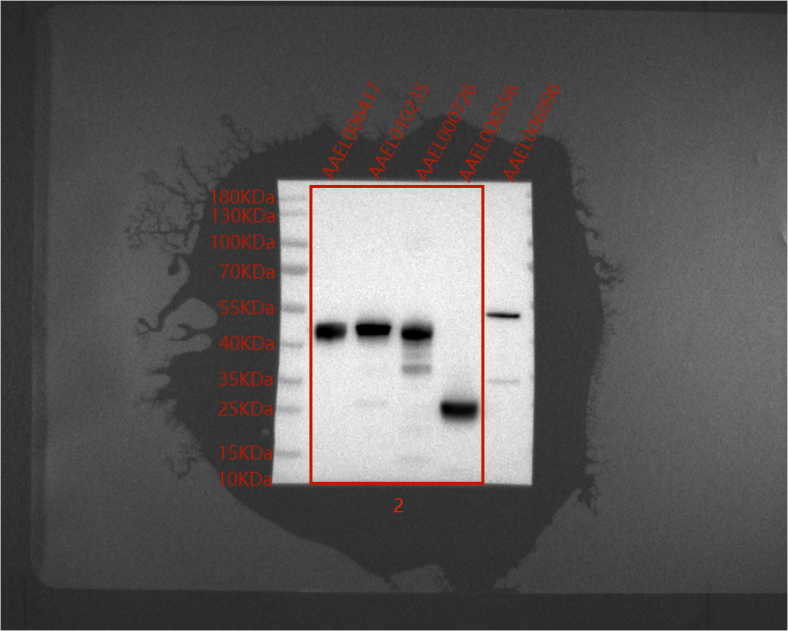

Supplement: Supplementary file 2 — Source Data Fig. 1 [file 44318_2024_56_MOESM2_ESM.zip › Fig 1/Fig 1B/2.png]

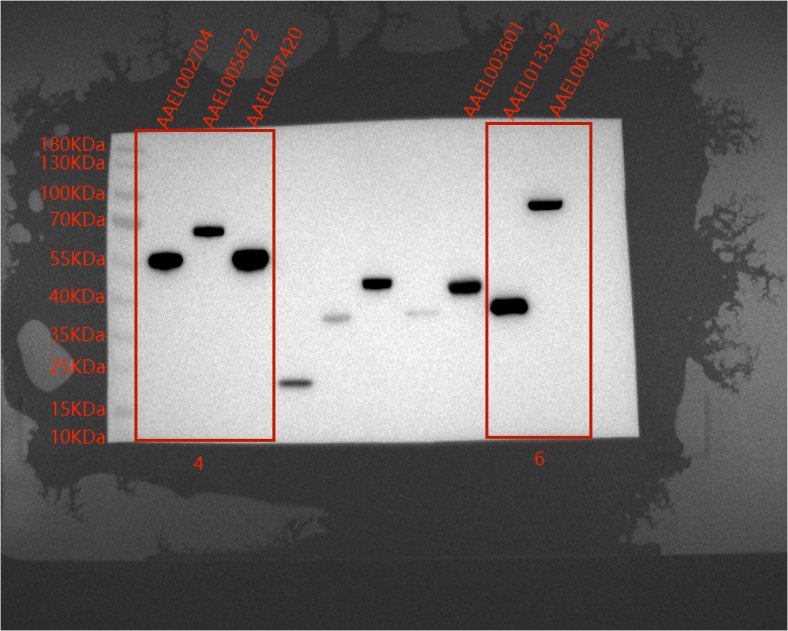

Supplement: Supplementary file 2 — Source Data Fig. 1 [file 44318_2024_56_MOESM2_ESM.zip › Fig 1/Fig 1B/4+6.png]

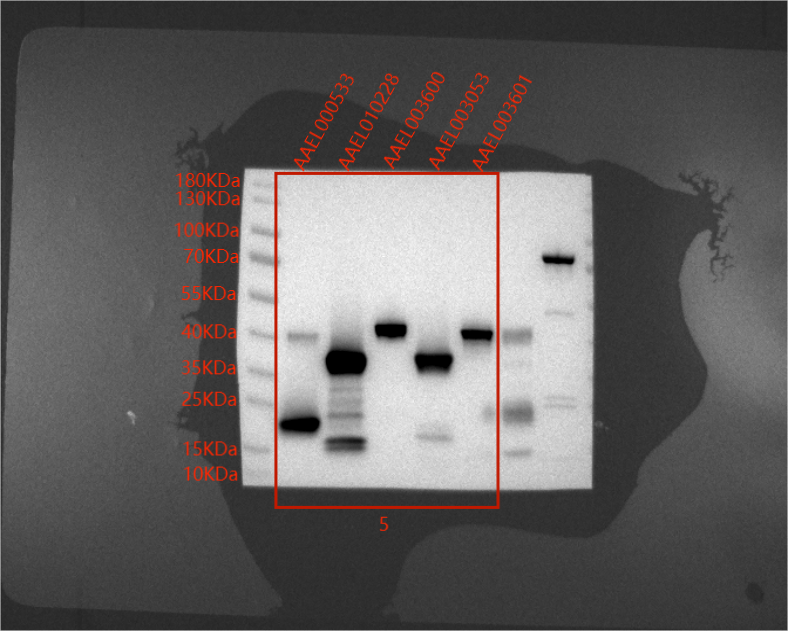

Supplement: Supplementary file 2 — Source Data Fig. 1 [file 44318_2024_56_MOESM2_ESM.zip › Fig 1/Fig 1B/5.png]

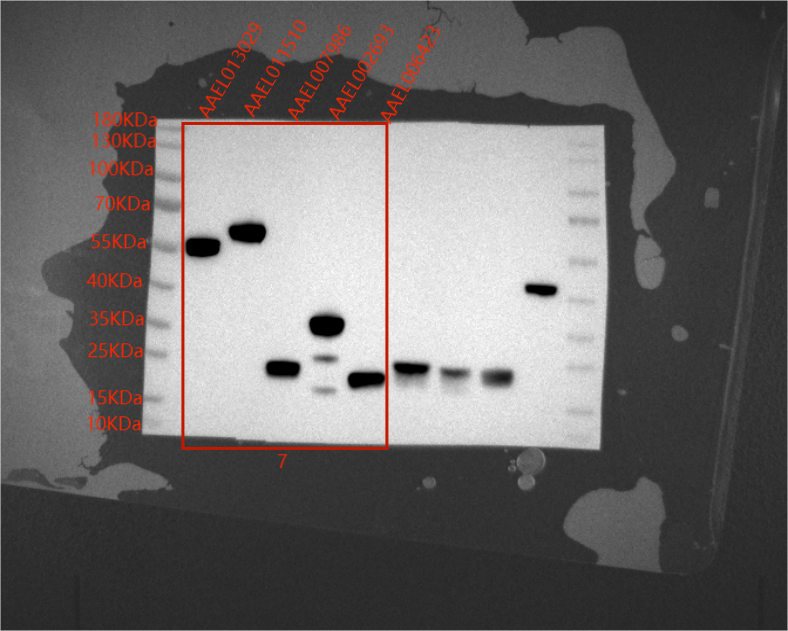

Supplement: Supplementary file 2 — Source Data Fig. 1 [file 44318_2024_56_MOESM2_ESM.zip › Fig 1/Fig 1B/7.png]

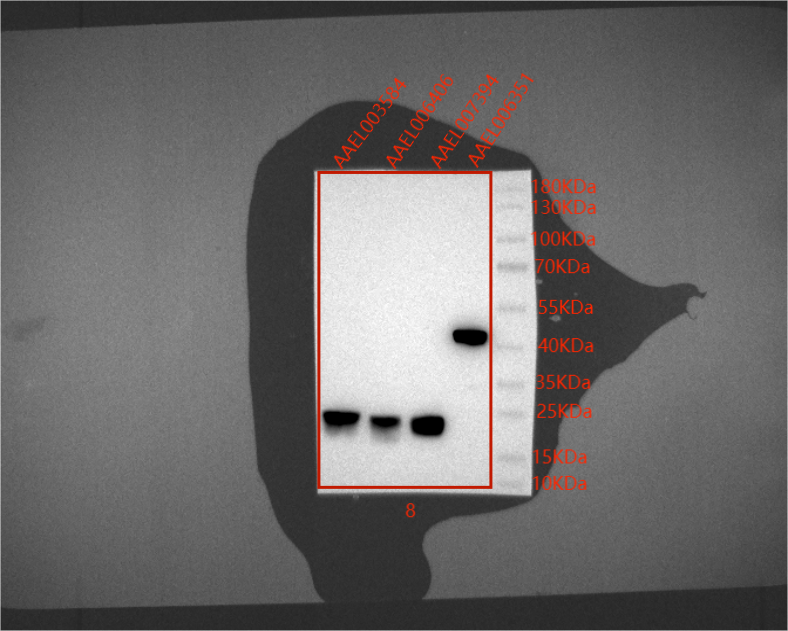

Supplement: Supplementary file 2 — Source Data Fig. 1 [file 44318_2024_56_MOESM2_ESM.zip › Fig 1/Fig 1B/8.png]

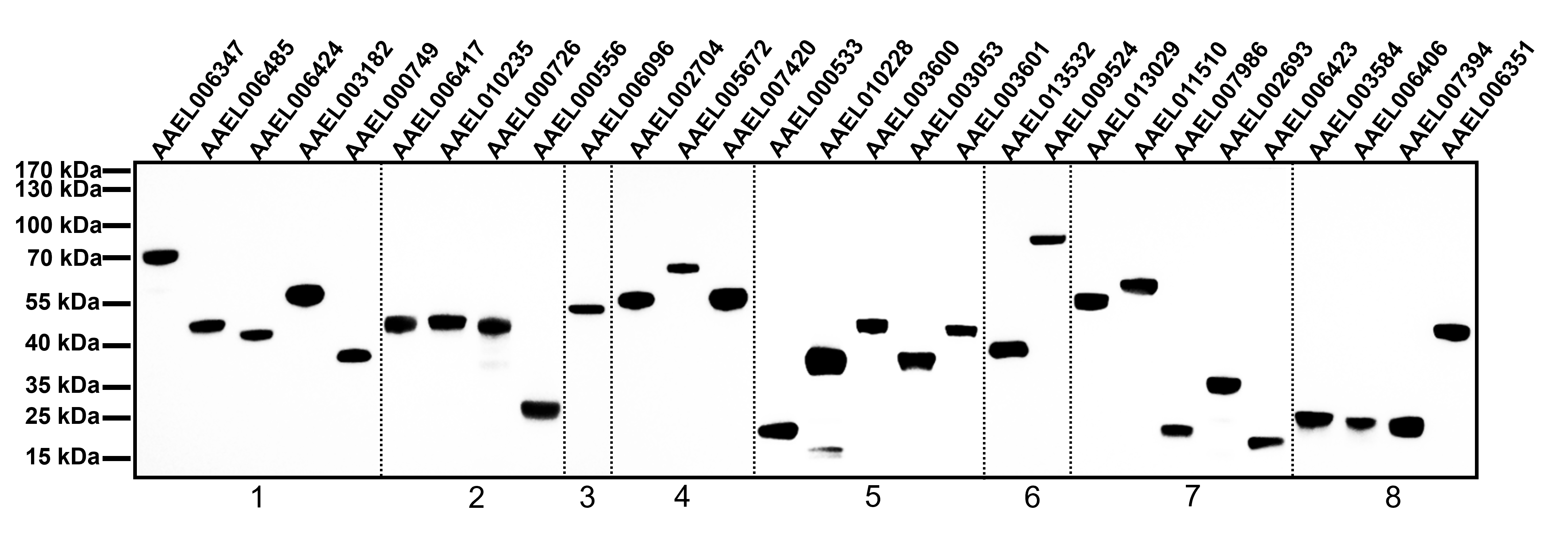

Supplement: Supplementary file 2 — Source Data Fig. 1 [file 44318_2024_56_MOESM2_ESM.zip › Fig 1/Fig 1B/Combined WB images with splice sites indicated by vertical dashed lines.tif]

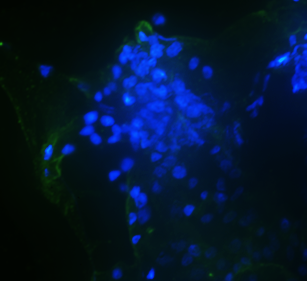

Supplement: Supplementary file 2 — Source Data Fig. 1 [file 44318_2024_56_MOESM2_ESM.zip › Fig 1/Fig 1J/AaNRP-dsRNA SG image.tif]

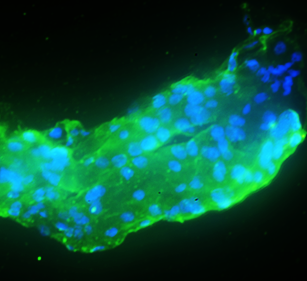

Supplement: Supplementary file 2 — Source Data Fig. 1 [file 44318_2024_56_MOESM2_ESM.zip › Fig 1/Fig 1J/GFP-dsRNA SG image.tif]

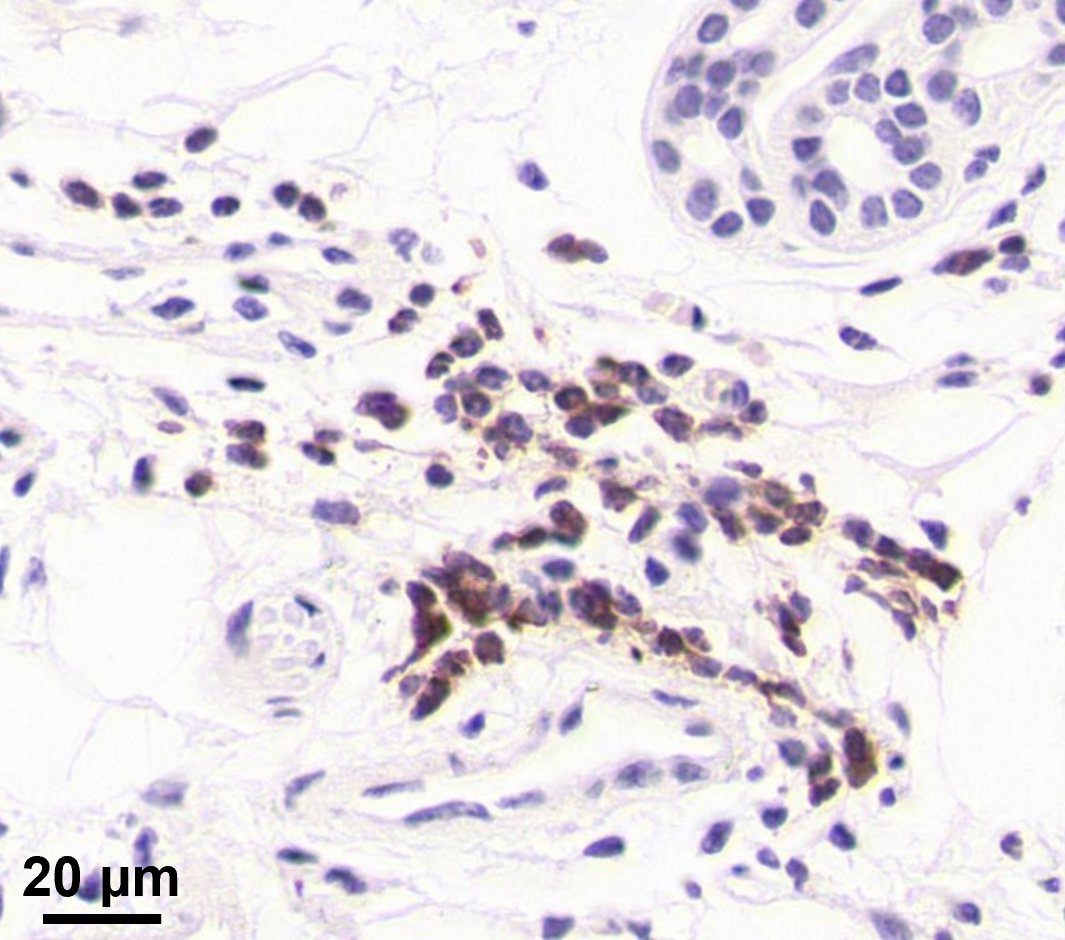

Supplement: Supplementary file 3 — Source Data Fig. 2 [file 44318_2024_56_MOESM3_ESM.zip › Fig 2/Fig 2F/Iso IgG+Mosquito Bite.tif]

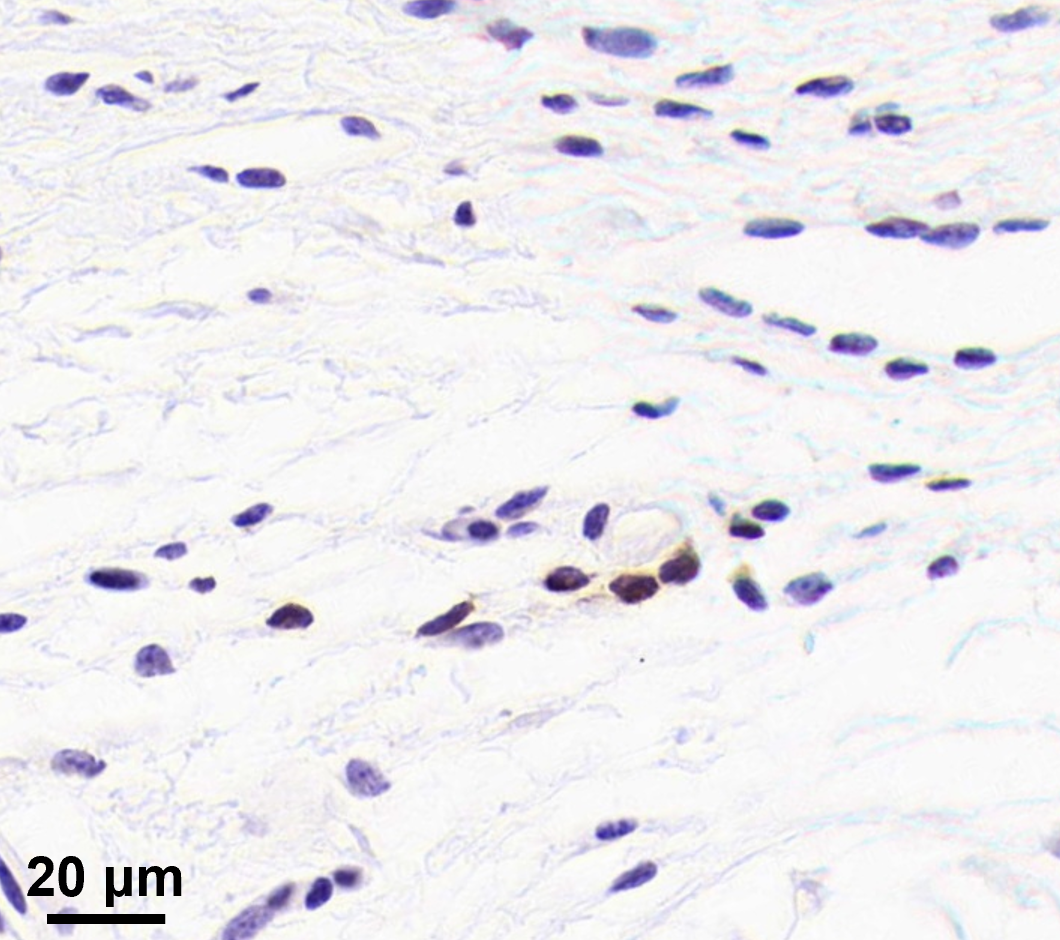

Supplement: Supplementary file 3 — Source Data Fig. 2 [file 44318_2024_56_MOESM3_ESM.zip › Fig 2/Fig 2F/Resting.tif]

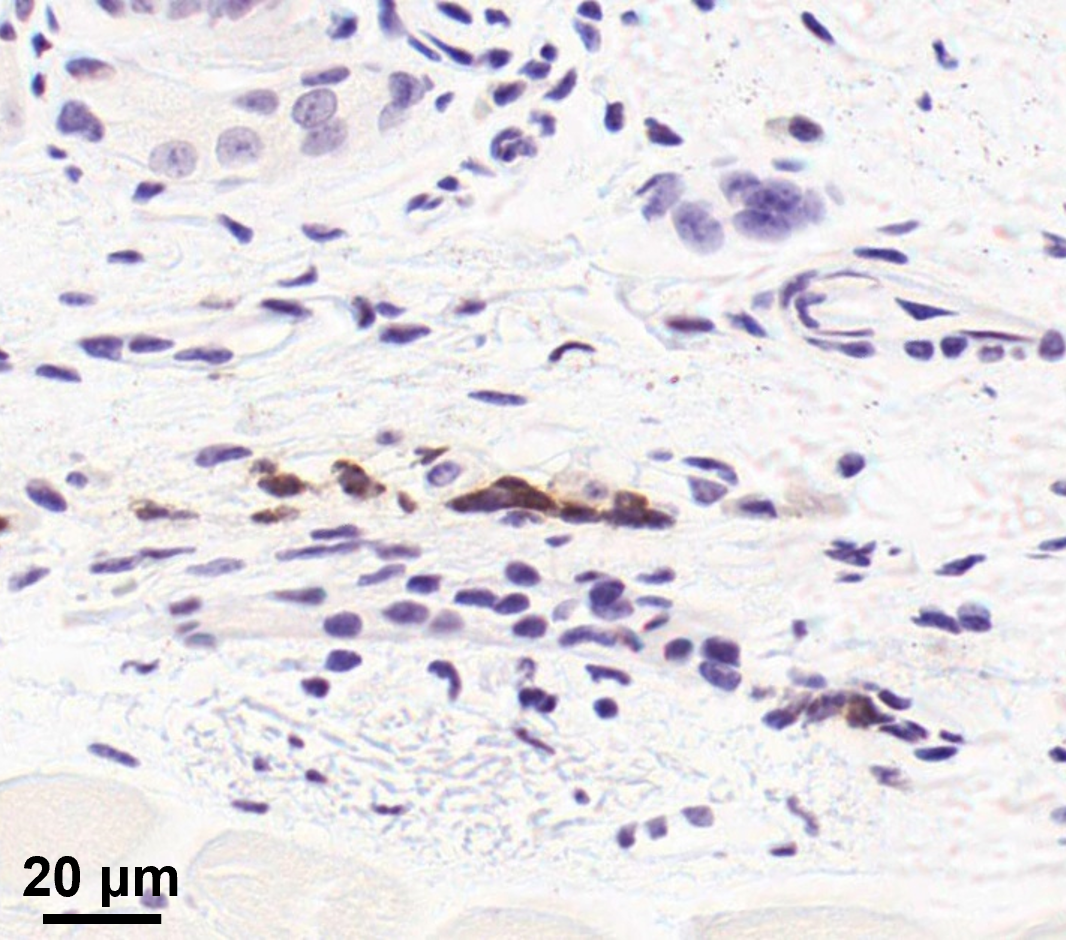

Supplement: Supplementary file 3 — Source Data Fig. 2 [file 44318_2024_56_MOESM3_ESM.zip › Fig 2/Fig 2F/Anti-F4 80 mAb+Mosquito Bite.tif]

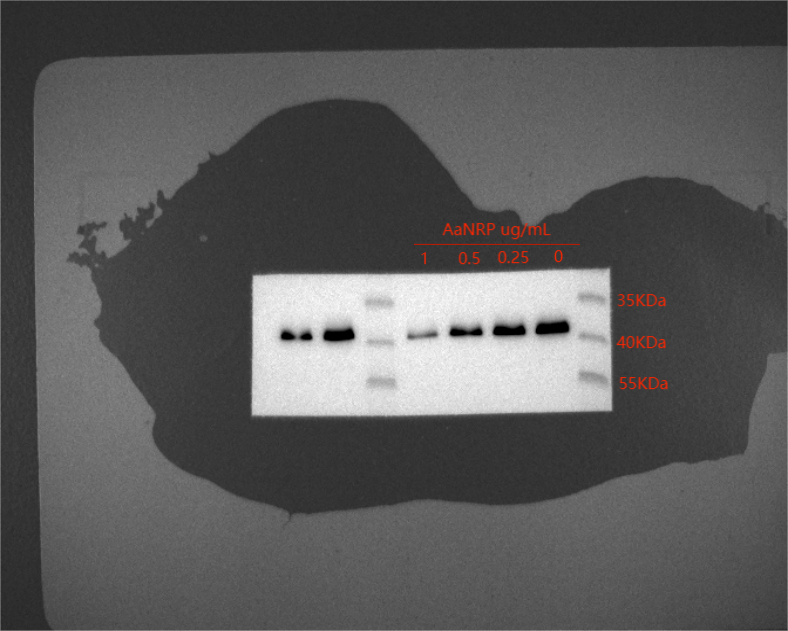

Supplement: Supplementary file 4 — Source Data Fig. 3 [file 44318_2024_56_MOESM4_ESM.zip › Fig 3/Fig 3C/IkB.png]

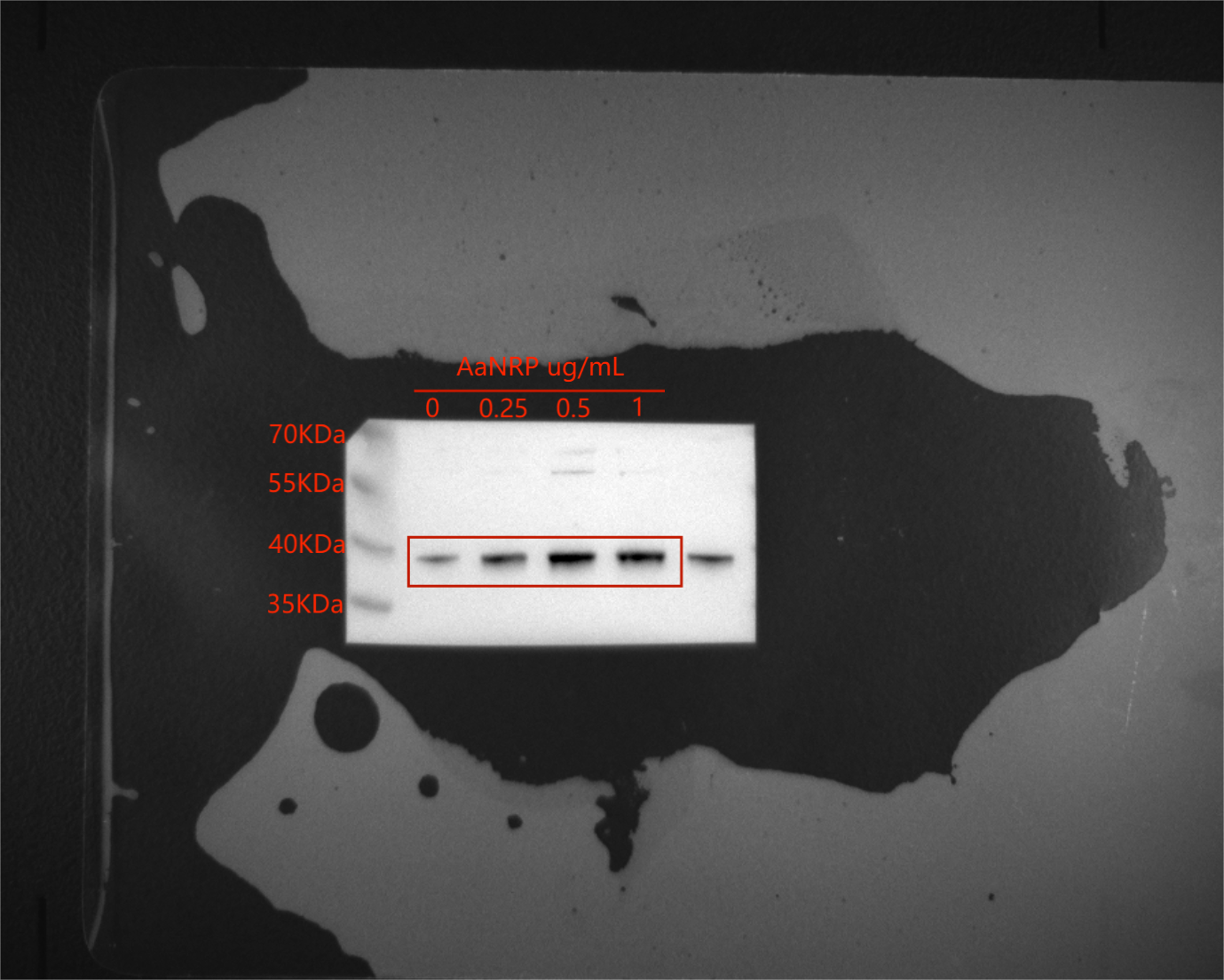

Supplement: Supplementary file 4 — Source Data Fig. 3 [file 44318_2024_56_MOESM4_ESM.zip › Fig 3/Fig 3C/p-IkB.png]

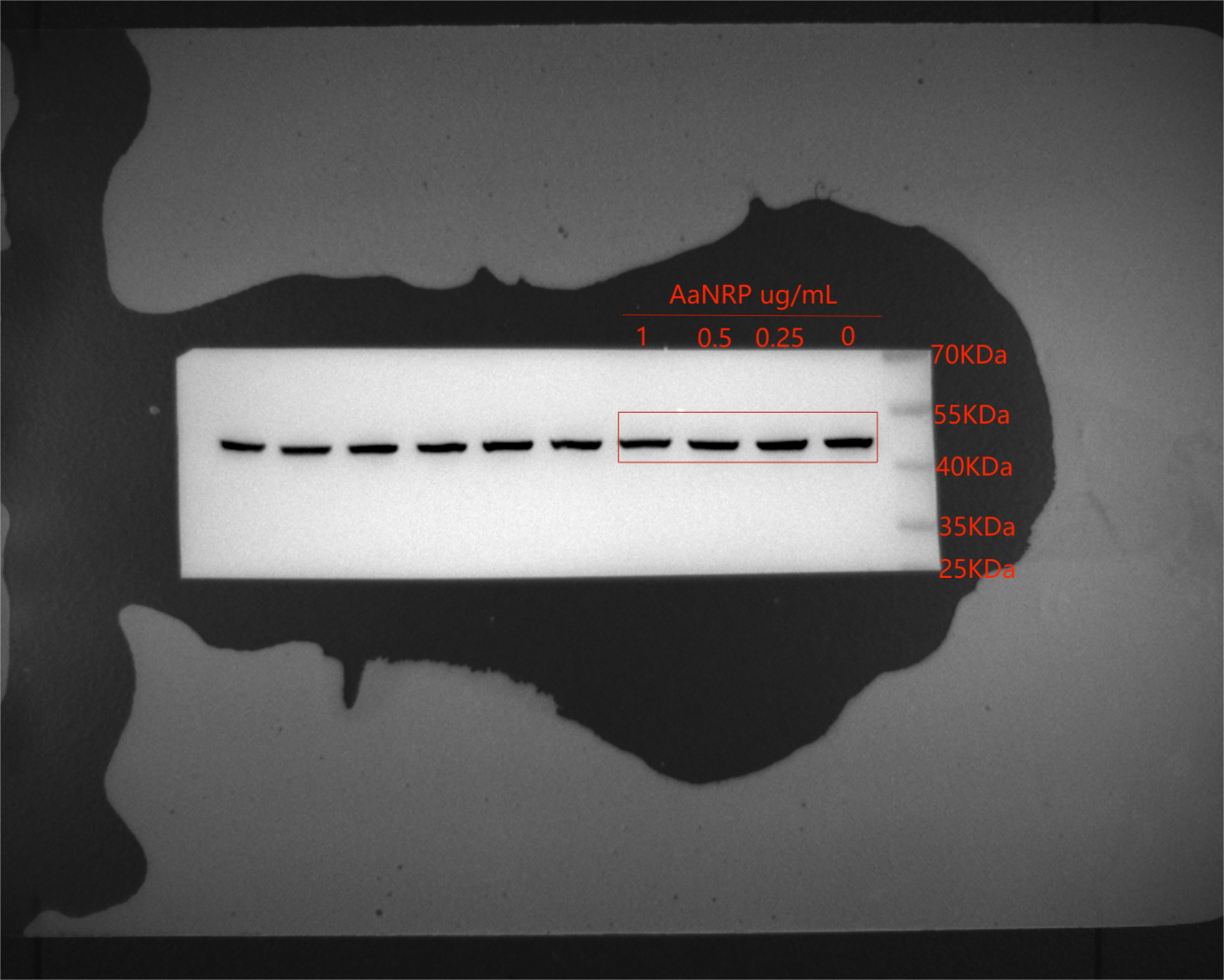

Supplement: Supplementary file 4 — Source Data Fig. 3 [file 44318_2024_56_MOESM4_ESM.zip › Fig 3/Fig 3C/┬aA╠ä-actin.png]

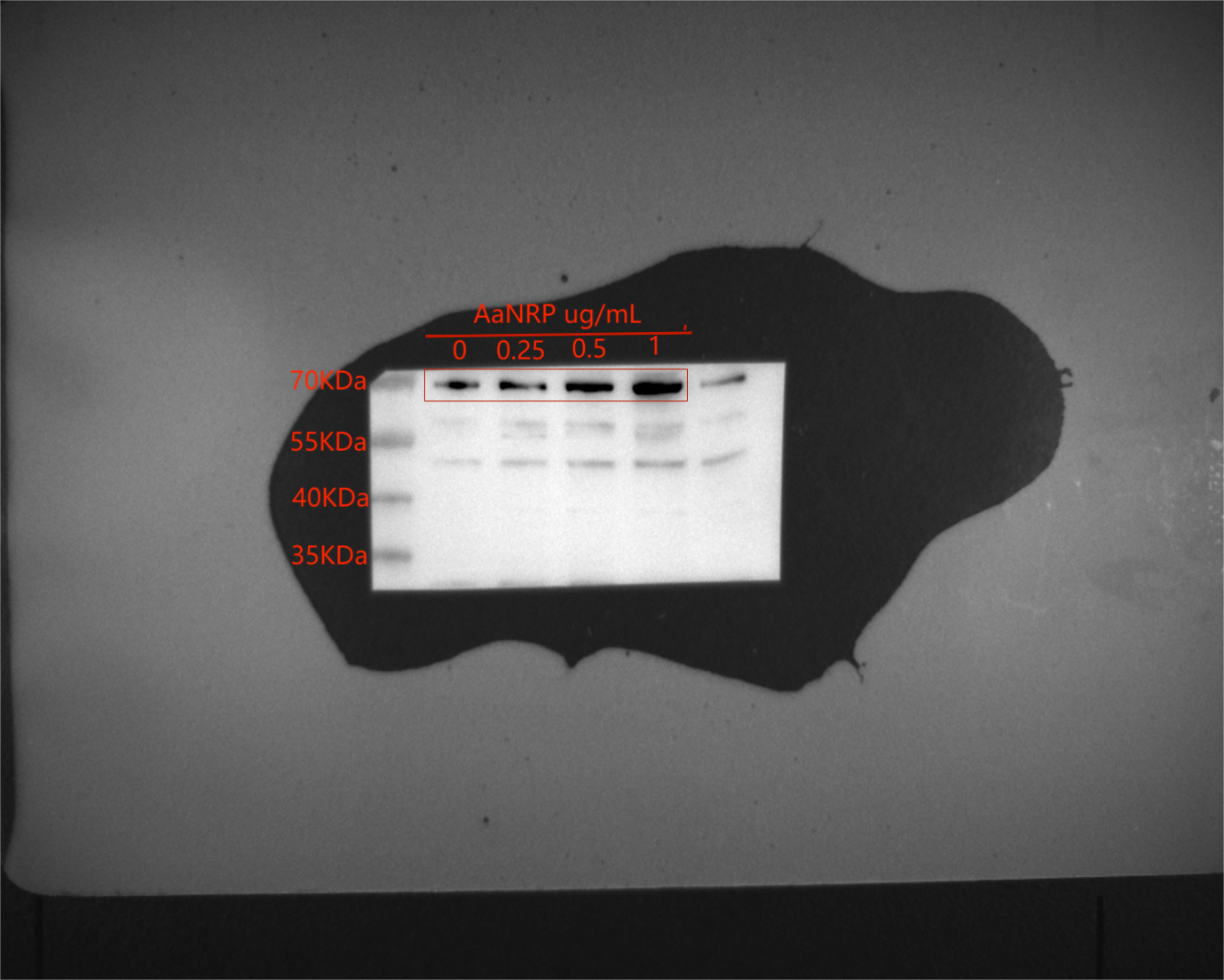

Supplement: Supplementary file 4 — Source Data Fig. 3 [file 44318_2024_56_MOESM4_ESM.zip › Fig 3/Fig 3C/p-p65.png]

## Slide 1
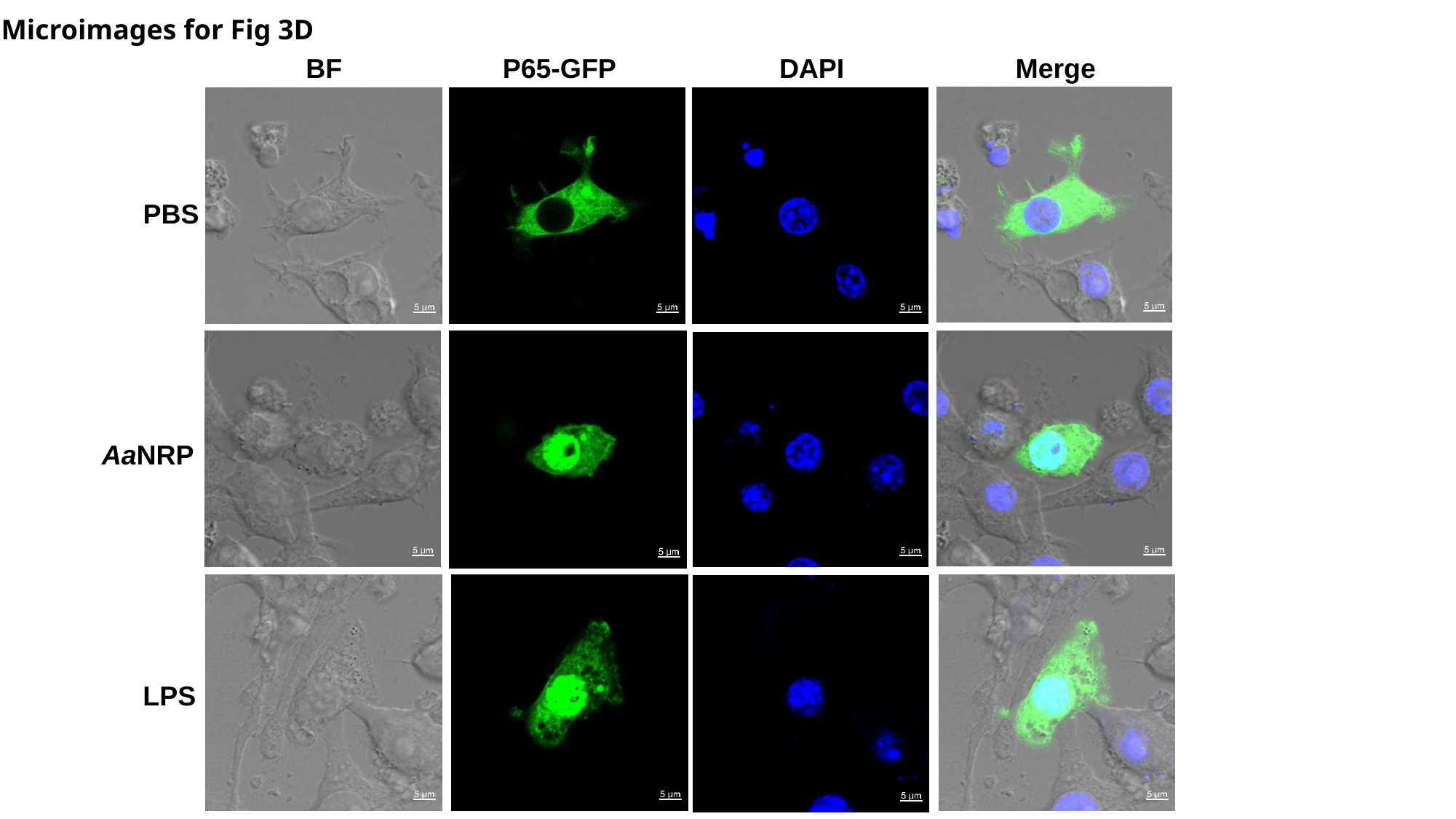

Microimages for Fig 3D
BF
P65-GFP
DAPI
Merge
PBS
AaNRP
LPS

Supplement: Supplementary file 4 — Source Data Fig. 3 [file 44318_2024_56_MOESM4_ESM.zip › Fig 3/Fig 3D/microimages for Fig 3D.pptx]

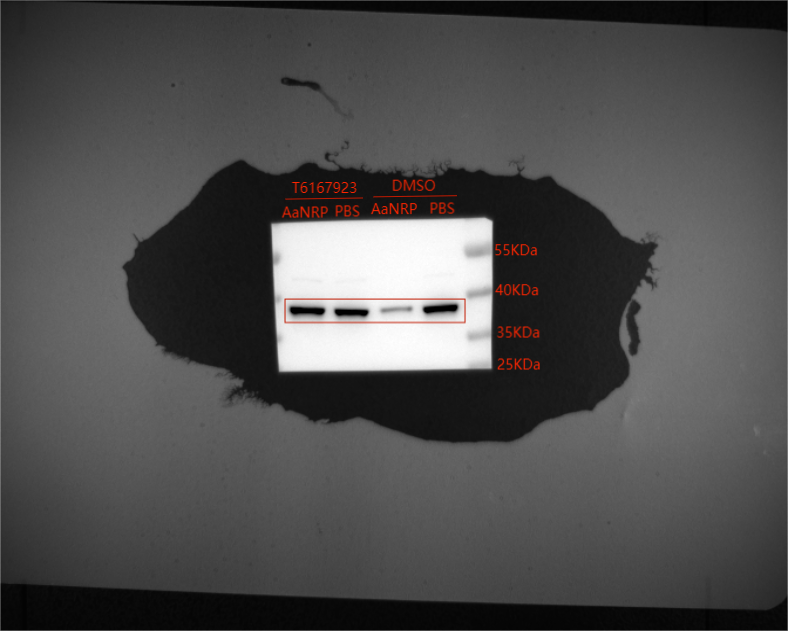

Supplement: Supplementary file 4 — Source Data Fig. 3 [file 44318_2024_56_MOESM4_ESM.zip › Fig 3/Fig 3E/Fig 3E ii primary macrophage/IkB.png]

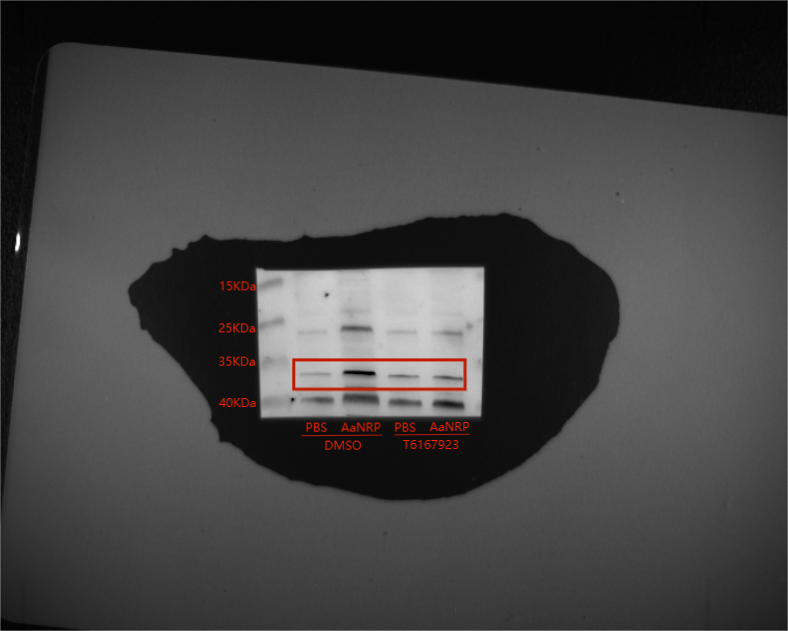

Supplement: Supplementary file 4 — Source Data Fig. 3 [file 44318_2024_56_MOESM4_ESM.zip › Fig 3/Fig 3E/Fig 3E ii primary macrophage/p-IkB.png]

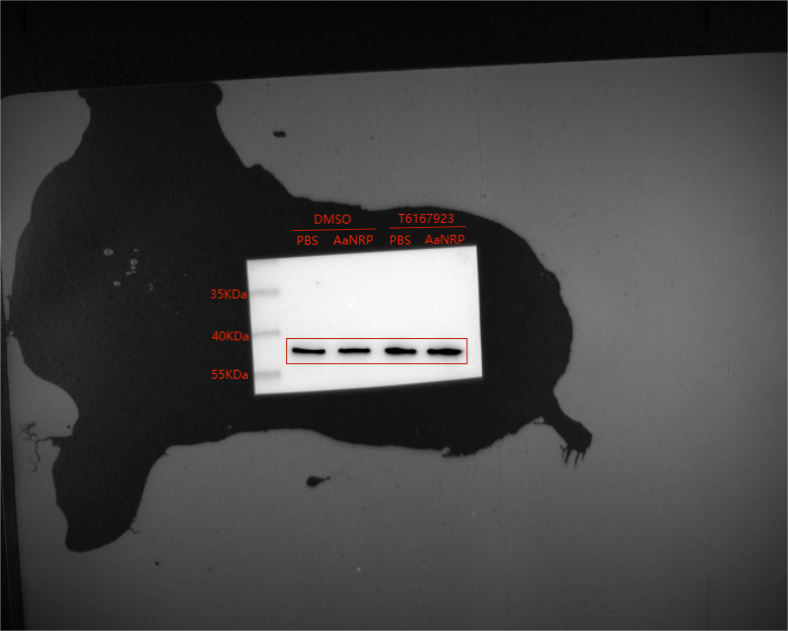

Supplement: Supplementary file 4 — Source Data Fig. 3 [file 44318_2024_56_MOESM4_ESM.zip › Fig 3/Fig 3E/Fig 3E ii primary macrophage/┬aA╠ä-actin.png]

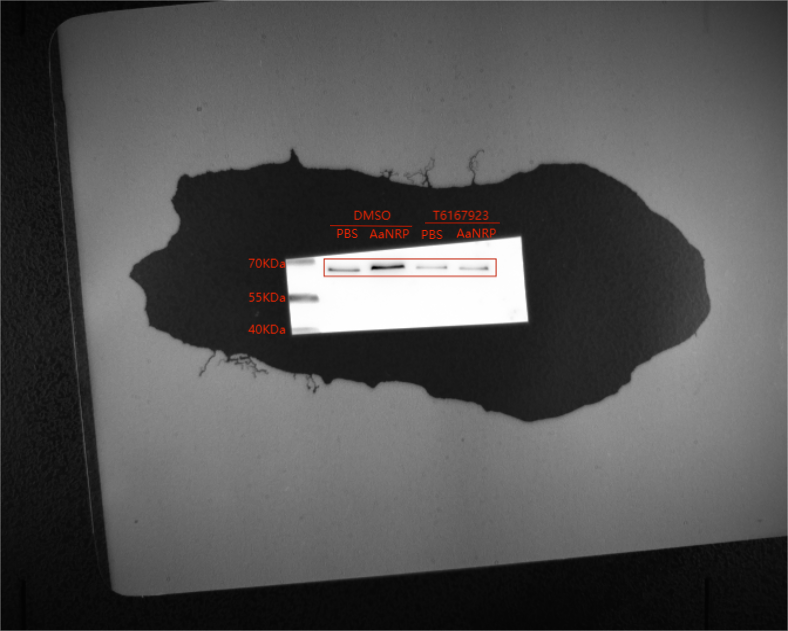

Supplement: Supplementary file 4 — Source Data Fig. 3 [file 44318_2024_56_MOESM4_ESM.zip › Fig 3/Fig 3E/Fig 3E ii primary macrophage/p-p65.png]

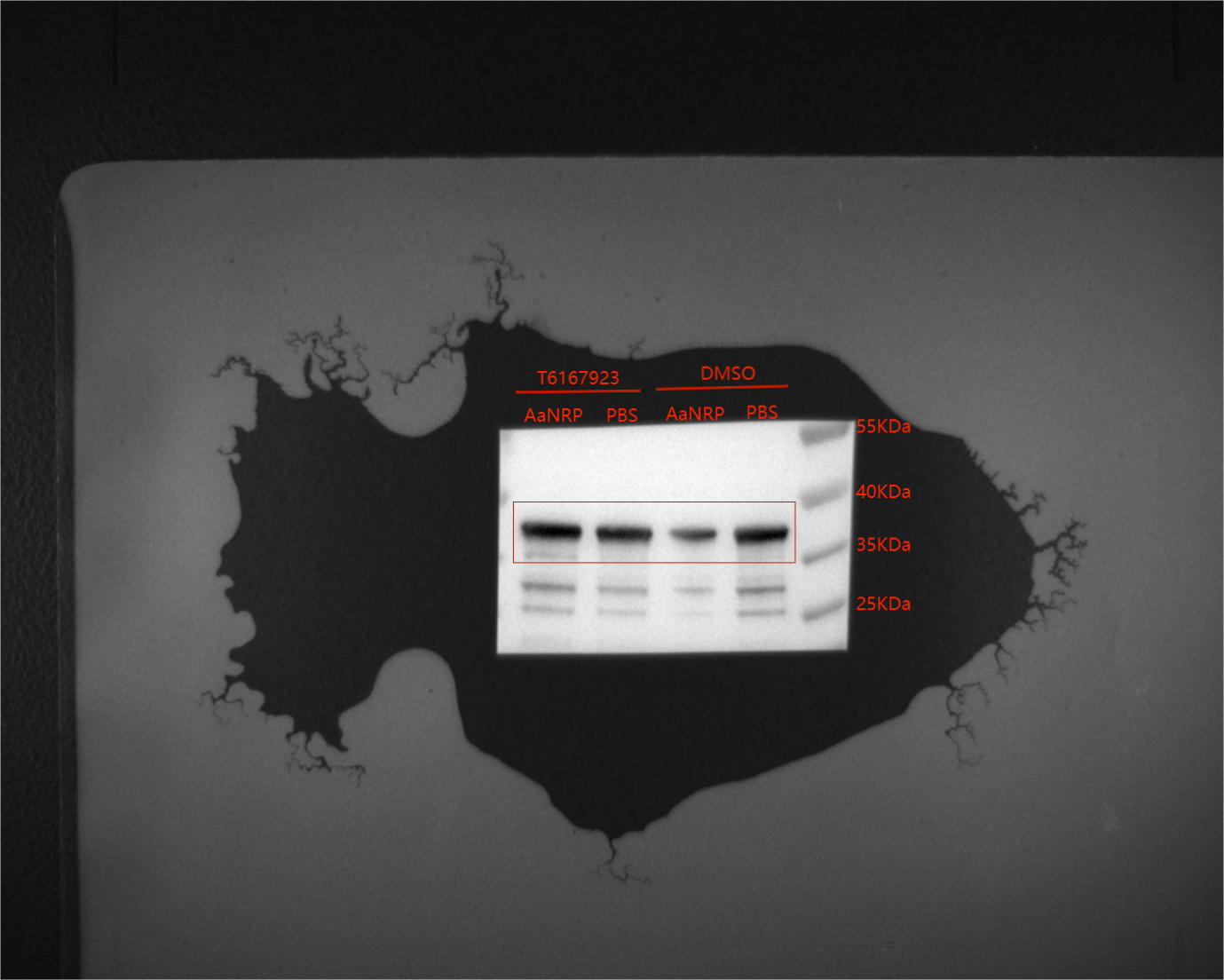

Supplement: Supplementary file 4 — Source Data Fig. 3 [file 44318_2024_56_MOESM4_ESM.zip › Fig 3/Fig 3E/Fig 3E i RAW264.7/IkB.png]

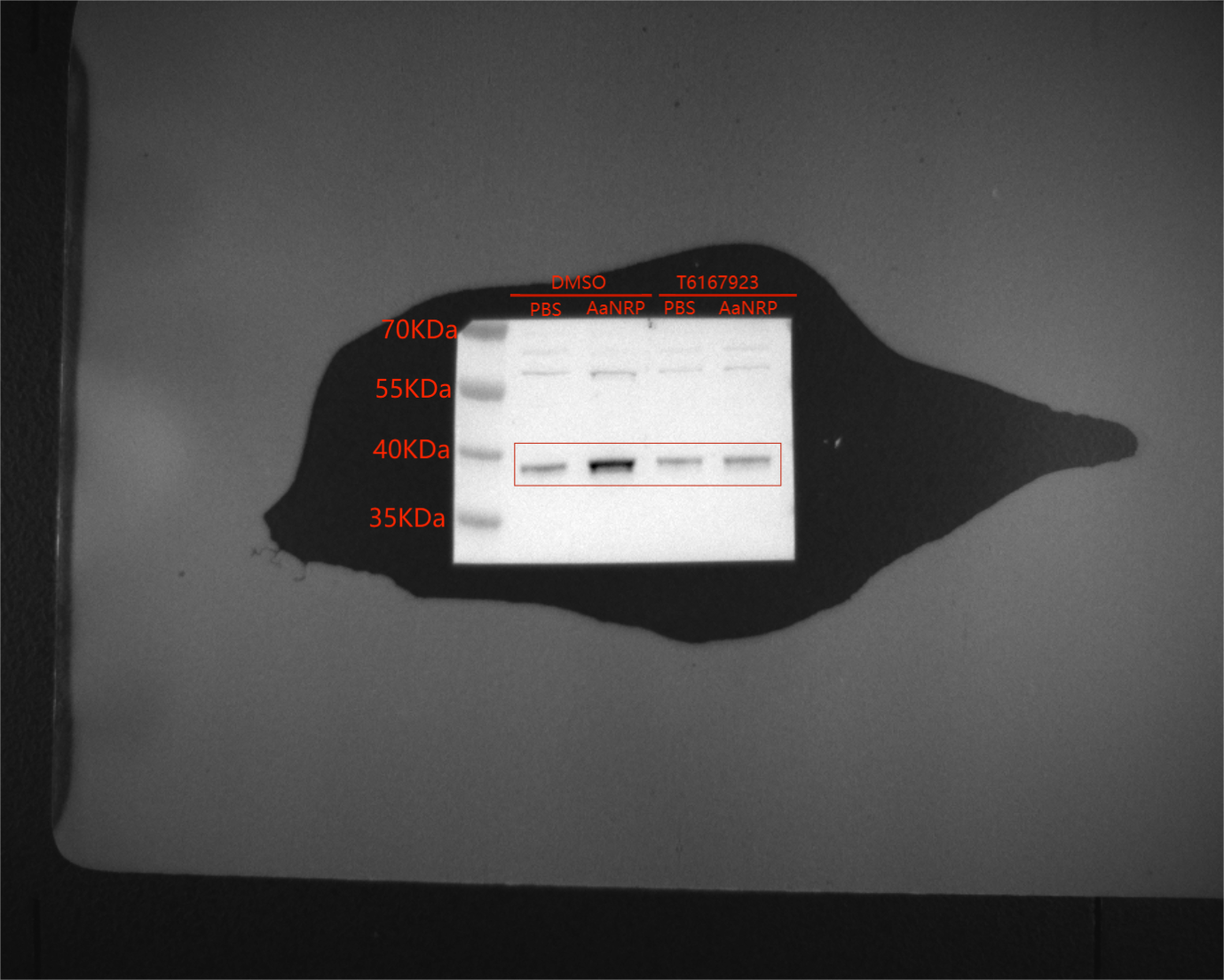

Supplement: Supplementary file 4 — Source Data Fig. 3 [file 44318_2024_56_MOESM4_ESM.zip › Fig 3/Fig 3E/Fig 3E i RAW264.7/p-IkB.png]

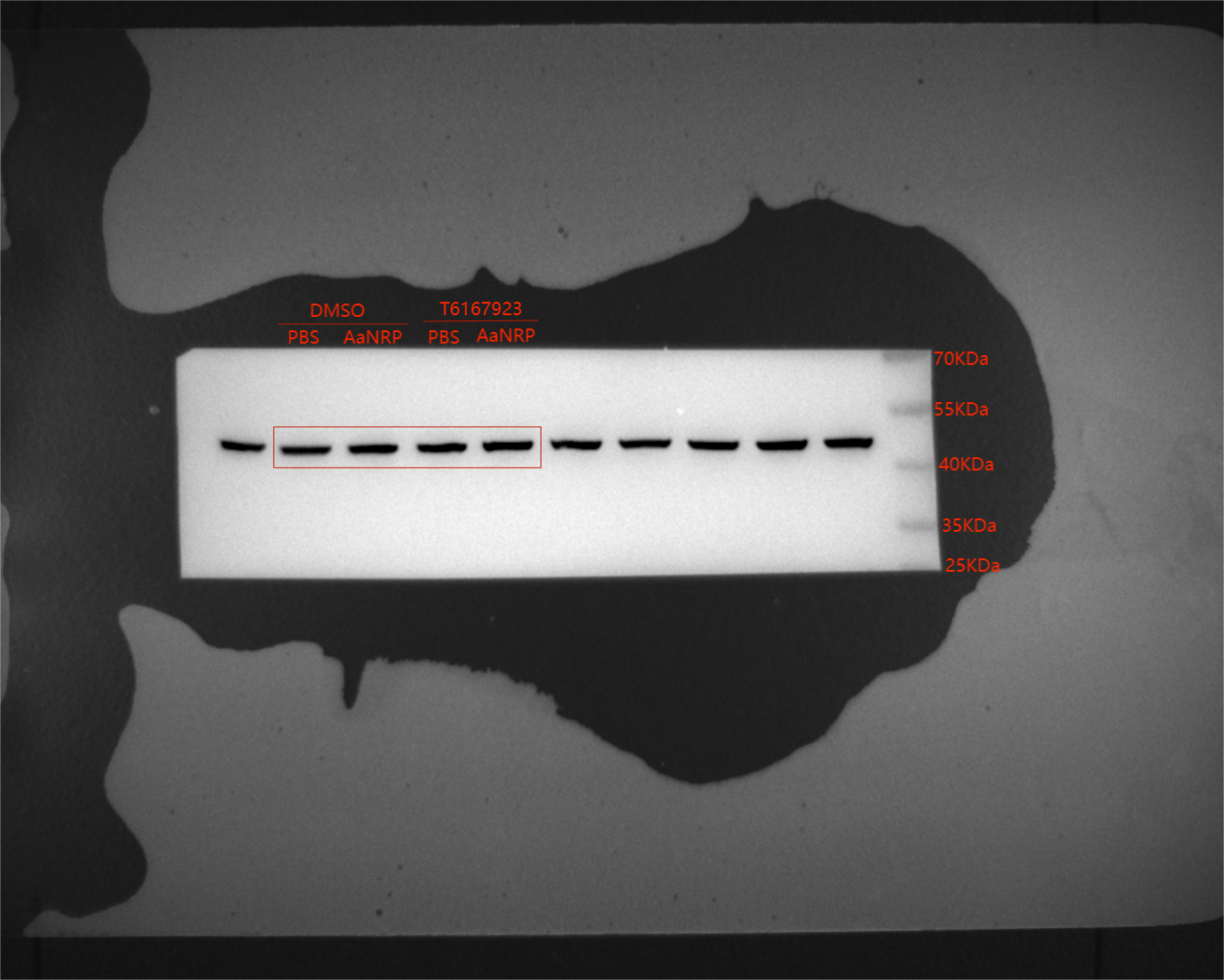

Supplement: Supplementary file 4 — Source Data Fig. 3 [file 44318_2024_56_MOESM4_ESM.zip › Fig 3/Fig 3E/Fig 3E i RAW264.7/┬aA╠ä-actin.png]

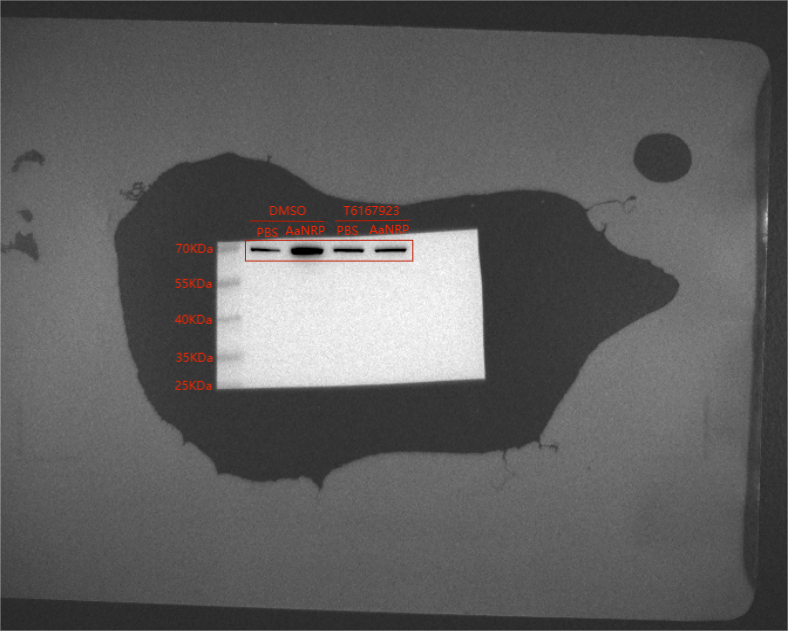

Supplement: Supplementary file 4 — Source Data Fig. 3 [file 44318_2024_56_MOESM4_ESM.zip › Fig 3/Fig 3E/Fig 3E i RAW264.7/p-p65.png]

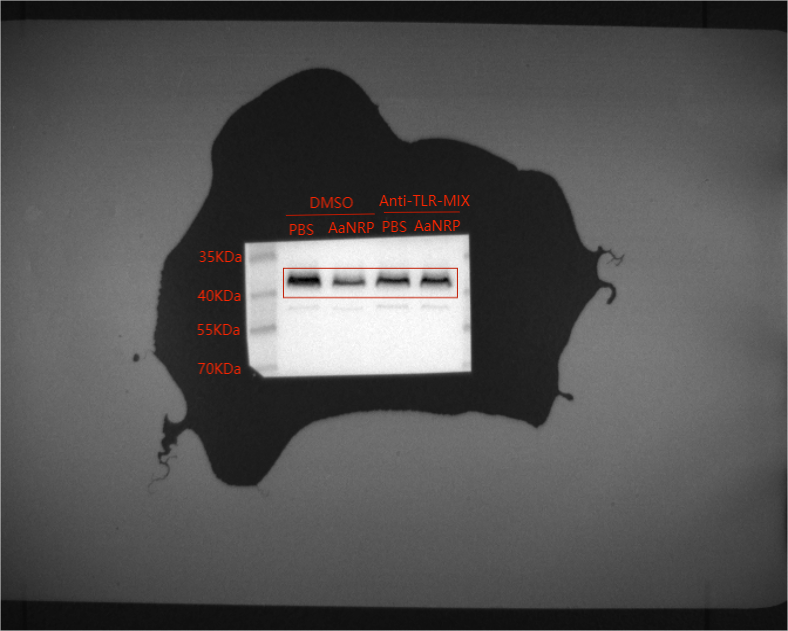

Supplement: Supplementary file 5 — Source Data Fig. 4 [file 44318_2024_56_MOESM5_ESM.zip › Fig 4/Fig 4D/IkB.png]

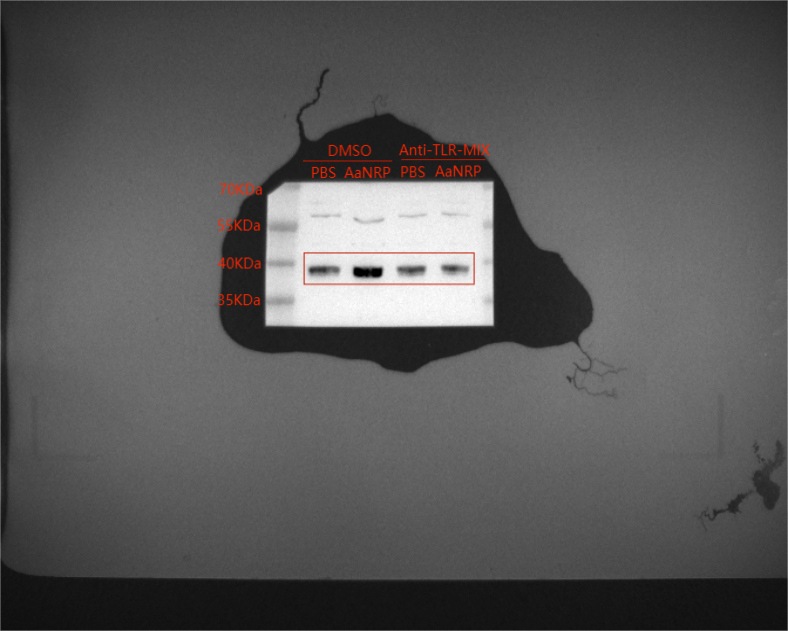

Supplement: Supplementary file 5 — Source Data Fig. 4 [file 44318_2024_56_MOESM5_ESM.zip › Fig 4/Fig 4D/p-IkB.png]

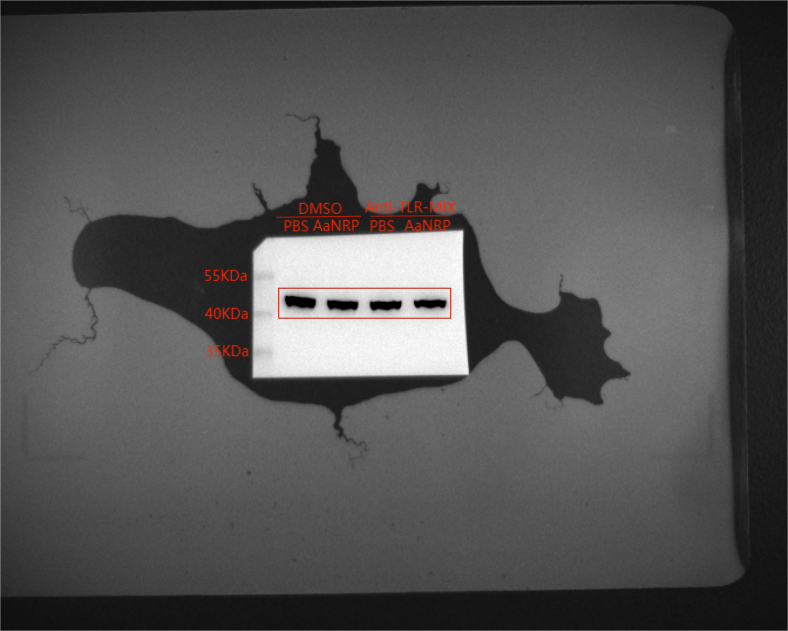

Supplement: Supplementary file 5 — Source Data Fig. 4 [file 44318_2024_56_MOESM5_ESM.zip › Fig 4/Fig 4D/┬aA╠ä-actin.png]

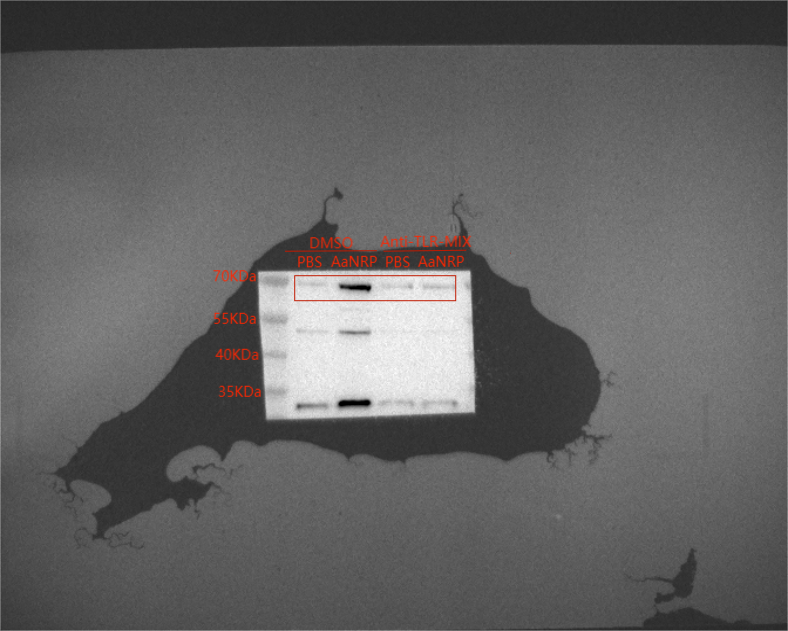

Supplement: Supplementary file 5 — Source Data Fig. 4 [file 44318_2024_56_MOESM5_ESM.zip › Fig 4/Fig 4D/p-p65.png]

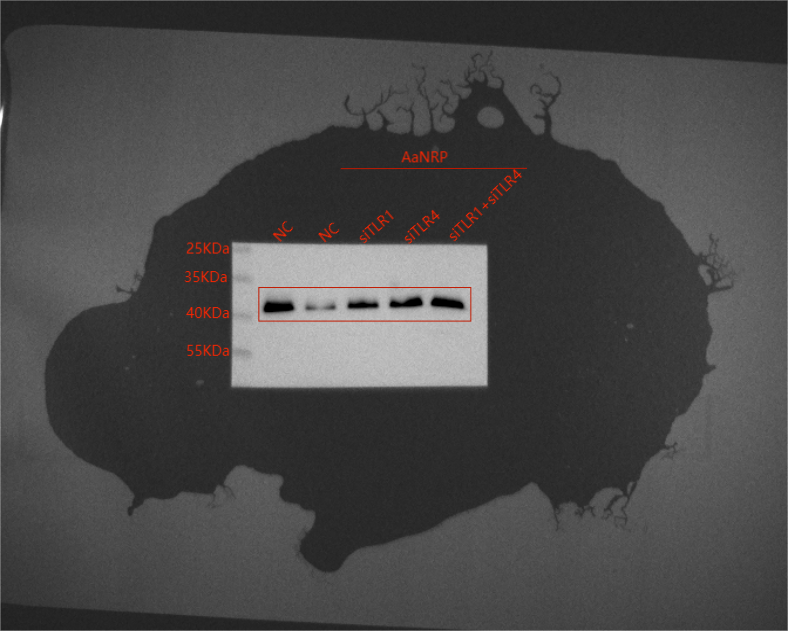

Supplement: Supplementary file 5 — Source Data Fig. 4 [file 44318_2024_56_MOESM5_ESM.zip › Fig 4/Fig 4G/IkB.png]

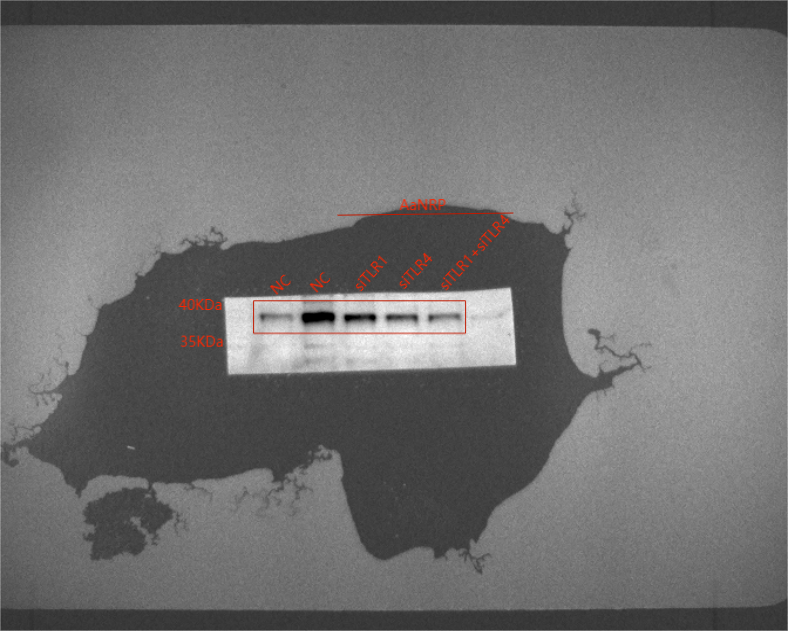

Supplement: Supplementary file 5 — Source Data Fig. 4 [file 44318_2024_56_MOESM5_ESM.zip › Fig 4/Fig 4G/p-IkB.png]

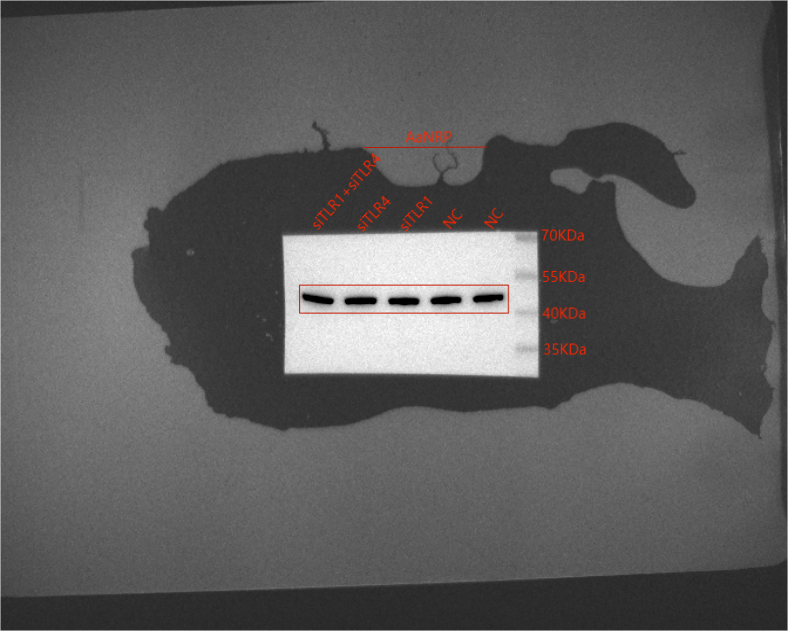

Supplement: Supplementary file 5 — Source Data Fig. 4 [file 44318_2024_56_MOESM5_ESM.zip › Fig 4/Fig 4G/┬aA╠ä-actin.png]

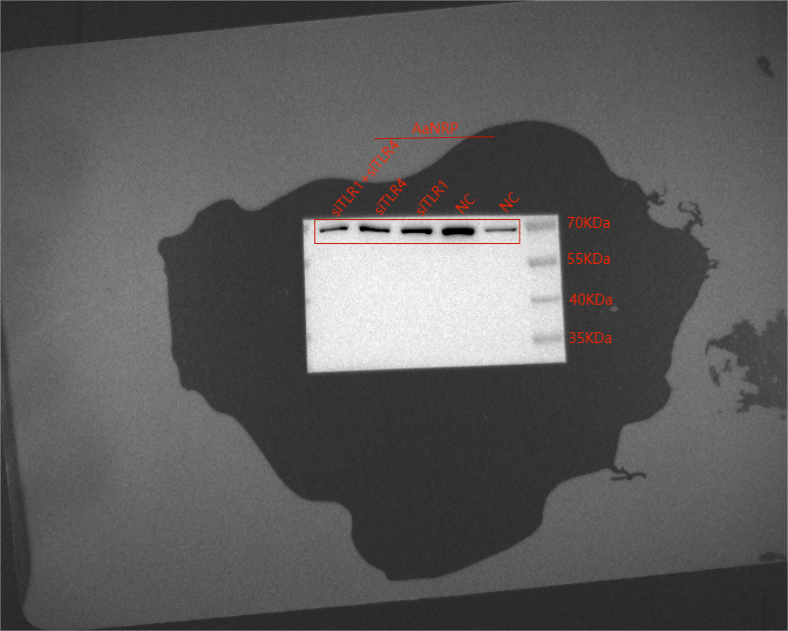

Supplement: Supplementary file 5 — Source Data Fig. 4 [file 44318_2024_56_MOESM5_ESM.zip › Fig 4/Fig 4G/p-p65.png]

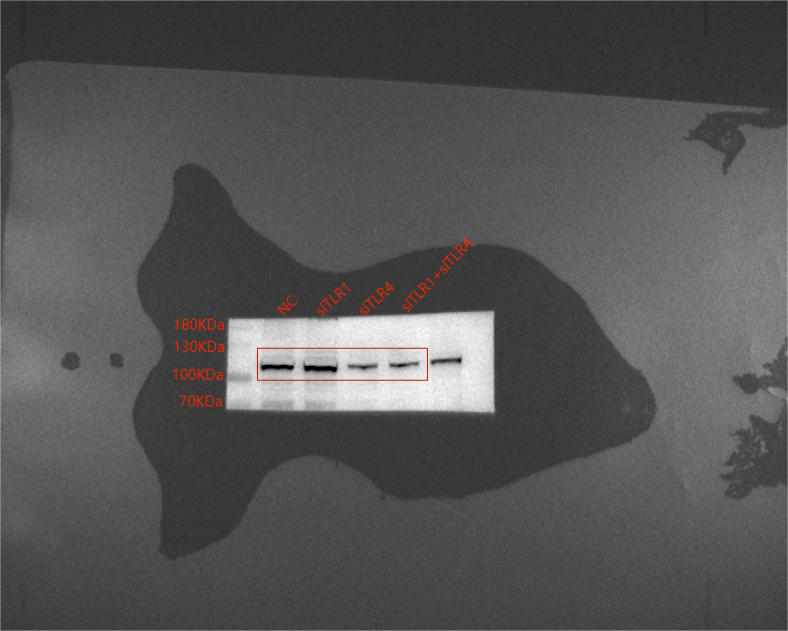

Supplement: Supplementary file 5 — Source Data Fig. 4 [file 44318_2024_56_MOESM5_ESM.zip › Fig 4/Fig 4F/TLR4 silencing by siRNA.png]

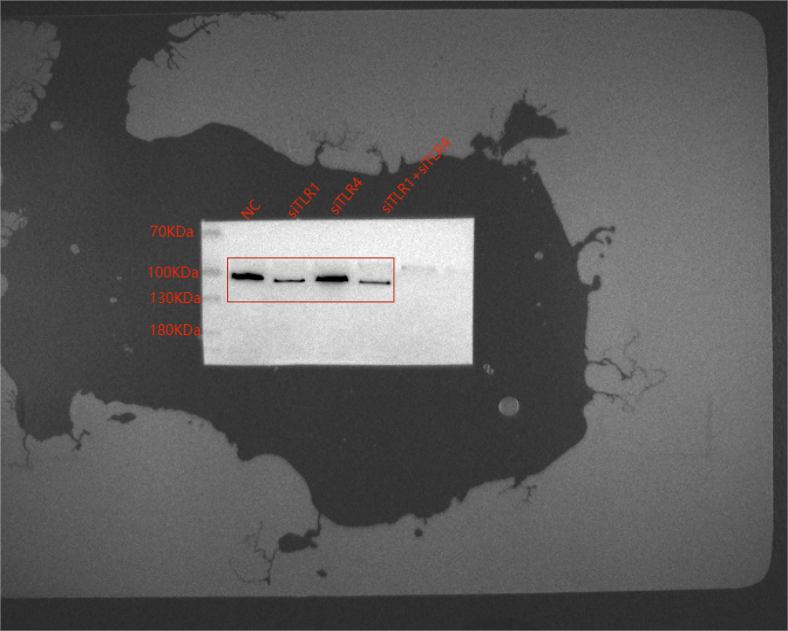

Supplement: Supplementary file 5 — Source Data Fig. 4 [file 44318_2024_56_MOESM5_ESM.zip › Fig 4/Fig 4F/TLR1 silencing by siRNA.png]

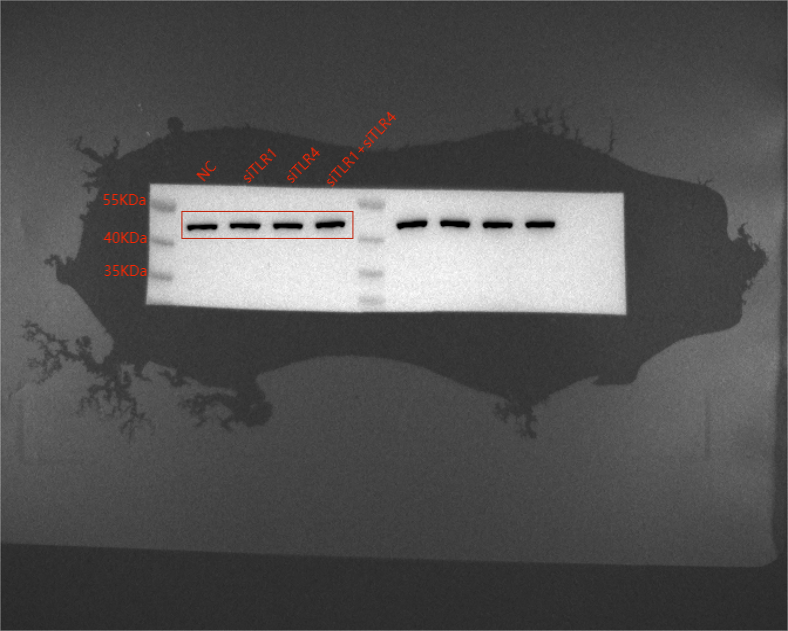

Supplement: Supplementary file 5 — Source Data Fig. 4 [file 44318_2024_56_MOESM5_ESM.zip › Fig 4/Fig 4F/┬aA╠ä-actin.png]

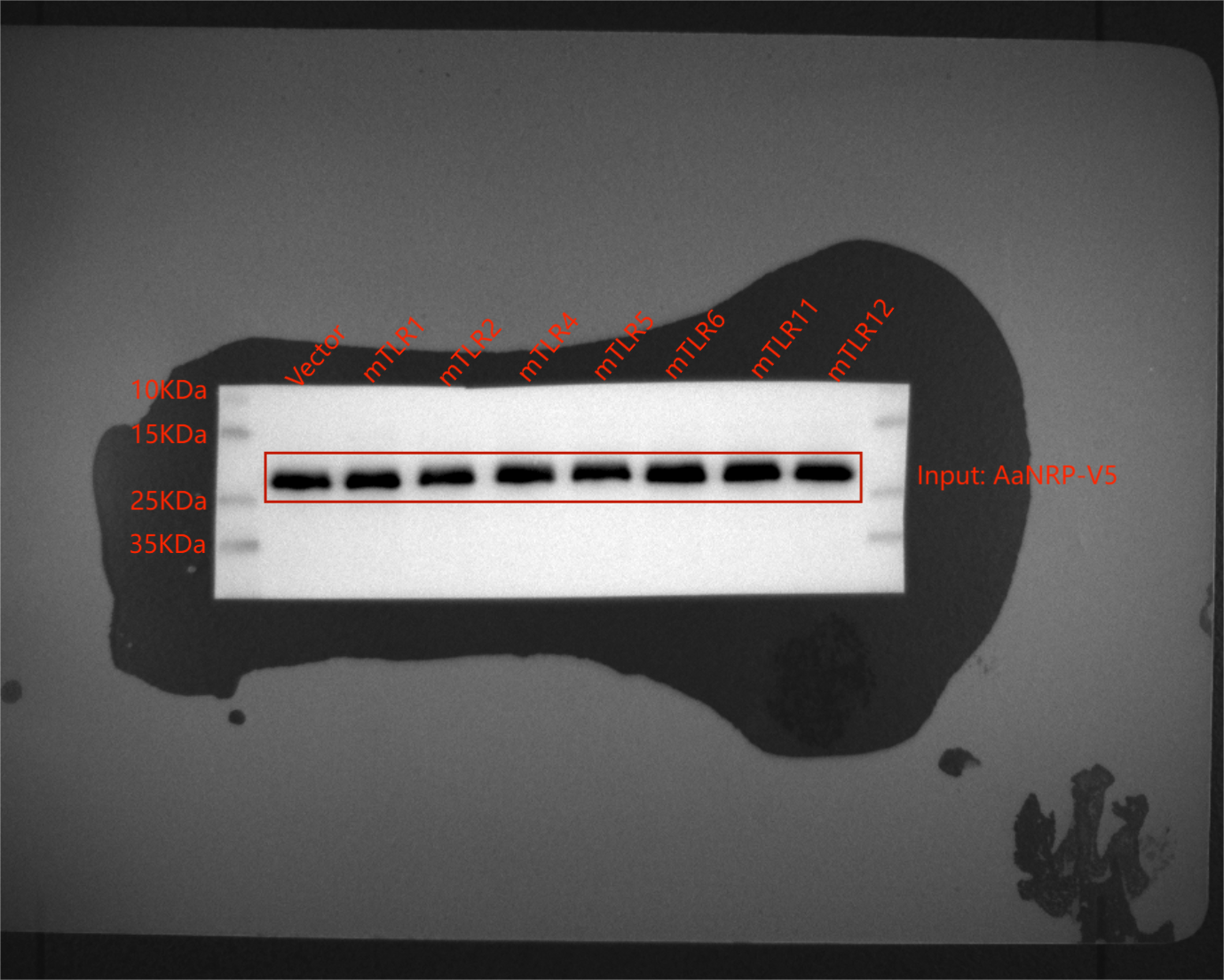

Supplement: Supplementary file 5 — Source Data Fig. 4 [file 44318_2024_56_MOESM5_ESM.zip › Fig 4/Fig 4A/Fig 4A (i) murine/AaNRP-V5 input.png]

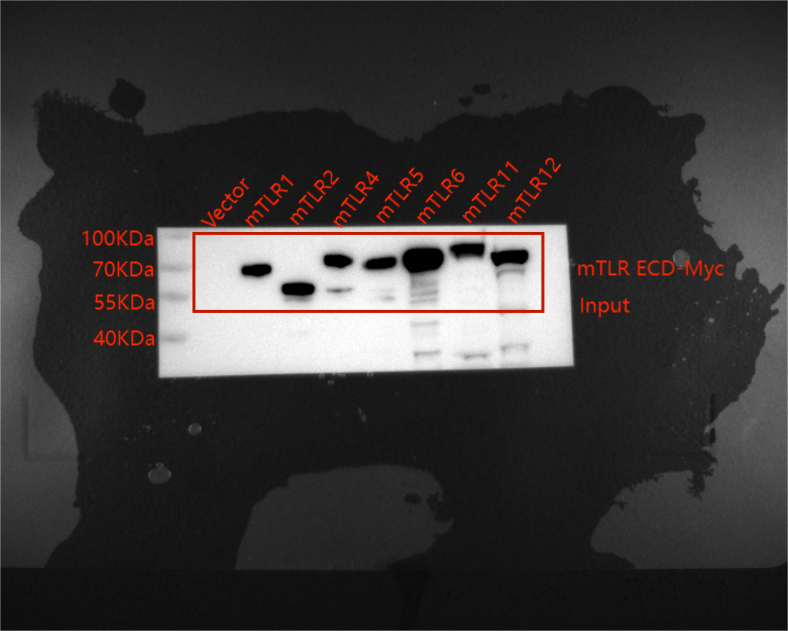

Supplement: Supplementary file 5 — Source Data Fig. 4 [file 44318_2024_56_MOESM5_ESM.zip › Fig 4/Fig 4A/Fig 4A (i) murine/mTLR ECD-Myc Input.png]

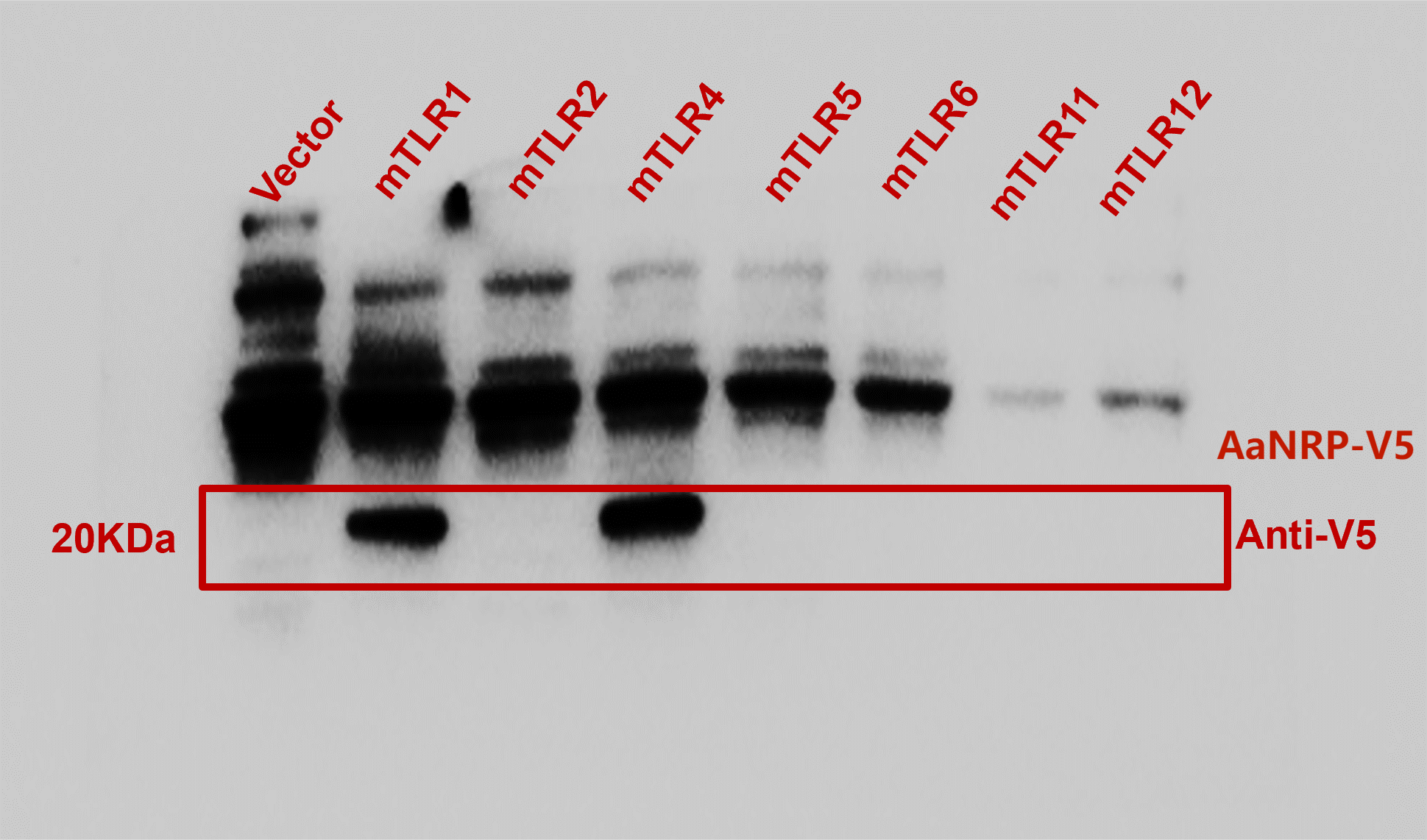

Supplement: Supplementary file 5 — Source Data Fig. 4 [file 44318_2024_56_MOESM5_ESM.zip › Fig 4/Fig 4A/Fig 4A (i) murine/AaNRP-V5 IP replicate 1.png]

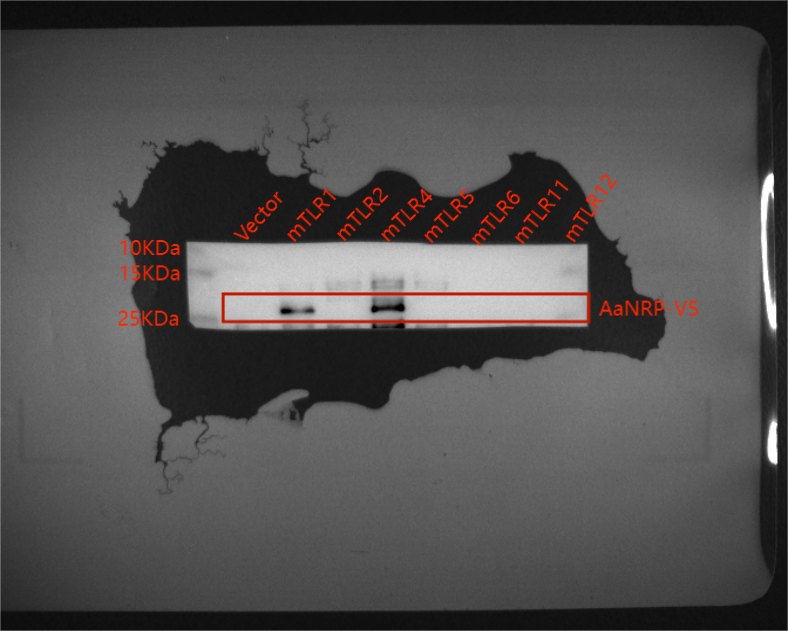

Supplement: Supplementary file 5 — Source Data Fig. 4 [file 44318_2024_56_MOESM5_ESM.zip › Fig 4/Fig 4A/Fig 4A (i) murine/AaNRP-V5 IP replicate 2.png]

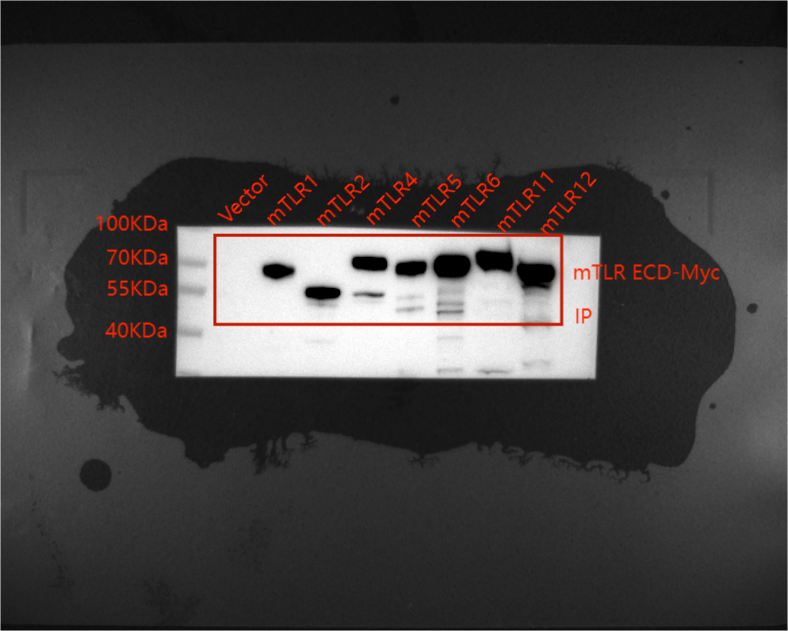

Supplement: Supplementary file 5 — Source Data Fig. 4 [file 44318_2024_56_MOESM5_ESM.zip › Fig 4/Fig 4A/Fig 4A (i) murine/mTLR ECD-Myc IP.png]

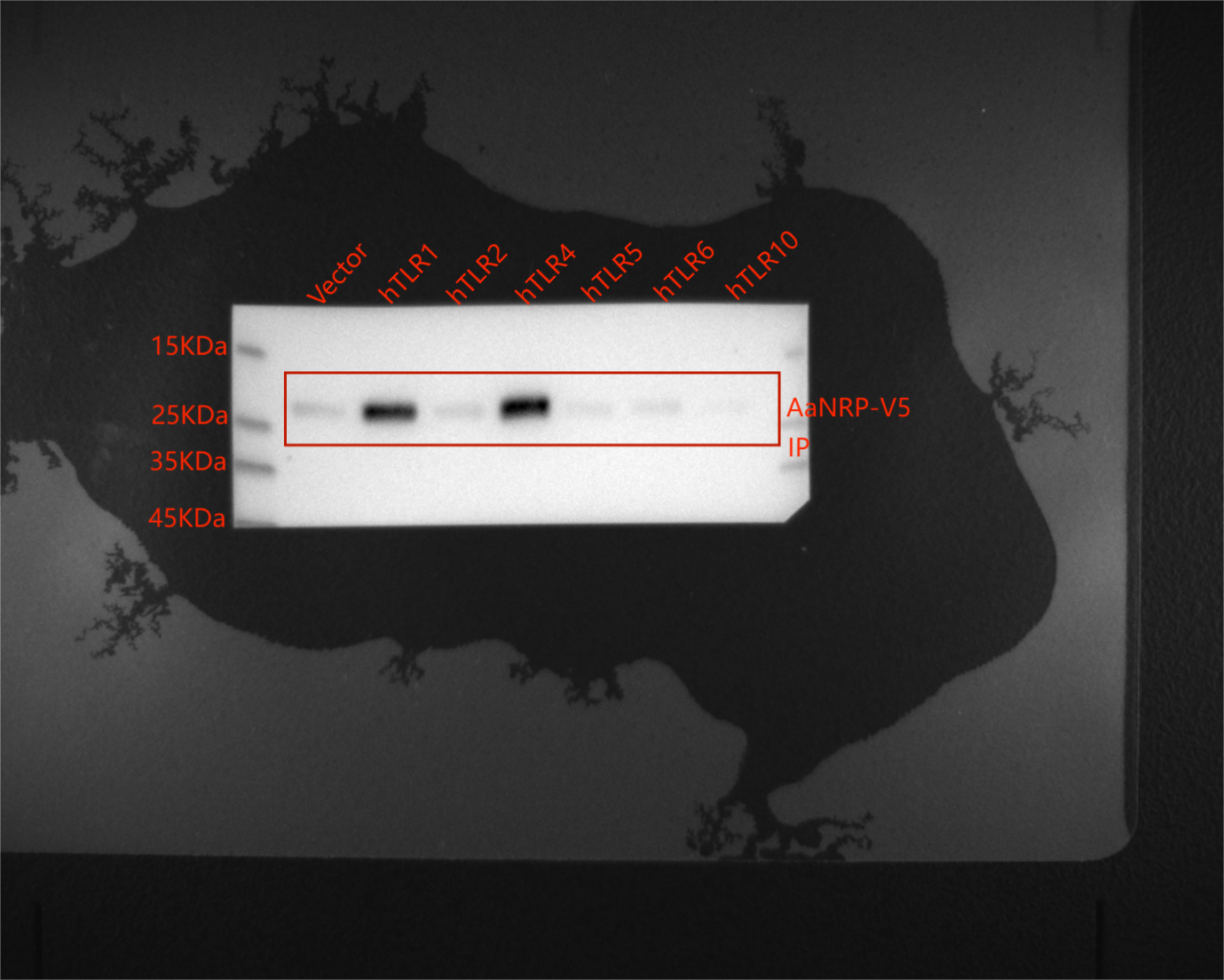

Supplement: Supplementary file 5 — Source Data Fig. 4 [file 44318_2024_56_MOESM5_ESM.zip › Fig 4/Fig 4A/Fig 4A (ii) human/AaNRP-V5 IP.png]

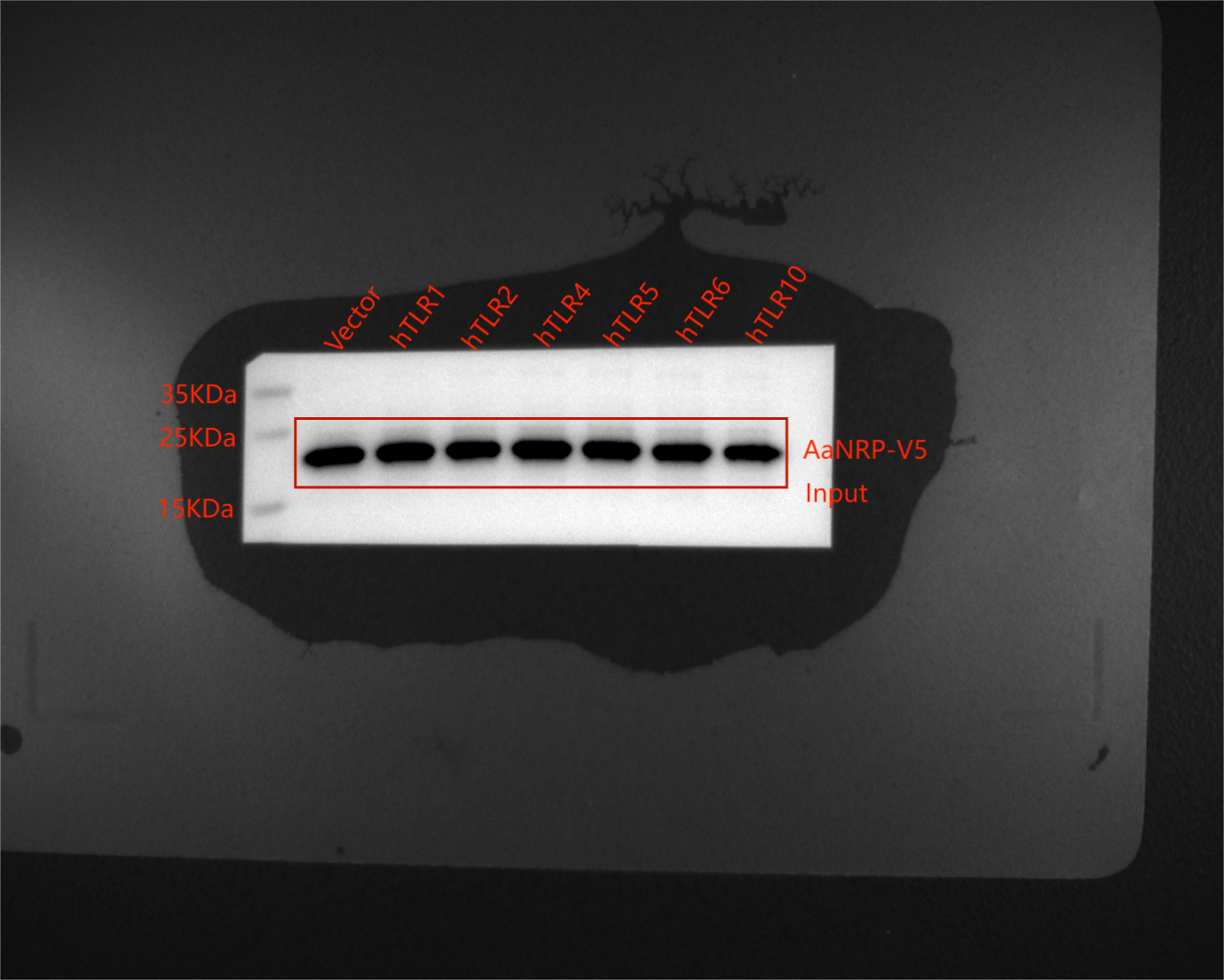

Supplement: Supplementary file 5 — Source Data Fig. 4 [file 44318_2024_56_MOESM5_ESM.zip › Fig 4/Fig 4A/Fig 4A (ii) human/AaNRP-V5 Input.png]

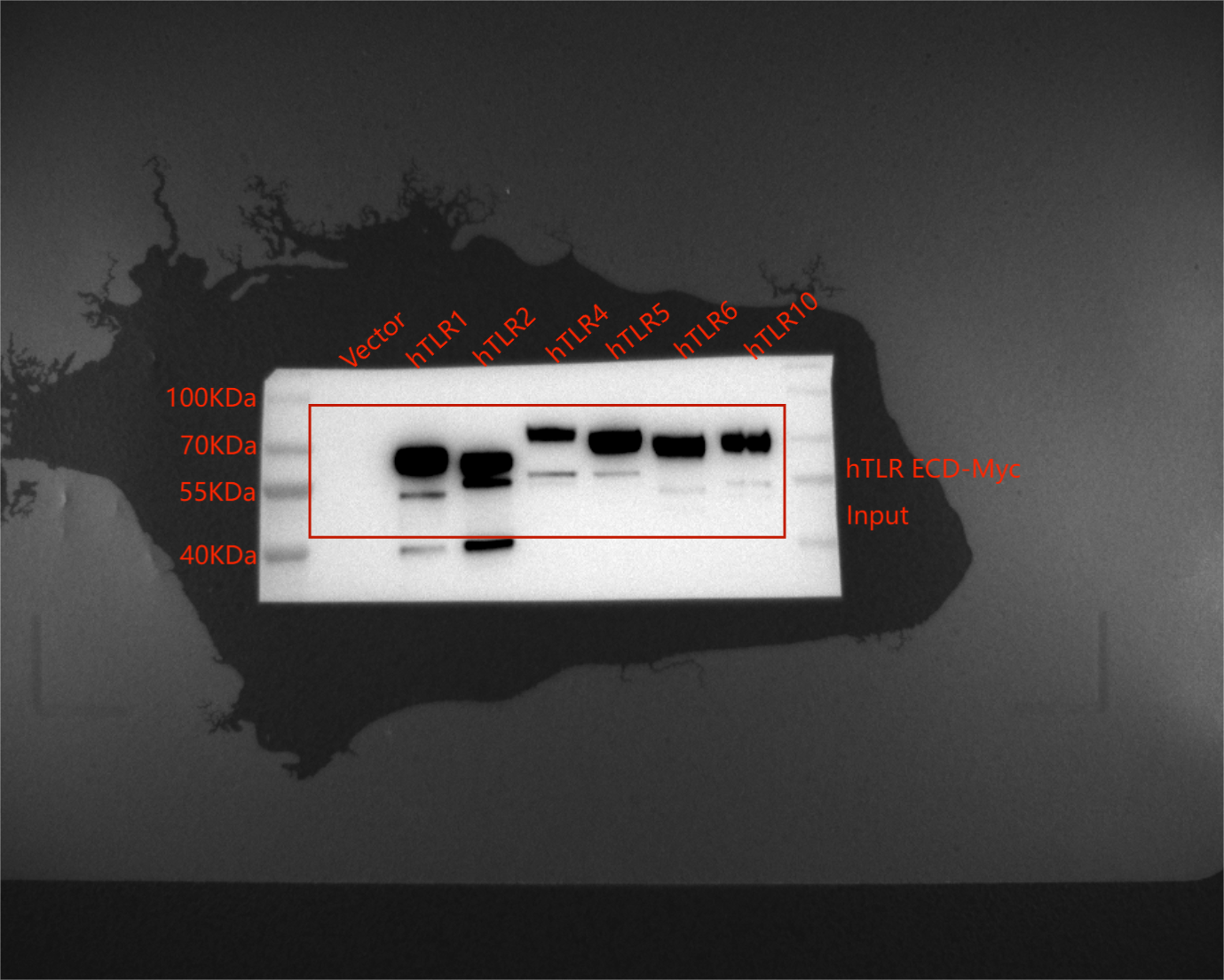

Supplement: Supplementary file 5 — Source Data Fig. 4 [file 44318_2024_56_MOESM5_ESM.zip › Fig 4/Fig 4A/Fig 4A (ii) human/human TLR ECD-Myc, Input.png]

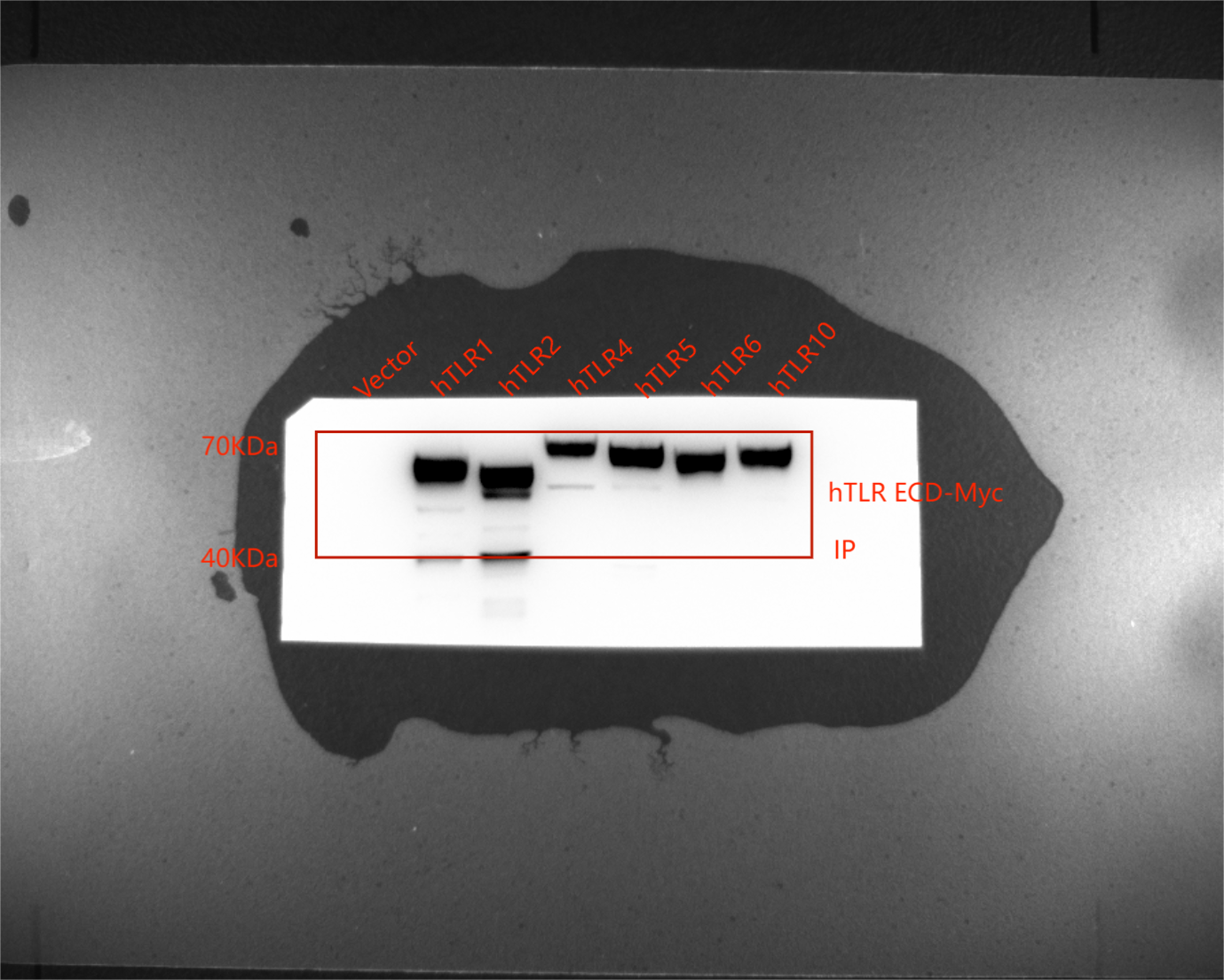

Supplement: Supplementary file 5 — Source Data Fig. 4 [file 44318_2024_56_MOESM5_ESM.zip › Fig 4/Fig 4A/Fig 4A (ii) human/human TLR ECD-Myc, IP by anti-Myc.png]

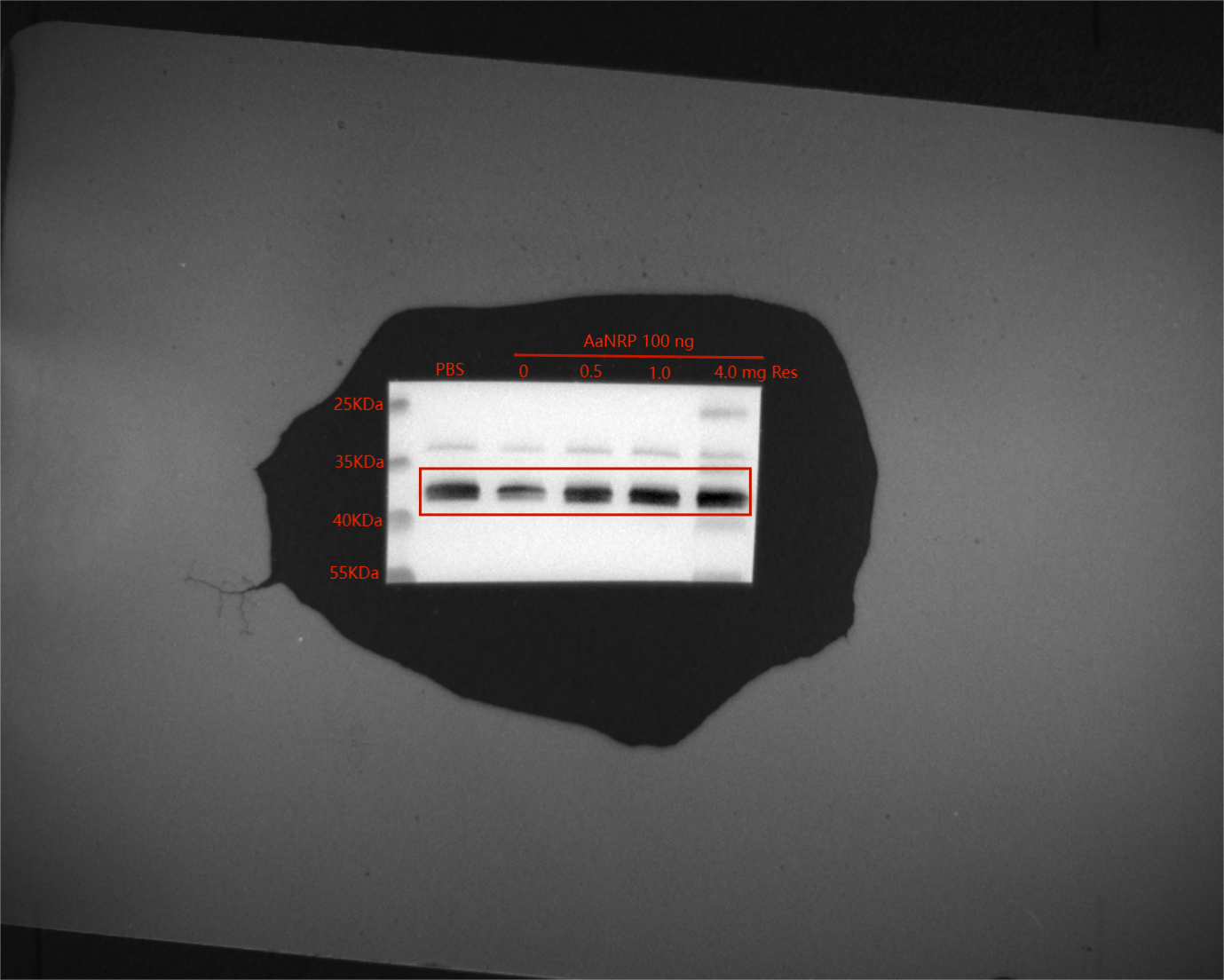

Supplement: Supplementary file 8 — Source Data Fig. 7 [file 44318_2024_56_MOESM8_ESM.zip › Fig 7/Fig 7B/IkB.png]

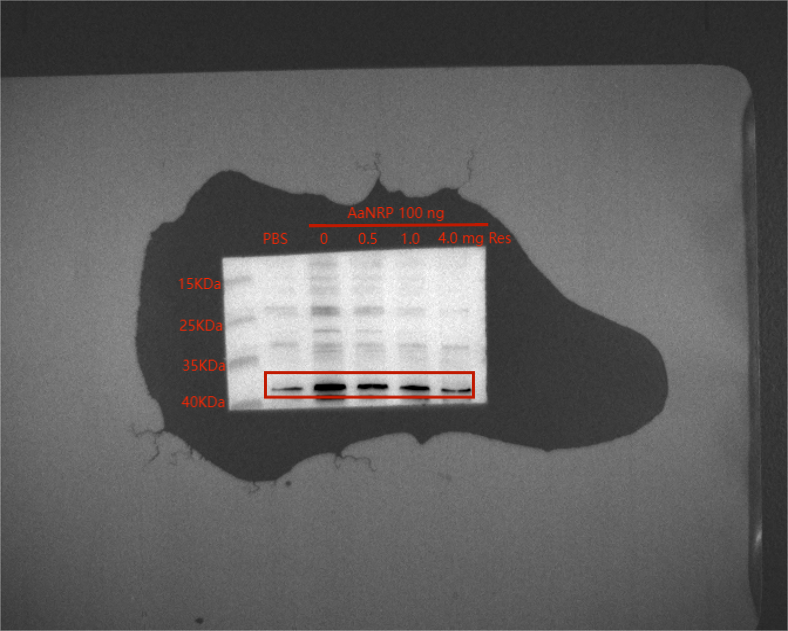

Supplement: Supplementary file 8 — Source Data Fig. 7 [file 44318_2024_56_MOESM8_ESM.zip › Fig 7/Fig 7B/p-IkB.png]

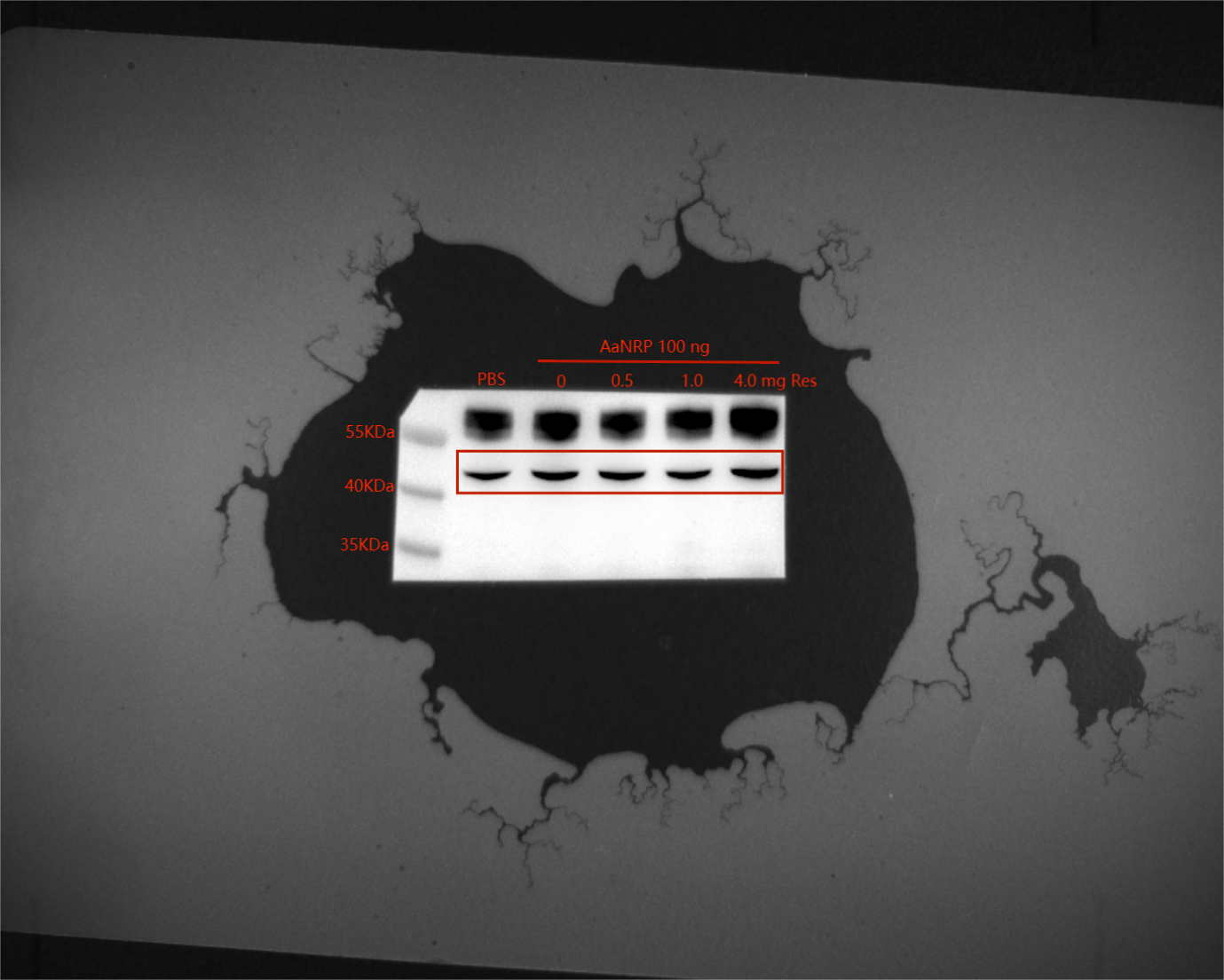

Supplement: Supplementary file 8 — Source Data Fig. 7 [file 44318_2024_56_MOESM8_ESM.zip › Fig 7/Fig 7B/┬aA╠ä-actin.png]

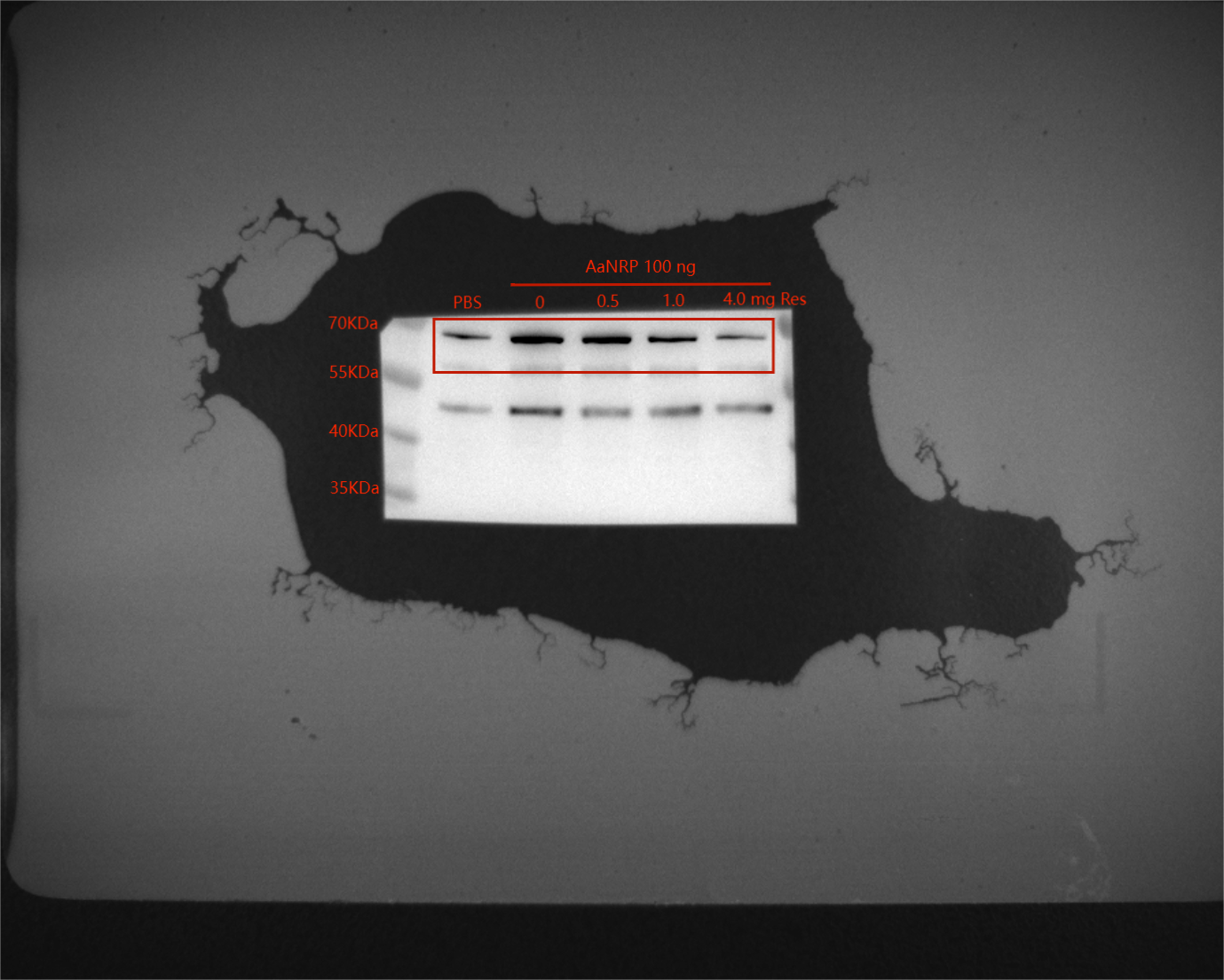

Supplement: Supplementary file 8 — Source Data Fig. 7 [file 44318_2024_56_MOESM8_ESM.zip › Fig 7/Fig 7B/p-p65.png]
