# Supplementary material for: A mosquito salivary protein-driven influx of myeloid cells facilitates flavivirus transmission
Source: EMBO J. 2024 Feb 20;43(9):2. doi: 10.1038/s44318-024-00056-x (PMC11066113; doi:10.1038/s44318-024-00056-x)
Supplement: Supplementary file 5 — Source Data Fig. 4 [file 44318_2024_56_MOESM5_ESM.zip › Fig 4/Fig 4B/Fig 4B.pptx]

## Slide 1
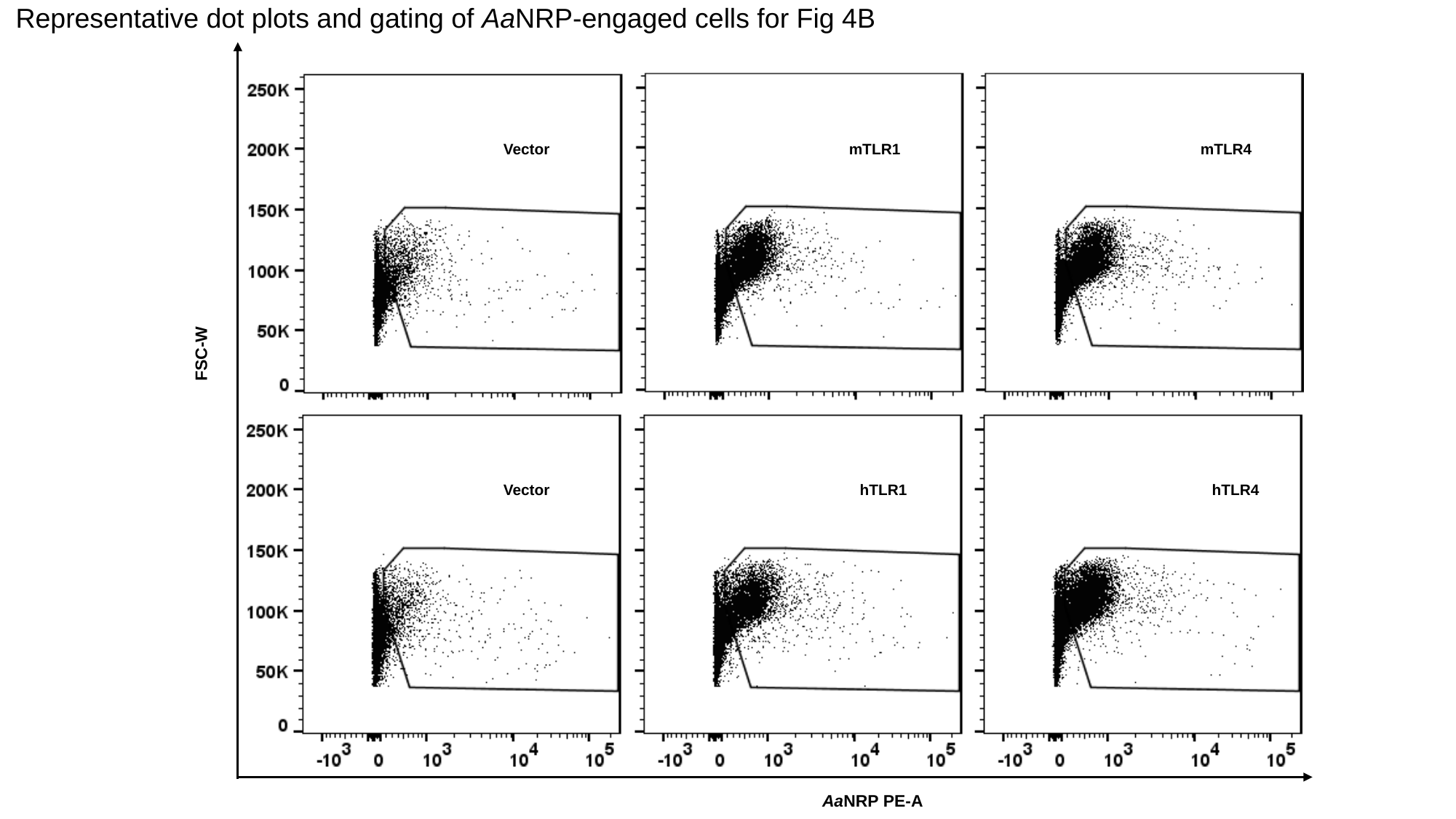

Representative dot plots and gating of AaNRP-engaged cells for Fig 4B
mTLR1
mTLR4
Vector
hTLR4
Vector
hTLR1
FSC-W
AaNRP PE-A
