# Supplementary material for: A mosquito salivary protein-driven influx of myeloid cells facilitates flavivirus transmission
Source: EMBO J. 2024 Feb 20;43(9):2. doi: 10.1038/s44318-024-00056-x (PMC11066113; doi:10.1038/s44318-024-00056-x)
Supplement: Supplementary file 10 — Expanded View Figures [file 44318_2024_56_MOESM10_ESM.pdf]

Expanded View Figures

Figure EV1. *Aa*NRP-mediated neutrophil influx and the specific expression of *Aa*NRP in *A. aegypti* mosquitoes, related to Fig. 1.

(**A**) Dot-plot graphs showing the flow cytometric analysis of neutrophils ($CD45^+CD11b^+Ly6G^+$) in mouse footpad skin at 4 h post inoculation (hpi, related to Fig. 1C). 129 Sv/Ev mice were intradermally inoculated with 100 ng *Aa*NRP or PBS in the hind footpads as negative controls. (**B**) Dot-plot graphs showing the flow cytometric analysis of neutrophils in mouse footpad skin at 4 hpi, 12 hpi, 24 hpi and 48 hpi (related to Fig. 1D). 129 Sv/Ev mice were intradermally inoculated with 100 ng *Aa*NRP, equivalent amount of mosquito saliva as a positive control, or PBS, heat-inactivated *Aa*NRP (hi*Aa*NRP) and a noninflammatory control (NIC) salivary protein encoded by *AAEL009524* as negative controls. (**C**) IHC staining of the specific neutrophil marker Ly6G in footpad sections at 4 hpi and 12 hpi. 129 Sv/Ev mice were intradermally inoculated with 100 ng *Aa*NRP or PBS in the hind footpads as negative controls. Red arrows indicate neutrophils, scale bar 20 μm. (**D**) Quantification of the number of $Ly6G^+$ cells per mm^2 of footpad skin sections at 4 hpi and 12 hpi. (**E**) Hematoxylin-eosin (HE) staining of neutrophils in footpad skin at 4 hpi and 12 hpi. Red arrows indicate neutrophils, and regions circled with dashed red lines denote intravascular lumens, cale bar 20 μm. (**F–H**) RNA expression level of *AaNRP* in different mosquito tissues (**F**), in salivary glands with female specificity (**G**) and in salivary glands at 24 h post blood meal (**H**). The RNA level of *AaNRP* was detected by qPCR and expressed as the ratio of β-actin with $2^{-\Delta Ct}$ normalization. (**D**, **F–H**) Data are expressed as the mean ± SEM, and each dot represents an individual mouse (**D**) or mosquito (**F–H**). The two-way ANOVA and multiple *t* tests (**D**), one-way ANOVA (**F**), and unpaired *t* test (**G**, **H**) were used for the statistical analyses. All experiments were reproduced at least twice. $**p < 0.01$, $***p < 0.001$, $****p < 0.0001$.

 © The Author(s)

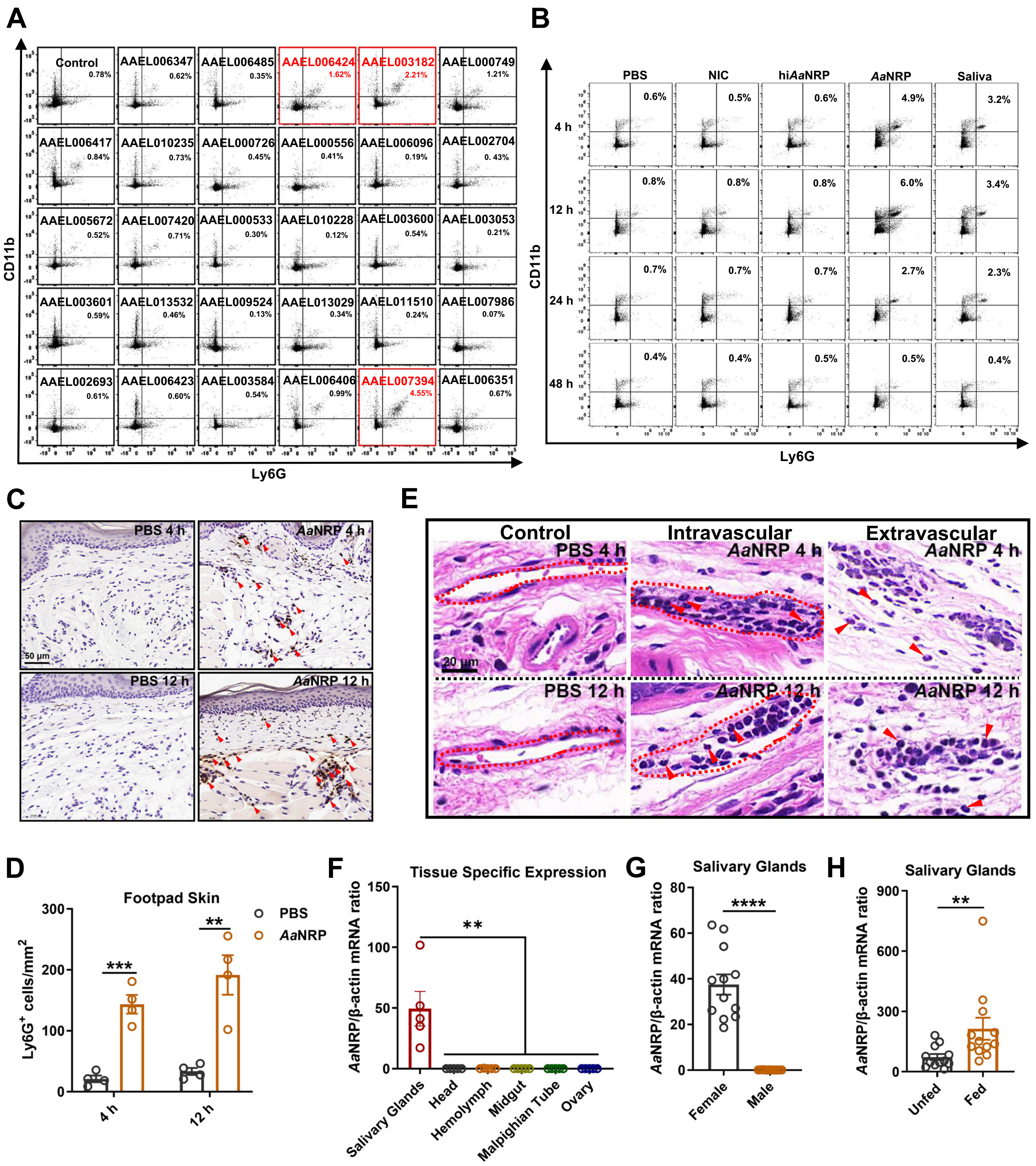
A
Control
AAEL006347
AAEL006485
AAEL006424
AAEL003182
AAEL000749
AAEL006417
AAEL010235
AAEL000726
AAEL000556
AAEL006096
AAEL002704
AAEL005672
AAEL007420
AAEL000533
AAEL010228
AAEL003600
AAEL003053
AAEL003601
AAEL013532
AAEL009524
AAEL013029
AAEL011510
AAEL007986
AAEL002693
AAEL006423
AAEL003584
AAEL006406
AAEL007394
AAEL006351
CD11b
Ly6G
B
PBS
NIC
hiAaNRP
AaNRP
Saliva
4 h
12 h
24 h
48 h
CD11b
Ly6G
C
PBS 4 h
AaNRP 4 h
PBS 12 h
AaNRP 12 h
50 μm
E
Control
Intravascular
Extravascular
PBS 4 h
AaNRP 4 h
PBS 12 h
AaNRP 12 h
20 μm
D
Footpad Skin
PBS
AaNRP
Ly6G+ cells/mm2
4 h
12 h
F
Tissue Specific Expression
AaNRP/β-actin mRNA ratio
Salivary Glands
Head
Hemolymph
Midgut
Malpighian Tube
Ovary
G
Salivary Glands
AaNRP/β-actin mRNA ratio
Female
Male
H
Salivary Glands
AaNRP/β-actin mRNA ratio
Unfed
Fed

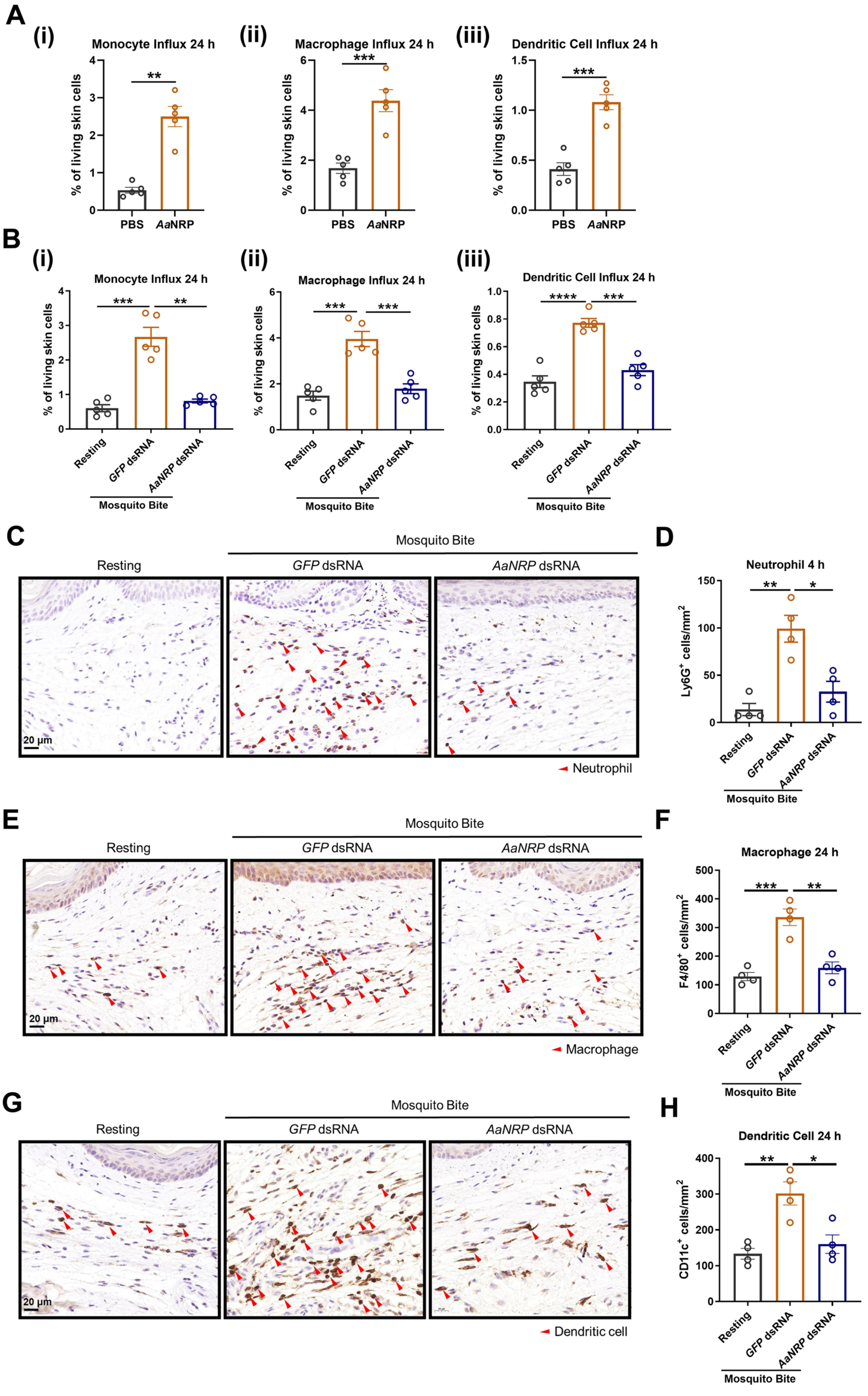
A
(i) Monocyte Influx 24 h
% of living skin cells
PBS AaNRP
**
(ii) Macrophage Influx 24 h
% of living skin cells
PBS AaNRP
***
(iii) Dendritic Cell Influx 24 h
% of living skin cells
PBS AaNRP
***
B
(i) Monocyte Influx 24 h
(ii) Macrophage Influx 24 h
(iii) Dendritic Cell Influx 24 h
% of living skin cells
Resting
GFP dsRNA
AaNRP dsRNA
Mosquito Bite
C
Mosquito Bite
Resting
GFP dsRNA
AaNRP dsRNA
20 μm
Neutrophil
D
Neutrophil 4 h
Ly6G+ cells/mm2
E
Macrophage
F
Macrophage 24 h
F4/80+ cells/mm2
G
Dendritic cell
H
Dendritic Cell 24 h
CD11c+ cells/mm2

 © The Author(s)

Figure EV2. *Aa*NRP induces myeloid cell influx, related to Fig. 1.

(**A**) *Aa*NRP-induced influx of monocytes (i), macrophages (ii), and dendritic cells (DCs, iii) toward murine footpad skin at 24 hpi. 129 Sv/Ev mice were intradermally inoculated with 100 ng *Aa*NRP or PBS in the hind footpads as negative controls. At 24 hpi, murine footpads were sampled to measure the percentages of myeloid cells in all living skin cells by flow cytometry. (**B**) Influence of *AaNRP* silencing on mosquito bite-induced influx of monocytes (i), macrophages (ii), and DCs (iii) at 24 h post bite (hpb). *A. aegypti* mosquitoes were intrathoracically injected with 1 µg/300 nL *AaNRP* dsRNA or *GFP* dsRNA (negative control). Three days later, mosquitoes were allowed to bite the hind footpads of 129 Sv/Ev mice (each hind footpad was bitten by five mosquitoes) and the unbitten mice served as the resting controls. At 24 hpb, murine footpads were sampled to measure the percentages of myeloid cells in all living skin cells by flow cytometry. (**C–H**) Influence of *AaNRP* silencing on mosquito bite-induced myeloid cell influx assessed by immunohistochemical (IHC) staining. *A. aegypti* mosquitoes were intrathoracically injected with 1 µg/300 nL *AaNRP* dsRNA or *GFP* dsRNA (negative control). Three days later, mosquitoes were allowed to bite the hind footpads of 129 Sv/Ev mice (each hind footpad was bitten by five mosquitoes) and the unbitten mice served as the resting control. At 4 hpb and 24 hpb, murine footpads were dissected for IHC staining of neutrophils at 4 hpb (**C**) and of macrophages (**E**) and DCs (**G**) at 24 hpb. (**C**) IHC staining of the neutrophil-specific marker Ly6G indicated by red arrows, scale bar 20 µm. (**D**) Quantification of the counts of neutrophils per mm^2 of footpad section. Four random scopes were sampled from each mouse, and the mean of the four scopes was used to represent the mouse. (**E**) IHC staining of the macrophage-specific marker F4/80 indicated by red arrows, scale bar 20 µm. (**F**) Quantification of the counts of macrophages per mm^2 of footpad section. (**G**) IHC staining of the DC-specific marker CD11c indicated by red arrows, scale bar 20 µm. (**H**) Quantification of the counts of DCs per mm^2 of footpad section. (**A**, **B**, **D**, **F**, **H**) Data are expressed as the mean ± SEM, and each dot represents an individual mouse. The unpaired *t* test (**A**) and the one-way ANOVA and multiple *t* tests (**B**, **D**, **F**, **H**) were used for statistical analyses. All experiments were reproduced at least twice. $^*p < 0.05$, $^{**}p < 0.01$, $^{***}p < 0.001$, $^{****}p < 0.0001$.

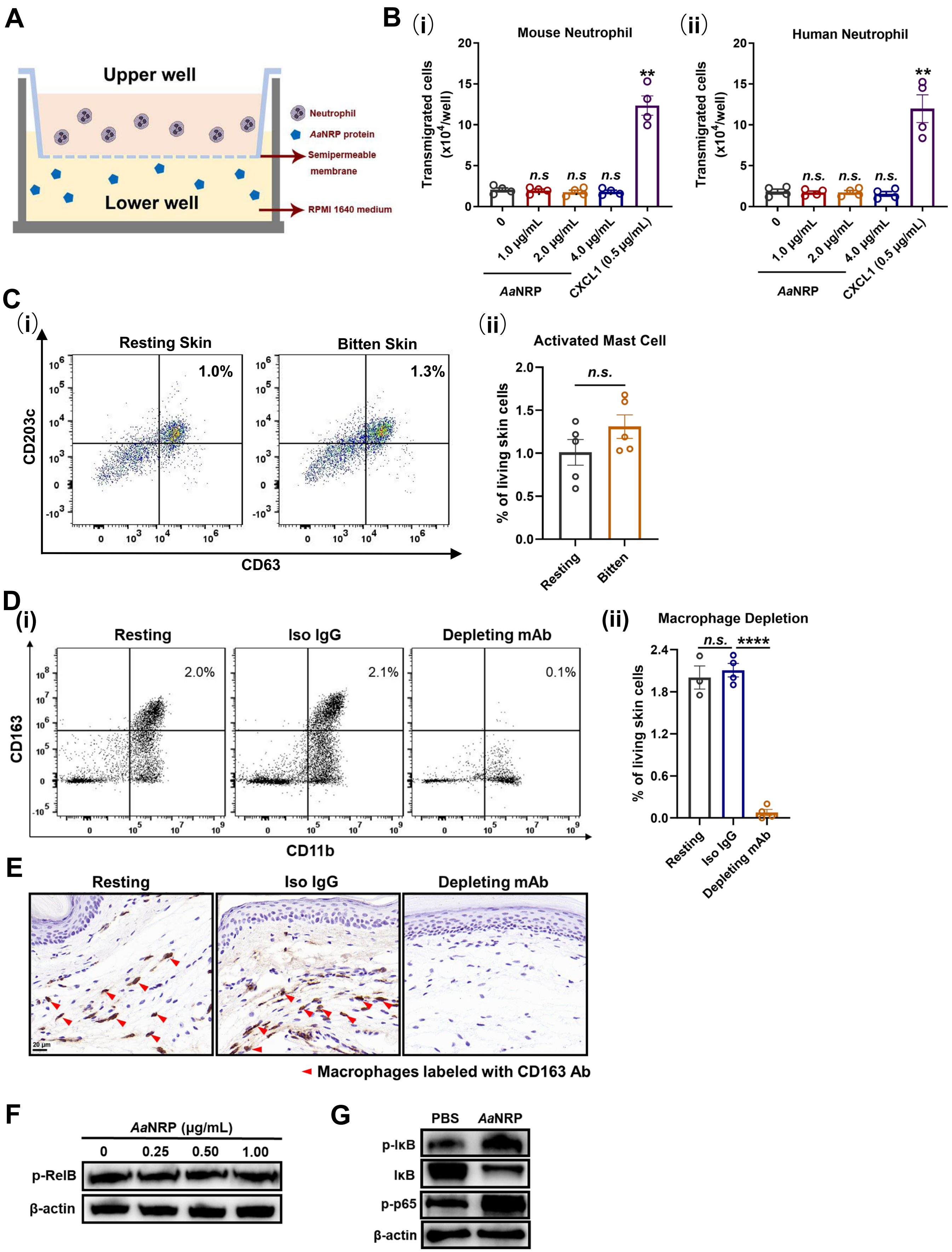
A
Upper well
Lower well
Neutrophil
AaNRP protein
Semipermeable membrane
RPMI 1640 medium
B (i) Mouse Neutrophil
(ii) Human Neutrophil
Transmigrated cells (x10^4/well)
0
1.0 μg/mL
2.0 μg/mL
4.0 μg/mL
CXCL1 (0.5 μg/mL)
AaNRP
n.s
**
C (i) Resting Skin
Bitten Skin
1.0%
1.3%
CD203c
CD63
(ii) Activated Mast Cell
% of living skin cells
Resting
Bitten
n.s.
D (i) Resting
Iso IgG
Depleting mAb
2.0%
2.1%
0.1%
CD163
CD11b
(ii) Macrophage Depletion
% of living skin cells
n.s.
****
Resting
Iso IgG
Depleting mAb
E Resting
Iso IgG
Depleting mAb
20 μm
Macrophages labeled with CD163 Ab
F AaNRP (μg/mL)
0
0.25
0.50
1.00
p-RelB
β-actin
G PBS
AaNRP
p-IκB
IκB
p-p65
β-actin

Figure EV3. Influence of *Aa*NRP on the chemotaxis, activation, and inflammatory signaling of skin innate immunocytes, related to Figs. 2 and 3.

(**A–C**) Transwell assays exploring the direct chemotaxis of neutrophils by *Aa*NRP. (**A**) Study design for the neutrophil transmigration assay. A transwell with a 5.0 μm pore was used. Neutrophils (2×10^5) isolated from human and murine peripheral blood and suspended in 250 μL RPMI 1640 medium supplemented with 2% FBS were added to the upper chamber. RPMI 1640 (600 μL) containing 0, 1.0, 2.0, or 4.0 μg/mL *Aa*NRP or 0.5 μg/mL mouse and human CXCL1 (as positive controls) supplemented with 2% FBS was added to the lower chambers. Three hours later, the neutrophils that transmigrated into the lower chamber or adhered to the basolateral side of the upper chamber were collected and counted using a cell counter (Celldrop, FL/BF, Denovix). (**B**) Counts of mouse (i) and human (ii) neutrophils that transmigrated to the lower chambers under different concentrations of *Aa*NRP or CXCL1. (**C**) Influence of *Aa*NRP on skin mast cell activation as assessed by flow cytometry. (i) Dot plots showing the activated mast cells that are $CD63^{+}CD203c^{+}$. (ii) Percentages of the activated mast cells in resting and mosquito-bitten skin. Mice were bitten by mosquitoes in the hind footpads (one footpad was bitten by five mosquitoes) or left unbitten as the resting controls. Four hours later, the footpad skin was collected and processed for flow cytometric analysis of mast cell activation. (**D–E**) Verification of resident macrophage depletion in murine footpad skin by flow cytometry (**D**) and IHC (**E**). (**D**, i) Dot plots showing the skin resident macrophages ($CD45^{+}CD11b^{+}CD163^{+}$). (**D**, ii) Percentages of skin resident macrophages within all the living skin cells. (**E**) IHC staining of resident macrophages in murine footpad skin with a specific biomarker CD163 indicated by red arrows, scale bar 20 μm. 129 Sv/Ev mice were intraperitoneally injected with 1 mg anti-F4/80 monoclonal antibody (depleting mAb) or 1 mg rat isotypic IgG2b antibody at 2 and 1 days before sampling. Footpads were collected from untreated (resting), isotype IgG-injected, and anti-F4/80 mAb-injected mice and were then used for flow cytometry and IHC analysis of skin resident macrophages. (**F**) Influence of *Aa*NRP on the expression of phosphorylated RelB (p-RelB). RAW264.7 cells were treated with 0, 0.25, 0.50, or 1.00 μg/mL *Aa*NRP in DMEM supplemented with 2% FBS for 4 h. Subsequently, the cells were collected and subjected to an immunoblotting assay to detect the protein abundance of p-RelB. (**G**) Influence of *Aa*NRP on the canonical NF-κB signaling in murine footpad. 129 Sv/Ev mice were intradermally injected with 100 ng *Aa*NRP or 20 μL PBS in the hind footpads with a very fine-tipped syringe. Four hours later, the footpads were collected for an immunoblotting assay to detect the protein abundance of some crucial signaling molecules in the canonical MyD88-NF-κB signaling axis. (**B–D**) Data are expressed as the mean ± SEM and each dot represents an individual mouse. The one-way ANOVA and multiple *t* tests (**B**, **D**) and unpaired *t* test (**C**) were used for statistical analyses. All experiments were reproduced at least twice. $^{**}p < 0.01$, $^{****}p < 0.0001$, n.s. not significant.

© The Author(s)

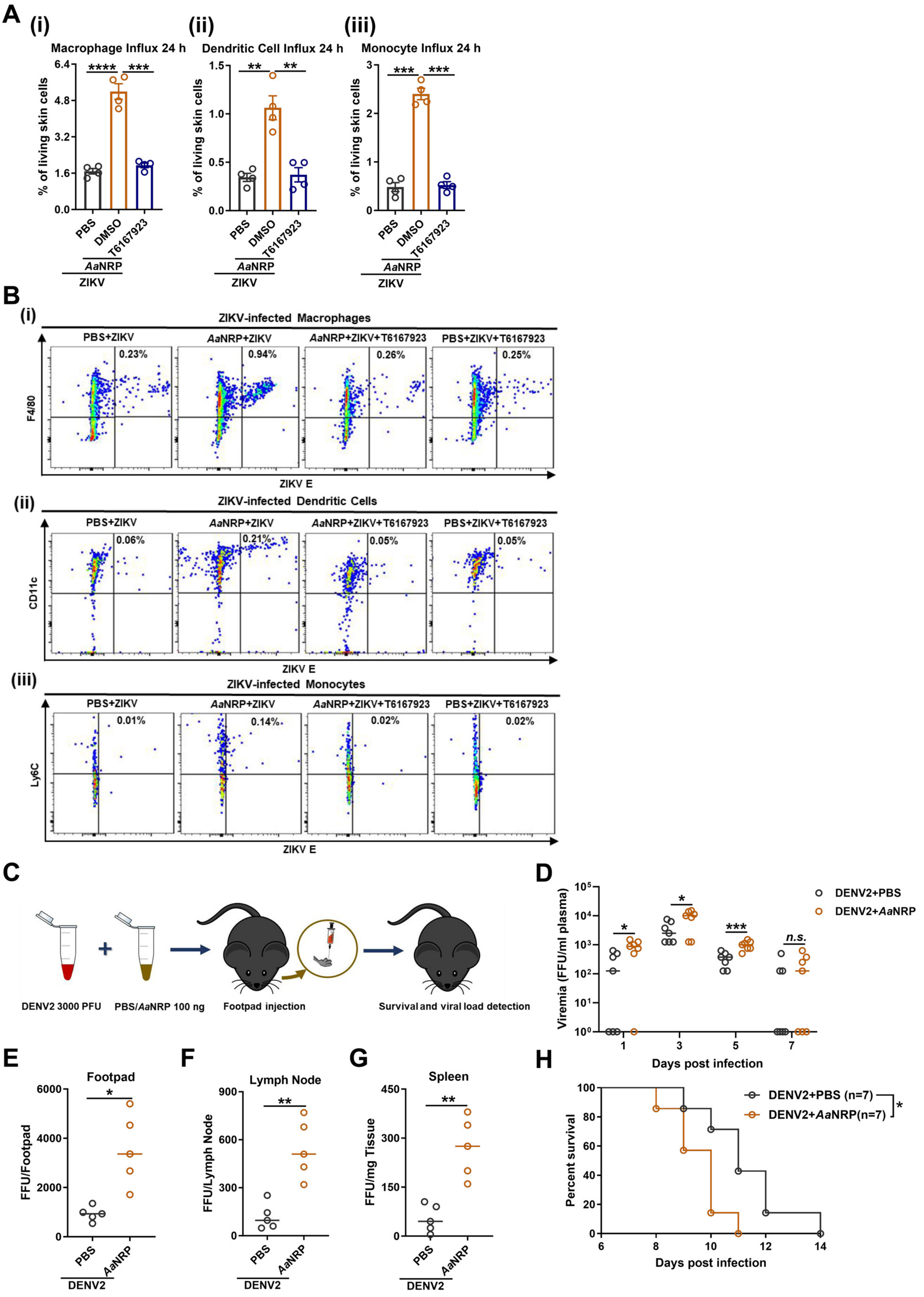
A
(i)
Macrophage Influx 24 h
% of living skin cells
****
***
PBS
DMSO
T6167923
AaNRP
ZIKV
(ii)
Dendritic Cell Influx 24 h
**
**
(iii)
Monocyte Influx 24 h
***
***
B
(i)
ZIKV-infected Macrophages
PBS+ZIKV
AaNRP+ZIKV
AaNRP+ZIKV+T6167923
PBS+ZIKV+T6167923
0.23%
0.94%
0.26%
0.25%
F4/80
ZIKV E
(ii)
ZIKV-infected Dendritic Cells
0.06%
0.21%
0.05%
0.05%
CD11c
(iii)
ZIKV-infected Monocytes
0.01%
0.14%
0.02%
0.02%
Ly6C
C
DENV2 3000 PFU
PBS/AaNRP 100 ng
Footpad injection
Survival and viral load detection
D
Viremia (FFU/ml plasma)
DENV2+PBS
DENV2+AaNRP
n.s.
Days post infection
E
Footpad
FFU/Footpad
*
DENV2
F
Lymph Node
FFU/Lymph Node
**
G
Spleen
FFU/mg Tissue
**
H
Percent survival
DENV2+PBS (n=7)
DENV2+AaNRP(n=7)
*
Days post infection

 © The Author(s)

Figure EV4. *Aa*NRP enhances flaviviral infection in mice, related to Figs. 5 and 6.

(**A**) Influx of macrophages (i), DCs (ii) and monocytes (iii) toward murine footpad skin at 24 hpi. Six-week-old female type I interferon receptor-deficient (*ifnar*$^{-/-}$) 129 (A129) mice were intraperitoneally injected with 0.5 mg T6167923 or DMSO (solvent control). One hour later, the mice were intradermally inoculated with 100 ng *Aa*NRP plus 250 PFU ZIKV (Group: *Aa*NRP + ZIKV and Group: *Aa*NRP + ZIKV + T6167923) or PBS plus 250 PFU ZIKV (Group: PBS + ZIKV) in their footpads. At 24 hpi, murine footpad skin was collected for flow cytometric analysis of the percentages of different myeloid cells in all the living skin cells. (**B**) Representative flow cytometric pseudocolor plots of ZIKV-infected macrophages (CD45$^+$CD11b$^+$F4/80$^+$ZIKV-E$^+$, i), DCs (CD45$^+$I-A/I-E$^+$CD11c$^+$ZIKV-E$^+$, ii) and monocytes (CD45$^+$CD11b$^+$Ly6C$^+$Ly6G$^-$ZIKV-E$^+$, iii) in murine footpad skin at 24 hpi. This data is the representative gating of ZIKV-infected myeloid cells in Fig. 5C. (**C–H**) *Aa*NRP-mediated enhancement of dengue virus 2 (DENV2) infection in A129 mice. (**C**) Schematic of the study design. Three-week-old female A129 mice were intradermally injected with 3000 PFU of DENV2 plus PBS (DENV2 + PBS) or 3000 PFU of DENV2 plus 100 ng *Aa*NRP (DENV2 + *Aa*NRP) in the hind footpad with a fine-tipped syringe. (**D**) DENV2 load in murine peripheral blood plasma. Murine tail blood was collected for viral load detection by FFU assay. The DENV2 loads were shown as FFU/ml blood plasma. (**E–G**) DENV2 load in the murine footpad (**E**), lymph node (**F**), and spleen (**G**) at 48 hpi. DENV2 loads were measured by FFU assay and shown as FFU/footpad, FFU/lymph node, or FFU/mg spleen tissue. (**H**) Survival curves of the mice inoculated with DENV2 + PBS ($n = 7$) or DENV2 + *Aa*NRP ($n = 7$). (**A**) Data are expressed as the mean ± SEM and each dot represents an individual mouse. The one-way ANOVA and multiple *t* tests were used for statistical analyses. (**D–G**) Data are expressed as the median and each dot represents an individual mouse. The two-way ANOVA (**D**) and Mann–Whitney tests (**E–G**) were used for statistical analysis. (**H**) The log-rank test was used to compare the survival curves. All experiments were reproduced at least twice. *$p < 0.05$, **$p < 0.01$, ***$p < 0.001$, ****$p < 0.0001$, n.s. not significant.

© The Author(s)

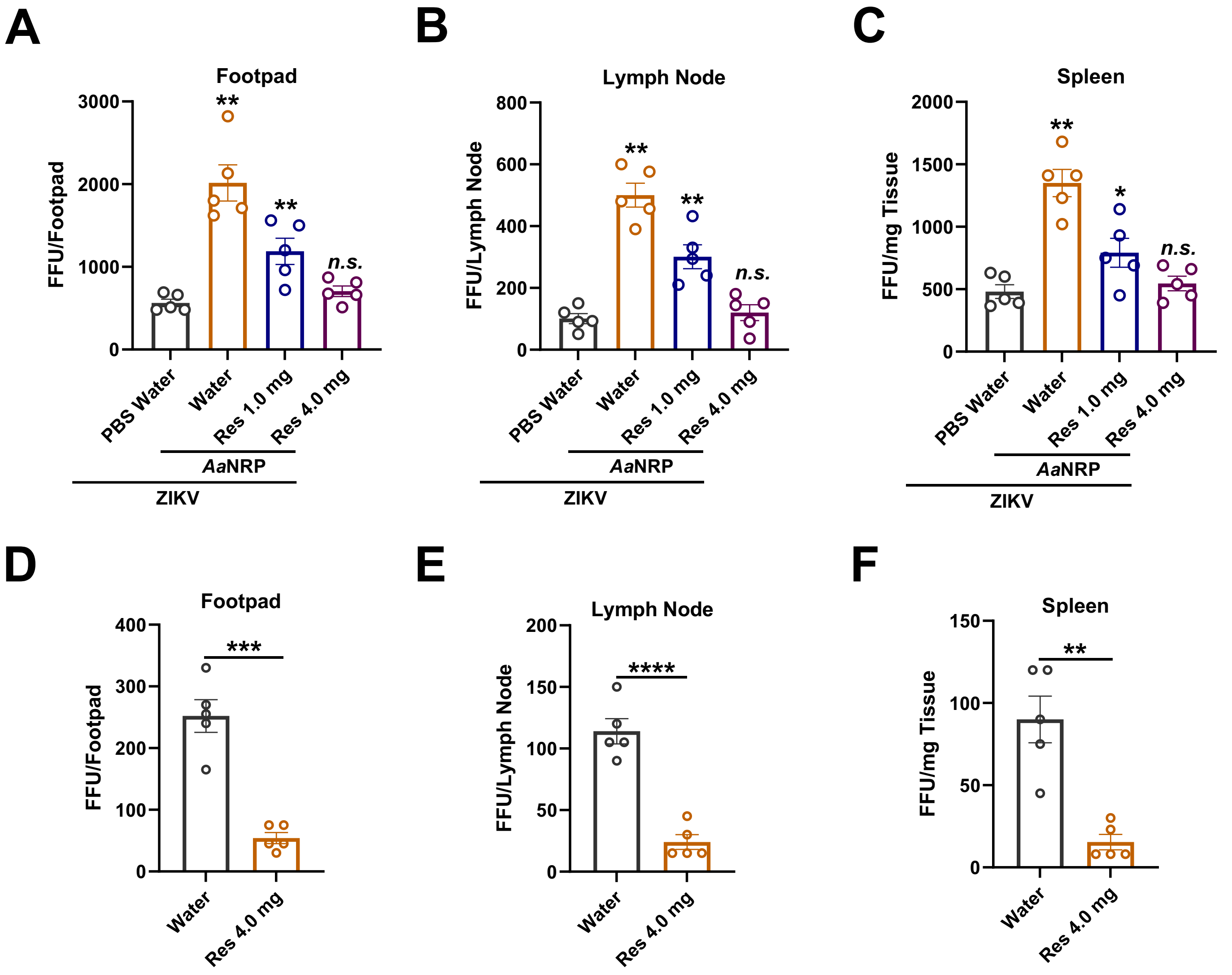

Figure EV5. Oral gavage of resveratrol relieves *Aa*NRP-promoted ZIKV infection in A129 mice, related to Fig. 7.

(**A–C**) ZIKV loads in the footpads (**A**), lymph nodes (**B**), and spleens (**C**) of mice intradermally inoculated with ZIKV at 48 hpi. Six-week-old A129 mice were orally administered water or 1.0 mg or 4.0 mg resveratrol (Res) once daily for 14 days. Afterward, the resveratrol-administered mice were intradermally injected with 100 ng *Aa*NRP + 250 PFU ZIKV immediately after the last dose of resveratrol, while half of the water-administered mice were intradermally injected with PBS + 250 PFU ZIKV as the negative control, and the remaining half were intradermally injected with 100 ng *Aa*NRP + 250 PFU ZIKV as the positive control. At 48 hpi, murine footpads, draining lymph nodes, and spleens were sampled for ZIKV load detection by FFU assay. Viral loads are shown as FFU/footpad, FFU/lymph node, or FFU/mg spleen tissue. (**D–F**) ZIKV loads in the footpads (**D**), lymph nodes (**E**), and spleen (**F**) of mice bitten by ZIKV-infected mosquitoes at 48 hpi. Six-week-old A129 mice were orally administered 4.0 mg/200 μL resveratrol (Res) or 200 μL sterile water once daily for 14 days. Afterward, each A129 mouse was bitten by three ZIKV-infected mosquitoes immediately after the last dose of resveratrol or water. At 48 hpi, murine footpads, lymph nodes, and spleens were sampled for ZIKV load detection by FFU assay. (**A–F**) Data are expressed as the mean ± SEM and each dot represents an individual mouse. The one-way ANOVA and multiple *t* tests (**A–C**) and unpaired *t* test (**D–F**) were used for statistical analyses. All experiments were reproduced at least twice. *$p < 0.05$, **$p < 0.01$, ***$p < 0.001$, ****$p < 0.0001$, n.s. not significant.

© The Author(s)
